# Transcriptional profiling of MnSOD-mediated lifespan extension in *Drosophila *reveals a species-general network of aging and metabolic genes

**DOI:** 10.1186/gb-2007-8-12-r262

**Published:** 2007-12-09

**Authors:** Christina Curtis, Gary N Landis, Donna Folk, Nancy B Wehr, Nicholas Hoe, Morris Waskar, Diana Abdueva, Dmitriy Skvortsov, Daniel Ford, Allan Luu, Ananth Badrinath, Rodney L Levine, Timothy J Bradley, Simon Tavaré, John Tower

**Affiliations:** 1 Molecular and Computational Biology Program, Department of Biological Sciences, University of Southern California, Los Angeles, CA 90089-1340, USA; 2 Department of Ecology and Evolutionary Biology, University of California, Irvine, CA 92717, USA; 3 Laboratory of Biochemistry, National Heart, Lung, and Blood Institute, Bethesda, MD 20817-6735, USA; 4 Department of Pathology and Laboratory Medicine, Childrens Hospital Los Angeles, Keck School of Medicine, University of Southern California, Los Angeles, CA 90089-9034, USA; 5 Department of Oncology, University of Cambridge, Cambridge CB2 2XZ, UK

## Abstract

**Background:**

Several interventions increase lifespan in model organisms, including reduced insulin/insulin-like growth factor-like signaling (IIS), FOXO transcription factor activation, dietary restriction, and superoxide dismutase (SOD) over-expression. One question is whether these manipulations function through different mechanisms, or whether they intersect on common processes affecting aging.

**Results:**

A doxycycline-regulated system was used to over-express manganese-SOD (MnSOD) in adult *Drosophila*, yielding increases in mean and maximal lifespan of 20%. Increased lifespan resulted from lowered initial mortality rate and required MnSOD over-expression in the adult. Transcriptional profiling indicated that the expression of specific genes was altered by MnSOD in a manner opposite to their pattern during normal aging, revealing a set of candidate biomarkers of aging enriched for carbohydrate metabolism and electron transport genes and suggesting a true delay in physiological aging, rather than a novel phenotype. Strikingly, cross-dataset comparisons indicated that the pattern of gene expression caused by MnSOD was similar to that observed in long-lived *Caenorhabditis elegans *insulin-like signaling mutants and to the xenobiotic stress response, thus exposing potential conserved longevity promoting genes and implicating detoxification in *Drosophila *longevity.

**Conclusion:**

The data suggest that MnSOD up-regulation and a retrograde signal of reactive oxygen species from the mitochondria normally function as an intermediate step in the extension of lifespan caused by reduced insulin-like signaling in various species. The results implicate a species-conserved net of coordinated genes that affect the rate of senescence by modulating energetic efficiency, purine biosynthesis, apoptotic pathways, endocrine signals, and the detoxification and excretion of metabolites.

## Abstract 2

Transcriptional profiling of MnSOD-mediated life-span extension in Drosophila identifies a set of candidate biomarkers of aging, consisting primarily of carbohydrate metabolism and electron transport genes.

## Background

Reactive oxygen species (ROS) such as superoxide, hydrogen peroxide, and hydroxyl radical are produced as byproducts of normal cellular metabolism. These ROS, especially hydrogen peroxide, are participants in cellular signaling pathways [1]. In addition, ROS can damage macromolecules and this process is implicated in human aging and disease [2]. Among the most important regulators of ROS levels are the superoxide dismutase (SOD) enzymes [3,4]: Cu/ZnSOD in the cytoplasm and outer mitochondrial space, and MnSOD exclusively in the inner mitochondrial space. Superoxide is converted to hydrogen peroxide (H_2_O_2_) and O_2 _by SOD. Peroxiredoxins and abundant catalase enzyme then scavenge the hydrogen peroxide, converting it to molecular oxygen and water. In *Drosophila*, the correlation between oxidative stress and aging is well established as demonstrated by increased levels of 8-oxo-guanine and protein carbonyls with age [5,6], and the induction of oxidative stress response genes [7-10]. Furthermore, *Drosophila *with mutated Cu/ZnSOD or MnSOD have a reduced lifespan [9,11-13] whereas tissue-specific [14] or conditional [15,16] over-expression of SOD enzymes can result in increased longevity.

Previously, the conditional transgenic system ('FLP-*out*') based on yeast FLP recombinase was used to induce the over-expression of MnSOD enzyme in adult *Drosophila *[17]. With FLP-*out*, a brief heat pulse triggered the rearrangement and subsequent expression of a *MnSOD *transgene throughout the adult lifespan, and longevity was increased in proportion to the increase in MnSOD enzyme activity. Here, a doxycycline (DOX)-regulated promoter system ('tet-on') [18] was used to induce MnSOD, thereby eliminating the confounding effect of the heat pulse and allowing for more sensitive assays. The increased sensitivity of this system was exploited to assay the effects of moderate MnSOD over-expression on mortality rates, metabolic rates, stress-resistance, and global patterns of gene expression.

Decreased signaling through the insulin/insulin-like growth factor-like signaling (IIS) pathway results in lifespan extension in the nematode, *Drosophila*, and mouse [19-21]. In *Drosophila *and *Caenorhabditis elegans*, lifespan can be increased by the IIS-target transcription factor FOXO/DAF-16. Assay of the transcriptional response to reduced IIS signaling in *C. elegans *has identified genes that are up-regulated, including those encoding MnSOD (*sod-3*) [22], and heat shock proteins (*hsp-16*) [23,24] as well as genes that are down-regulated, such as those encoding insulin-like peptides (ILPs; *ins-7*) and guanylyl cyclase (*gcy-18*) [23]. Several of the genes thought to be regulated by DAF-16 have, in turn, been found to have effects on lifespan, such as the *hsp *genes, suggesting that they might mediate part of the lifespan extension resulting from reduced IIS signaling [23-26]. Lifespan extension via reduced IIS signaling in *C. elegans *requires autophagy pathway components [27] and interacts with the heat shock factor pathway to control protein aggregate clearance [28]. Despite this progress in the identification and characterization of genes acting downstream of FOXO, the mechanism of lifespan extension by IIS has not yet been fully elucidated.

Previous genome-wide studies have identified genes that are up- and down-regulated during *Drosophila *aging [29], including tissue-specific patterns [30]. Additionally, cross-species comparisons of genome-wide expression patterns during aging have been used to search for species-general and species-specific signatures of aging [31,32]. Notably, the expression profiles of aging in *C. elegans *and *D. melanogaster *were found to show significant similarity (correlation = 0.18, *p *< 0.001) whereas a significant negative correlation was observed when the expression patterns of *daf-2 *IIS mutants were compared to those of *Drosophila *aging (correlation = -0.13, *p *<< 0.001) [31]. These results hint that similar mechanisms may mediate longevity in worms and flies, although few direct comparisons have been reported.

The data presented here demonstrate that manipulation of MnSOD expression alone is sufficient to increase lifespan through a mechanism that does not necessitate increased stress resistance, but likely involves altered metabolism. Transcriptional profiling identified candidate biomarkers of aging that consist of a set of carbohydrate metabolism and electron transport genes. Lifespan extension by MnSOD appears to proceed through a retrograde signal of increased hydrogen peroxide that involves an intricate network of genes that modulate energetic efficiency, purine biosynthesis, apoptotic pathways, endocrine signals, and the detoxification and excretion of metabolites. Cross-dataset comparisons revealed orthologous genes that are implicated in lifespan extension due to reduced IIS signaling in *C. elegans*. This implies that MnSOD up-regulation likely mediates part of the lifespan extension endowed by lowered IIS activity and identifies likely species-general effectors of longevity.

## Results

### MnSOD transgene induction prolongs *Drosophila *lifespan by rapidly reducing mortality rate

**Figure 1:**
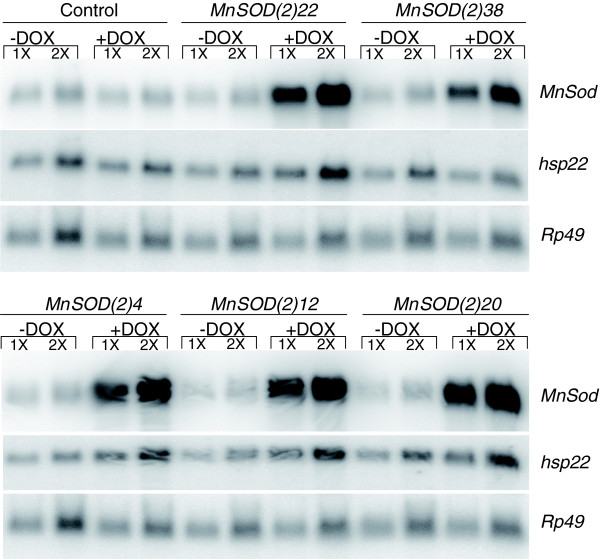
Northern analysis of MnSOD and *hsp22 *expression in control and transgenic lines. Northern analysis for controls and transgenic lines *MnSOD(2)22*, *MnSOD(2)38*, *MnSOD(2)4*, *MnSOD(2)12*, and *MnSOD(2)20 *demonstrates the induction of MnSOD transgene expression by DOX administration and the increased expression of *hsp22 *due to MnSOD over-expression. *Rp49 *represents the loading control; 1X = 5 μg RNA, 2X = 10 μg RNA.

The *Drosophila Sod2 *(MnSOD) cDNA was cloned downstream of the DOX-inducible promoter [18] and five independent single insertions were recovered on the second chromosome. In all experiments the MnSOD transgenic lines were crossed to the rtTA transactivator line (*rtTA(3)E2*) and the adult male progeny were used in assays. The rtTA transcriptional activator protein is expressed in all tissues and will activate high-level transgene expression only in the presence of DOX [18]. As such, genetically identical flies cultured in the absence of DOX represent the control for the effect of MnSOD over-expression. To control for the effect of DOX, the *rtTA(3)E2 *strain was crossed to *Or-R *wild type and the resultant hybrid progeny were used in all assays. Transgene expression was confirmed by Northern blot, and approximately 15-, 6-, 13-, 13-, and 14-fold increases in MnSOD transcripts were observed in adult flies for lines *MnSOD(2)22*, *MnSOD(2)38*, *MnSOD(2)4*, *MnSOD(2)12*, and *MnSOD(2)20*, respectively (Figure 1). No leaky expression of the transgene in the absence of DOX could be detected by Northern blot.

**Figure 2:**
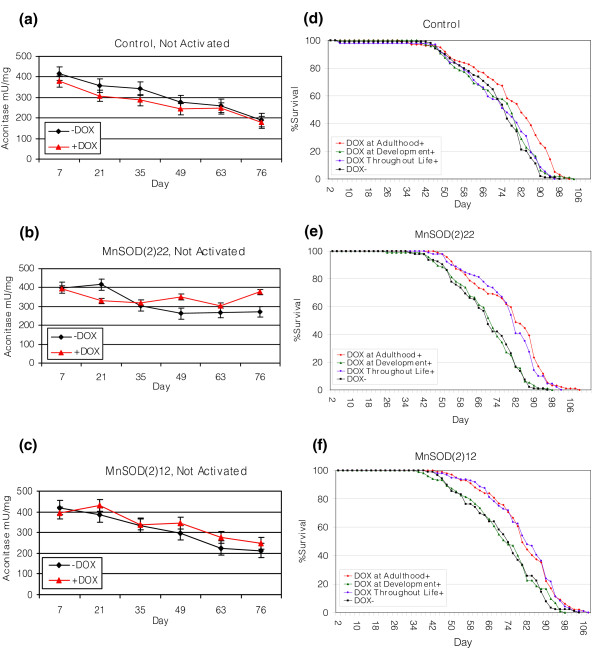
MnSOD over-expression during adulthood is necessary and sufficient for lifespan extension and does not result in increased oxidative stress. **(a-c) **Aconitase enzyme activity measured in mU/mg plotted against age for the following lines: control (a), *MnSOD(2)22 *(b), and *MnSOD(2)12 *(c). **(d-f) **The effect of timing of MnSOD induction on lifespan for control (d), *MnSOD(2)22 *(e) and *MnSOD(2)12 *(f).

**Figure 3:**
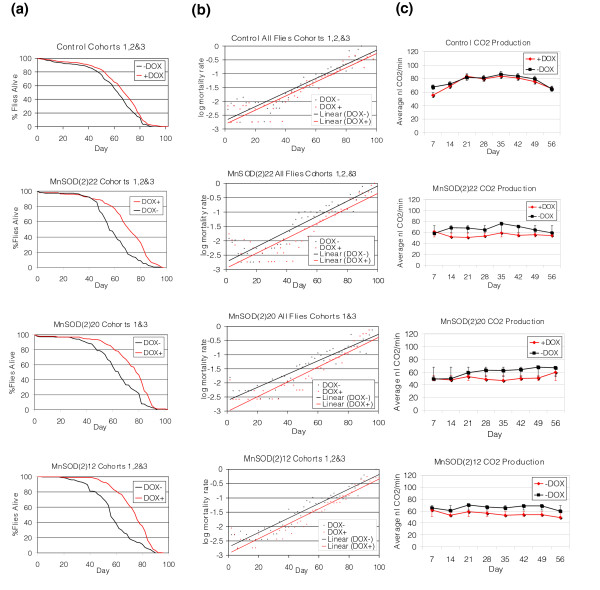
MnSOD over-expression extends *Drosophila *lifespan and alters metabolic rates. For these assays four lines were used: control, *MnSOD(2)22*, *MnSOD(2)20*, and *MnSOD(2)12*. **(a) **The percentage of animals alive is plotted against animal age. **(b) **Plots of log mortality rate against age. **(c) **CO_2 _production as measured by the average nanoliters of CO_2 _produced per minute plotted against age.

MnSOD over-expression in adults was found to be necessary and sufficient for increased lifespan, while over-expression in larvae had no detectable effect on subsequent adult lifespan (Figure 2d–f; Figure S7 and Table S1 in Additional data file 2). The effect of MnSOD over-expression on the mean, median, and 'maximum' lifespan (defined operationally here as the 90th percentile of lifespan) was assayed in multiple trials for several lines (Figure 3; Tables S2-S4 in Additional data file 2). DOX itself had no effect on maximum lifespan and a small (+8%, (95% basic bootstrap confidence interval (CI) [33], 5-11%)) but significant (log-rank test, *p *< 0.001) positive effect on mean lifespan under these conditions (Figure 3a; Tables S3-S4 in Additional data file 2). We attribute this to the fact that DOX can reduce the occasional growth of sticky bacteria on the surface of the vials, which can otherwise present a hazard for the flies. DOX also caused a dramatic decrease in the expression of immune response genes (Additional data file 3). However, other experiments indicate that such a change does not affect fly lifespan [34]. Over-expression of MnSOD significantly extended lifespan (log-rank test, *p *<< 0.001 in all cases) and yielded further increases for each line: *MnSOD(2)22*, *MnSOD(2)20 *and *MnSOD(2)12 *had increases in mean lifespan of +20% (95% basic bootstrap CI 13-20%), +20% (95% basic bootstrap CI, 16-24%) and +18% (95% bootstrap CI, 15-22%), respectively (Figure 3a; Tables S3-S4 in Additional data file 2). Maximum lifespan was increased by +13% (95% double bootstrap CI [35], 6-13%), +10% (95% double bootstrap CI, 10-14%) and +7% (95% double bootstrap CI, 5-10%), respectively. Plots of log mortality rate versus age [36] reveal that the increase in lifespan is due primarily to a rapid decrease in the initial mortality rate (within approximately 48 hours of DOX feeding), with no detectable effect on the mortality rate doubling time (Figure 3b).

### MnSOD over-expression increases neither stress resistance nor oxidative stress

In *Drosophila *and other species the IIS pathway has been shown to negatively regulate both lifespan and stress resistance, but in certain instances these outputs can be uncoupled [19,20,37-40]. MnSOD over-expression yielded no increase in resistance to the stressors hydrogen peroxide, paraquat, 100% oxygen atmosphere, or desiccation (Figures S1-S3 and Table S3 in Additional data file 2). However, MnSOD over-expression resulted in significantly diminished (log-rank test, *p *<< 0.001) thermotolerance with reductions in mean lifespan as large as -31% (basic bootstrap CI, -34% to -28%) for *MnSOD(2)22 *(Figure S4 and Tables S3-S4 in Additional data file 2).

Aconitase is an iron-sulfur cluster enzyme that is exquisitely sensitive to inactivation by oxidative stress [7], and its activity decreases during *Drosophila *aging (Figure 2a–c) [41]. MnSOD over-expression did not result in a significant change in aconitase activity (Table S8 in Additional data file 2), indicating that it does not cause an increase in oxidative stress. Thus, lifespan extension by MnSOD does not appear to involve an oxidative-stress hormesis mechanism, although the possibility that diminished thermotolerance or other types of hormesis contribute to such an effect cannot be excluded.

### MnSOD over-expression results in decreased metabolic rate

Reduced metabolic rates are associated with enhanced longevity in *C. elegans *dauer larvae as well as severe class II mutant IIS adults [42,43]. To assay the effect of MnSOD over-expression on metabolic activity, CO_2 _production was measured weekly throughout the adult lifespan in progeny from lines *MnSOD(2)22*, *20*, *12 *and controls (Figure 3c). DOX had no effect on CO_2 _production in control flies (ANOVA, *p *= 0.29) (Figure S5 and Tables S4-S6 in Additional data file 2). However, a significant decrease in CO_2 _production was observed due to *MnSOD *over-expression (ANOVA, *p *< 0.01). Averaged over the total adult lifespan, DOX caused a change of -17% (basic bootstrap CI, -21% to -13%), -16% (basic bootstrap CI, -22% to -10%) and -16% (basic bootstrap CI, -21% to -12%) in lines *MnSOD(2)22*, *MnSOD(2)20 *and *MnSOD(2)12*, respectively. There were no detectable differences in respiratory quotient (Figures S5-S6 and Tables S5-S7 in Additional data file 2). MnSOD over-expression does not simply cause a general physiological impairment, however, as these flies exhibit normal or even increased total lifetime locomotor activity (C Brown, D Grover, N Hoe, D Ford, S Tavaré and J Tower, submitted).

### MnSOD over-expression induces genome-wide transcriptional changes

**Figure 4:**
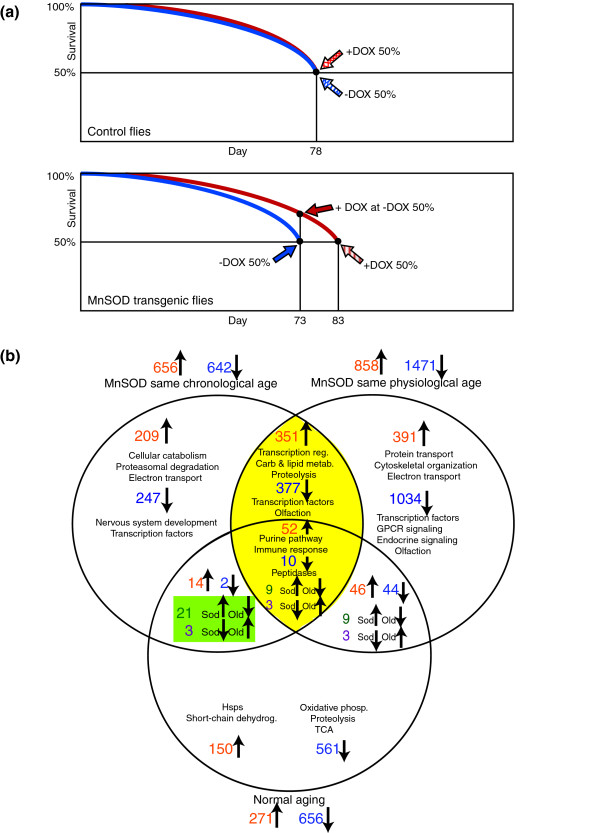
Similarities and differences in the gene expression profiles of MnSOD over-expressing and aging in *Drosophila*. **(a) **Diagram of sampling points for the transgenic and control flies used in the gene expression profiling studies. For the control, treated (+DOX) and untreated (-DOX) flies were sampled at the 50% survival of the untreated sample, which was also approximately the 50% survival point of the treated flies. For the transgenic line, untreated flies (-DOX) were sampled at their 50% survival and a sample was also taken for DOX treated (+DOX) flies at the same time point (same chronological age). An additional sample was taken for the treated flies (+DOX) at their 50% survival (same 'physiological age'). **(b) **Venn diagram depicting gene expression changes due to MnSOD over-expression and the overlap with those that occur during normal aging [10]. Yellow highlighting indicates genes whose expression levels are altered at both time points. Green shading indicates genes identified as potential biomarkers of aging. Orange or blue text denotes genes up- or down-regulated, respectively, in a given condition or in the same direction in multiple conditions. Green or purple text denotes genes up- or down-regulated, respectively, in MnSOD over-expressing flies when the direction of change is opposite in old flies. Several representative functional categorizations are noted for the various gene sets. GPCR, GTP-binding protein-coupled receptor; Hsp, heat shock protein; TCA, tricarboxylic acid cycle.

The global transcriptional response to MnSOD over-expression was assessed using Affymetrix DrosGenome1 arrays. To control for the effect of an approximately 20% delay in aging caused by MnSOD over-expression, cohorts of MnSOD transgenic flies treated with or without DOX were sampled at the same chronological age (day 73, corresponding to approximately 50% survival for -DOX flies) as well as, at the same 'physiological age' (approximately 50% survival, day 73 for -DOX flies and day 83 for +DOX flies) (Figure 4a). To control for the effect of DOX, control flies treated with or without DOX were sampled at the same chronological age (day 78, corresponding to approximately 50% survival of -DOX flies).

Assuming that MnSOD simply extends lifespan and the normal time course of gene expression changes, genes that are differentially expressed between control and long-lived flies of the same chronological age should include both the targets of MnSOD as well as potential biomarkers of aging that scale with 'physiological age'. Here, biomarkers would represent genes that normally increase or decrease in expression during aging, but have had their time course delayed by approximately 20%. At the same 'physiological age', gene expression changes should include both the targets of MnSOD as well as any alterations that do not simply represent a delay in normal aging patterns, such as genes whose expression scales with chronological age. Genes whose expression is altered (in the same direction) at the same chronological age and the same 'physiological age' should represent the primary true targets of MnSOD.

Transcriptional profiling was used to determine the extent to which the data match or depart from this simple predicted pattern. In flies of the same chronological age, MnSOD over-expression caused the up-regulation of 656 genes, and the down-regulation of 642 genes, while at the same 'physiological age' MnSOD resulted in 858 and 1,471 genes being up- and down-regulated, respectively (Figure 4b and Additional data file 4). In line with the prediction that these genes include the true targets of MnSOD, none was found to have opposing patterns of expression between the two sampling time points, while 412 and 390 were up- or down-regulated in both cases. A number of genes were only differentially expressed at one of the time points assayed. For example, of the 656 and 642 genes up- and down-regulated at the same chronological age, 244 and 252, respectively, were not identified as differentially expressed at the same 'physiological age'. Such genes may consist of both potential biomarkers of aging as well as true MnSOD targets that are not detected at the later time point because they demonstrate complex time-dependent modes of regulation. Likewise, 446 and 1,081 genes were identified as up- and down-regulated, respectively, when flies were sampled at the same 'physiological age', but not at the same chronological age. These genes may represent aspects of normal aging that are not delayed by MnSOD as well as any targets of MnSOD that have delayed induction.

These genes were mapped onto the Gene Ontology (GO) [44] classification of molecular function, biological process, and cellular compartment as a means of assessing functional profiles. Statistically overrepresented functional categories were identified using GOstat [45,46], which calculates a false discovery rate (FDR)-corrected *p *value based on a Chi-square test of whether the observed numbers of counts could have resulted from randomly distributing a particular GO term between the gene set of interest and the reference group. The statistical significance of the overlap between various gene sets was evaluated by computing the *p *value representing the probability of obtaining more than the observed number of overlaps by chance under a hypergeometric distribution, and was further assessed for several gene sets by Monte Carlo simulations.

### Candidate aging biomarkers include carbohydrate metabolism and electron transport genes

**Figure 5:**
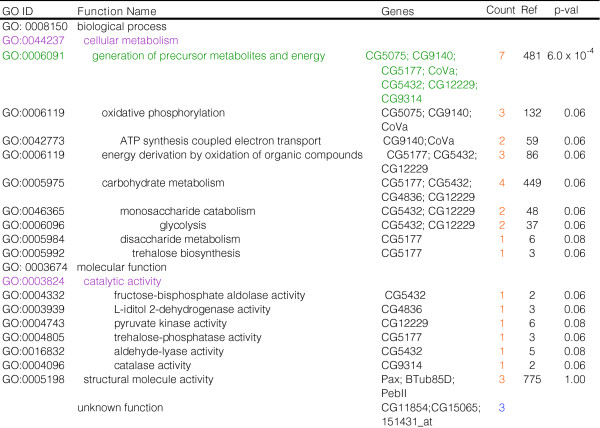
Candidate biomarkers of 'physiological age' include a highly regulated set of energy metabolism genes. GO classifications and functional overrepresentation of aging biomarkers. Orange or blue text denotes up- or down-regulated genes, respectively. 'Count' refers to the number of genes in the gene set belonging to a particular GO category. 'Ref' refers to the number of genes belonging to a particular GO category represented in the reference list (DrosGenome1 array).

One type of aging biomarker might be a gene whose expression increases (or decreases) dramatically due to aging. An intervention that delays aging should delay the time course of gene induction, and such a biomarker would then be scored as up-regulated (or down-regulated) by the intervention. Of the 244 genes that are up-regulated in long-lived versus control flies of the same chronological age, but that are not altered in flies of the same 'physiological age', 21 (approximately 9%) have opposing patterns of expression to that of normal aging [10]. The *p *value associated with the null hypothesis that this overlap occurred by chance suggests rejection of the null in support of the alternative hypothesis that the overlap is non-random (*p *< 0.005; Additional data file 5). Examination of GO annotations revealed that this suite of candidate biomarkers is enriched for genes involved in the generation of precursor metabolites and energy (GO: 006091; *p *< 0.002), such as those encoding the glycolytic enzymes pyruvate kinase (CG12229), fructose-bisphosphate aldolase (*delilah*), trehalose-phosphatase (CG5177), and L-iditol 2-dehydrogenase (CG4836) (Figure 5). Several genes involved in electron transport chain were also identified, including cytochrome-c oxidase subunit Va (*CoVa*) and NADH dehydrogenase (ubiquinone; CG9140) as was *kitty *(CG9314), which encodes a protein with predicted catalase activity. Three additional genes of unknown function (CG11854, CG15065, 151431_at) were down-regulated due to MnSOD over-expression, but up-regulated during normal aging. Thus, out of 496 changes in gene expression observed in long-lived MnSOD over-expressing flies, 24 (approximately 5%) were in the opposite direction to a change observed for those same genes during normal aging [10] (Figure 4b), consistent with a true delay in physiological aging.

### The targets of MnSOD over-expression share features with normal aging patterns

Genes that are differentially expressed between control and long-lived flies at both time points should represent the true targets of MnSOD. Surprisingly, a significant number of these genes were found to exhibit a similar change in expression during normal aging, being up-regulated in both conditions (*p *<< 0.001). Specifically, 52 genes exhibited this pattern and this list was enriched for genes involved in the defense response (*p *< 0.002), including those involved in the immune response (*AttB*, *Rel*, *Im2*, *PGRP-SD*, *PGRP-LB*, *TepII*), stress response (*hsp90*), and detoxification (*GstE1*, CG5224). Additionally, there was enrichment for genes involved in amino acid metabolism (*p *< 0.05) and aromatic compound metabolism (*p *< 0.001), such as purine (*ade2*, *ade3*, *ade5*, *CG11089*, *CG66657*), folate (*pug*, *Nmdmc*), and pyrimidine (CG8353, CG17224) metabolism genes. Ten other genes were found to be down-regulated in both conditions. Although long-lived MnSOD over-expressing flies did not demonstrate an oxidative-stress hormesis response, the fact that so many gene expression changes were common to normal aging and involved in organismal defense raises the possibility of a more general hormesis-like mechanism.

A significant number of genes altered between control and long-lived flies of the same 'physiological age', but not the same chronological age, were also found to share the same pattern of expression during normal aging and may represent aspects of normal aging that are not delayed by MnSOD (Figure 4b). The 46 genes up-regulated in this set (*p *<< 0.001) included several immune response genes (*AttA*, *Drs*, *Def*, *IM1*), cytochrome P450s (*Cyp6a9*, *Cyp6a13*, *Cyp28a5*) as well as genes encoding the heat shock proteins Hsp26, Hsp68, and Hsp22. Increased *hsp22 *mRNA levels in response to MnSOD over-expression were also confirmed by Northern blot analysis (Figure 1). Amongst the 44 down-regulated genes (*p *= 0.70), many encode peptidases, including seven of the *Jonah *genes and the accessory gland-specific peptide genes *Acp62F *and *Acp36DE*. Genes altered in MnSOD over-expressing flies at the same 'physiological age' (but not chronological age) also included many (391 up-regulated and 1,034 down-regulated) that are not normally altered with age (Figure 4b). Interestingly, the up-regulated genes include ones implicated in longevity determination via the IIS pathway, such as the phosphoinositide 3-kinase (PI3K) genes *Pi3K21B *and *Akt1*, as well as *Rheb*, *d4E-BP *(*Thor*), and the *Drosophila *JNK homologue *bsk *[47]. Also notable was the up-regulation of the gene encoding HMG coenzyme-A synthase, an enzyme implicated in juvenile hormone biosynthesis and recently linked to the IIS pathway [48]. The gene encoding the ecdysone receptor (EcR) was down-regulated in MnSOD over-expressing flies relative to controls of the same 'physiological age' along with numerous other genes involved in endocrine activity, such as ecdysteroid hydroxylase (*sad*), ecdysone-induced genes (*Eip74EF*, *Eig71Ec*, *Edg84A*, *ImpE1*), insulin-like peptide-4 (*Ilp4*), and the neuropeptides (*Nplp4*, *Nplp3*). The *Drosophila *gene *sarah *(CG6072) was also up-regulated in long-lived versus control flies of the same 'physiological age' and is related to the human *RCAN *gene, which is induced in response to hydrogen peroxide and, in turn, regulates calcinuerin and oxidative stress resistance [49]. Also included amongst this gene set were genes encoding 14 odorant receptors, 5 gustatory receptors, and 3 odorant binding proteins, and 2 additional genes encoding proteins containing an odorant binding protein domain (IPR004272). That so many genes of this class were down-regulated is particularly intriguing since olfaction has been shown to negatively regulate lifespan [50,51]. A subset of these genes was also found to be down-regulated in experimental versus control flies of the same chronological age. It is possible that certain genes were not detected at the earlier time point because they display complex patterns of expression over time that could involve delayed induction (repression) and responses to other signals that cannot be explained by two time points. Additional data file 6 gives the categorization of the gene expression differences between MnSOD over-expressing flies and controls sampled at the same 'physiological age' into these gene sets.

### MnSOD likely mediates gene expression changes via a retrograde signal to the nucleus

MnSOD over-expression alters the expression patterns of genes belonging to a variety of functional classes. The most likely means by which MnSOD effects gene expression changes is via a retrograde signal to the nucleus that is mediated by hydrogen peroxide [52]. Hydrogen peroxide is the most stable and diffusible ROS signaling molecule and has been shown to activate various signaling cascades in mammalian cells, including c-Jun-N-terminal kinase (JNK) [53], mitogen-activated protein kinase (MAPK) [54,55], and nuclear factor kappa B (NF-κB) [56].

**Figure 6:**
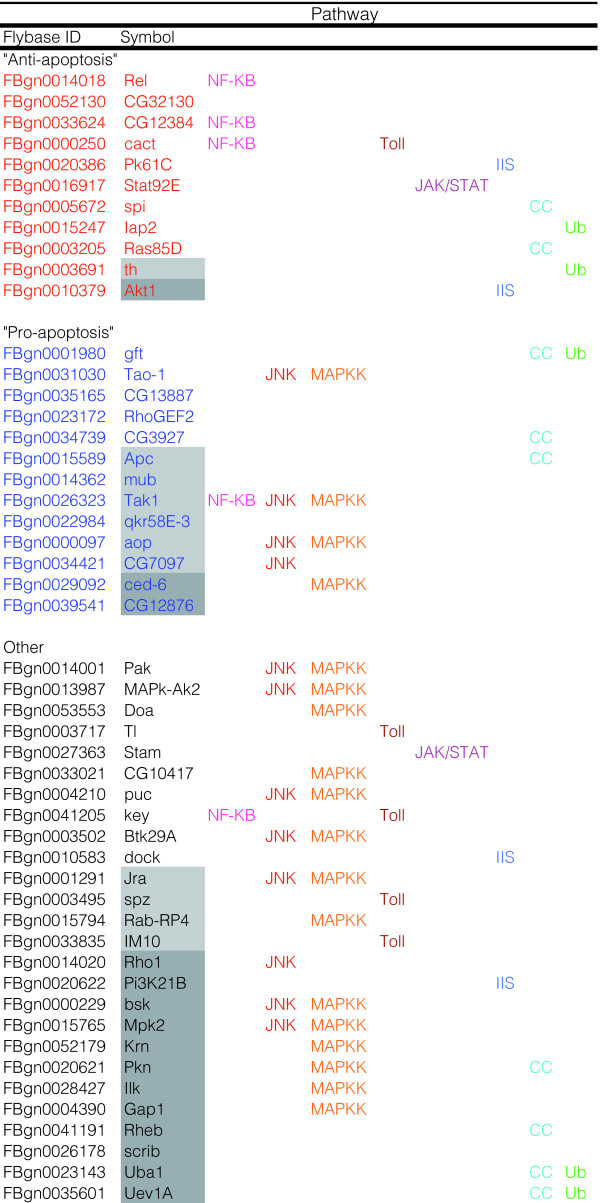
MnSOD over-expression induces numerous cellular signaling pathways. Components of signaling pathways altered by MnSOD over-expression participate in both apoptosis and cytoprotection. Light or dark grey shading indicates genes that were altered only at the first time point (same chronological age) or at the second time point (same 'physiological age'), respectively. Pathways include: NF-κB, JNK, MAPK, Toll, Janus kinase/signal transducer and activator of transcription (JAK/STAT), IIS, cell-cycle (CC), and ubiquitin mediated degradation (Ub).

In accordance with hydrogen peroxide functioning in this manner, many components of these pathways were up-regulated by MnSOD at both time points (with additional genes being altered at only one of the time points assayed) (Figure 6). The fact that hydrogen peroxide signals through these pathways and that MnSOD upregulates expression of pathway components suggests the existence of a positive feedback loop. In particular, components of the MAPK (seven genes), JNK (five genes), NF-κB (five genes), Toll (three genes), JAK-STAT (two genes), IIS (two genes), cell cycle (nine genes), and ubiquitin proteolytic (five genes) pathways were up-regulated, many of which mediate either 'pro-apoptotic' or 'anti-apoptotic' signals (Figure 6).

Another notable class of genes altered by MnSOD at both time points consisted of those encoding the antioxidants thioredoxin (*TrxT*), peroxidase (CG8913, *Jafrac1*), and multiple glutathione-S-transferases (GSTs; *GstE1*, CG5224, CG1681), each of which were up-regulated. The expression of numerous carbohydrate metabolism genes was up-regulated, including those encoding enzymes involved in both glycolysis and gluconogenesis, such as fructose-1,6-bisphosphatase (*fbp*), glycerol kinase (*Gyk*), lactate dehydrogenase (*Imp-L3*), and phosphoglucose isomerase (*Pgi*). Gene expression changes associated with lipid metabolism and ubiquitin mediated proteolysis were also altered. Additionally, an abundance of genes involved in purine and folate biosynthesis (*ATP-synβ*, *ade2*, *ade3*, *ade5*, CG3011, CG11089, CG17273, *pug*, *Nmdmc*) were up-regulated, as were components of the electron transport chain, such as the cytochrome P450s (*Cyp12d1-d*, *Cyp312a1*, *Cyp309a2*, *Cyp4p1 Cyp6d5*). Strikingly, *Cyp6d5 *has been reported to interact with *VhaSFD *[57], a vacuolar (V-type) H^+^-ATPase subunit previously implicated as a positive regulator of *Drosophila *lifespan [58]. Although the expression of this particular gene was not altered, that of *Vha100-1*, encoding another subunit of the V-type ATPase, was increased. The gene *eIF-4E*, encoding the eukaryotic translation initiation factor mRNA 5'cap-binding protein that functions to regulate cell growth, protein biosynthesis, and autophagic cell death [59] downstream of the TOR nutrient sensing pathway [60,61], was also up-regulated. Autophagy genes have previously been shown to be essential for IIS mutant lifespan extension and dauer development in *C. elegans *[27,62]. Up-regulation of several additional autophagy pathway component genes (*Atg8*, *Atg18*, *Cp1*, *l(2)01424*, *AGO2*, *eRF1*, *Rab7*, *Ect3*, *CecB*, CG12163, CG10992) correlated with lifespan extension by MnSOD. The expression of several genes implicated in circadian rhythm was altered by MnSOD; for example, *reg-5 *was up-regulated while *dunce *and *disco *were down-regulated. Other gene classes down-regulated by MnSOD included those that encode the transmembrane receptors *Notch *and *frizzled*, numerous peptidases, and 28 transcription factors, including *eve*, *gsb*, and *otp*. As mentioned above, a subset of olfactory and sensory perception genes was down-regulated at both time points, and this included genes encoding four odorant receptors (*Or46a*, *Or9a*, *Or22b*, *Or94b*), an odorant binding protein (*Obp85a*), and two gustatory receptors (*Gr21a*, *Gr57a*).

To determine whether MnSOD-regulated genes contain *cis*-regulatory elements that might be hydrogen peroxide responsive, the sequences 2,000 bp upstream of the transcriptional start site and the first intron were searched and enrichment detected using a stringent, two-step selection procedure. In particular, MnSOD-regulated genes were queried for hydrogen peroxide response elements (HREs) [63,64] and antioxidant response elements (AREs), which respond to hydrogen peroxide and phenolic antioxidants [65-67]. ARE-regulated genes are known to encode proteins involved in modulating the redox status of a cell, such as enzymes involved in glutathione synthesis or xenobiotic detoxification [66]. For example, a single ARE motif is required for the transcriptional up-regulation of glutathione in human HepG2 cells in response to hydrogen peroxide [65]. Sequences were also examined for the presence of the DNA replication-related element (DRE), which has been shown to be important for the transcriptional regulation of *Drosophila catalase *[68,69], the hypoxia induction factor (HIF)-1 response element to which the HIF-1 transcription factor binds in response to oxygen starvation [67], the DAF-16 binding element (DBE) [70], and the DAF-16 associated element (DAE) [23]. As shown in Table 1, the results indicate that both MnSOD up-regulated (*p *<< 0.001) and down-regulated (*p *< 0.05) genes are enriched for the HRE. Evidence was also found for overrepresentation of the ARE in both up-regulated (*p *< 0.001) and down-regulated (*p *< 0.05) genes, and for the DRE in up-regulated genes (*p *< 0.002). The DBE and DAE were also both enriched for (*p *<< 0.001) amongst up-regulated genes. In contrast, evidence for enrichment of the HIF-1 response element amongst MnSOD-regulated genes was not found. It is possible that the transcription factor regulating this response to hydrogen peroxide is the *Drosophila *c-Jun homologue, Jra, or the conserved transcription factor Nrf2, as it has previously been shown that human c-Jun and Nrf2 regulate ARE-mediated gene expression [67,71-73]. Taken together, these results indicate that genes altered in response to MnSOD over-expression are enriched for regulatory elements involved in the transcriptional response to hydrogen peroxide. This suggests that increased hydrogen peroxide as a result of MnSOD over-expression likely mediates some of the gene expression alterations observed in the data.

**Table 1:** MnSOD-regulated genes are enriched for hydrogen peroxide response elements

	HRE	ARE	DRE	DBE	DAE	HIF-RE
Ref	2,449	2,314	1,362	1,696	2,149	182
Up (mean no. of sites)	132 (2.4)	107 (2.4)	67 (1.2)	89 (1.9)	138 (2.0)	5 (1.0)
*P *value Up	4.10 × 10^-10 ^*	6.00 × 10^-5 ^*	3.12 × 10^-4†^	2.18 × 10^-6 ^*	1.00 × 10^-16 ^*	0.86
*P *value Up	4.10 × 10^-10 ^*	6.00 × 10^-5 ^*	3.12 × 10^-4†^	2.18 × 10^-6 ^*	1.00 × 10^-16 ^*	0.86
Dn (mean no. of sites)	77 (2.6)	74 (3.0)	18 (1.2)	38 (1.8)	49 (1.9)	3 (1.5)
*P *value Dn	0.038^†^	0.029^†^	0.99	0.85	0.86	0.71

For each motif, the number of genes for which the significance associated with finding that motif had a *p *value < 0.05 are listed. These values are reported for unique genes that were significantly up-reguated (Up) or down-regulated (Dn) by MnSOD in flies of both the same chronological and 'physiological age' and for the reference list (Ref), which derives from the Affymetrix DrosGenome1 array. The mean number of regulatory sites identified in the promoter region of the genes for which the motif was significant for a particular gene set is also reported (in parentheses). The significance of the enrichment for a given motif within a particular gene set is reported in the form of a *p *value:; ^† ^significant, * highly significant.

### Cross-species, cross-condition comparisons reveal shared longevity gene-expression signatures

**Figure 7:**
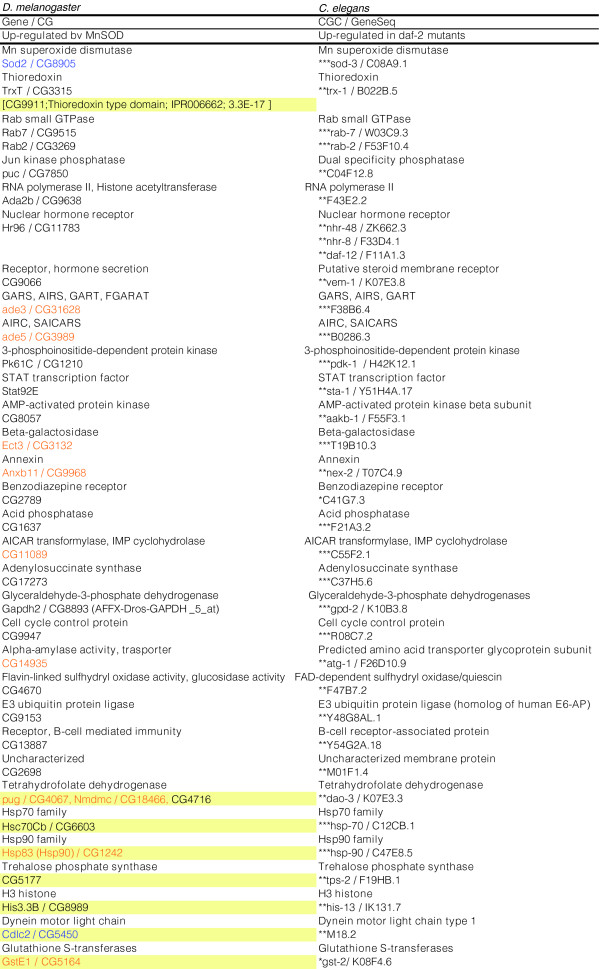
Longevity promoting genes conserved between *C. elegans daf-2 *mutants and MnSOD over-expressing *Drosophila*. *Drosophila *and *C. elegans *ortholog matches that are differentially expressed in response to MnSOD over-expression (both time points) and in *daf-2 *mutants in a *daf-16 *dependent manner. Expected values from BLASTP are indicated as follows: *5 × 10^-10 ^<*p *≤ 5 × 10^-02^, **5 × 10^-70 ^<*p *≤ 5 × 10^-10^, *** *p *≤ 5 × 10^-70^. Beige shading indicates genes that are not reciprocal best BLAST hits, but are members of the corresponding gene family. Orange or blue text indicates genes that are up-regulated or down-regulated during normal *Drosophila *aging, respectively. AIRS, 5' phosphoribosyl-5-aminoimidazole synthetase; AIRC, 5'-phosphoribosyl-5-aminoimadizole carboxylase; AICAR, 5'-phosphoribosyl-4-carboxamide-5 aminoimadizole carboxylase; FGARAT, 5'-phosphoribosyl-N-formylglycinamide amidotransferase; GARS, 5'-phosphoribosylglycinamide synthetase; GART, 5'-phosphoribosylglycinamide transformylase; SAICAR, 5' phosphoribosyl-4-(N-succinocarboxaminde)-5 amidoimidazole synthetase.

**Figure 8:**
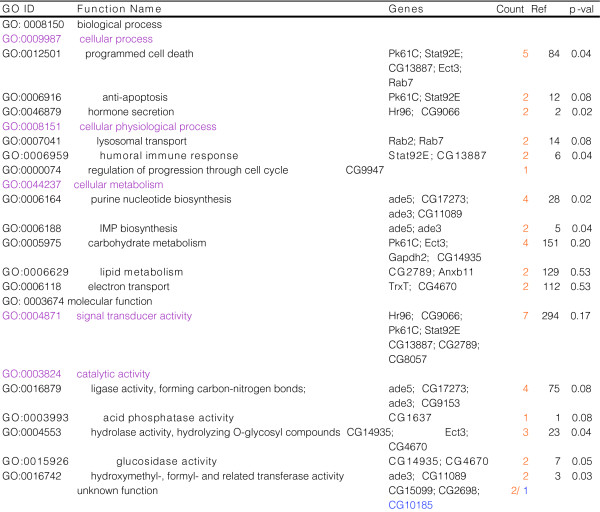
GO classifications and functional overrepresentation of conserved longevity promoting genes. Orange or blue text denotes up- or down-regulated genes, respectively. 'Count' refers to the number of genes in the gene set belonging to a particular GO category. 'Ref' refers to the number of genes belonging to a particular GO category represented in the reference list (worm-fly orthologs).

Based upon the hypothesis that longevity may be mediated by common sets of target genes that are effectors of upstream signaling pathways, and that the transcriptional targets of FOXO are likely to include direct mediators of increased longevity, the gene expression profiles resulting from MnSOD over-expression in *Drosophila *were compared to those of genes regulated by *daf-2 *in a *daf-16 *dependent manner in *C. elegans *[74,75]. Remarkably, comparison of MnSOD target genes (genes whose expression was altered at both time points) to those genes regulated by *daf-2 *in a *daf-16 *dependent manner [74] revealed 25 genes (Figure 7) out of 3,542 unique fly genes with a stringent worm ortholog that were up-regulated in both conditions, and this overlap is non-random (*p *<< 0.001; Additional data file 5). When the list of MnSOD-regulated genes was expanded to include those genes altered at the same chronological age, but not the same 'physiological age', five additional conserved genes (CG15099, *Jra*, *PHGPx*, *n-syb*, *Hrb98DE*) were identified (Additional data file 7). When genes altered at the same 'physiological age', but not the same chronological age were considered, ten additional genes were identified (*Akt1*, *Ras64B*, *Ank*, *syt*, *cib*, *ninaB*, *Cyp6a13*, CG7337, CG8112, CG3860; Additional data file 7). Of notable interest are genes known to be involved in programmed cell death (*Stat92E*, *Pk61C*, *Rab7*, *Ect3*, CG13887), insulin signaling (*Pk61C*), histone acetylation (*Ada2b*), nutrient sensing (CG8057), intracellular transport (*Rab7*, *Rab2*, CG13887), hormone secretion and the xenobiotic response (*Hr96*, CG9066), purine biosynthesis (*ade3*, *ade5*, CG17273, CG11089), carbohydrate metabolism (*Ect3*, CG14935, CG4670, *Gapdh2*), lipid metabolism (CG2789, *Anxb11*), electron transport (*TrxT*, CG4670), and ubiquitin-mediated degradation (CG9153) (Figure 8). An additional level of conservation is suggested by the observation that 6/25 genes (CG8057, CG17273, CG9066, *Stat92E*, *Ect3*, CG1637) common to the *Drosophila *MnSOD and *C. elegans daf-2 *longevity pathways are also shared by long-lived dauer worms. That these MnSOD targets are conserved from worms to flies and altered in multiple conditions that extend lifespan suggests they may play a significant role in mediating longevity.

### Xenobiotic detoxification gene expression correlates with *Drosophila *longevity

Prompted by the finding that HR96 is up-regulated by *Drosophila *MnSOD (Figures 7 and 8) and given its known role in the xenobiotic stress response, this relationship was investigated in greater detail. The data presented here were compared to those of King-Jones *et al. *[76], who examined the transcriptional response of Canton S (*CanS*) wild-type flies to phenobarbital (PB) and compared this response to those of PB-treated HR96 mutants using Affymetrix Drosophila2 arrays. Their analysis revealed 503 up-regulated and 484 down-regulated genes, respectively, in PB-treated *CanS *wild-type versus untreated flies. Of these genes, 102 were also differentially expressed between PB-treated *CanS *wild-type flies and PB-treated *Hr96 *mutants. Differences in the design of the Affymetrix DrosGenome1 arrays (used here) and the Drosophila2 arrays were accounted for by considering only those 8,636 genes that are designated 'good matches' by the manufacturer. In this way it was found that a significant portion of MnSOD up-regulated genes (59 out of 411, or 14.36%) are also involved in the *Drosophila *response to the xenobiotic PB (*p *<< 0.001; Additional data file 5). These genes include those encoding numerous detoxification enzymes, such as the P450s and GSTs, and *PHGPx*, as well as the gene encoding the juvenile hormone inducible protein JhI-26 (Additional data file 8). This list also includes folate metabolism genes and purine biosynthesis pathway components, including several conserved longevity-associated genes, such as *ade3*, *ade5*, CG11089, CG14935, and *Anxb11*, thus implicating detoxification in *Drosophila *longevity determination.

## Discussion

Here, over-expression of MnSOD in adult flies using the DOX-regulated system was found to increase mean and maximal lifespan by 20%, while over-expression during development had no detectable effect on subsequent adult life span. It should be noted that the lifespan of the controls used here (mean lifespan approximately 73 days at 25°C; Table S1C in Additional data file 2) compares favorably to the extended mutant lifespans reported for *InR *(60 days), JNK pathway (65 days), *chico *(65 days), *dTOR *(72 days) and *Methuselah *(77 days) [4,20,38,77]. Therefore, it is unlikely that MnSOD over-expression rescues some defect specific to the strains used. Preliminary data suggest that there is a limit to the amount of lifespan extension that can be achieved by over-expression of MnSOD alone: MnSOD transcript levels have been further increased by combining two MnSOD transgenic target constructs and/or by using a more active rtTA transactivator line [18], although this has so far yielded negative effects on lifespan [78]. Greater increases in life span (+40%) have been achieved by combining MnSOD with other lifespan-extending genes, such as Cu/ZnSOD [16].

Surprisingly, our studies reveal that MnSOD over-expression neither resulted in increased resistance to oxidative stress nor did it cause increased oxidative stress, and these long-lived flies exhibited diminished resistance to heat. The findings dispel the hypothesis that lifespan extension by over-expression of the antioxidant MnSOD proceeds through a mechanism that necessitates increased stress resistance. Long-lived MnSOD over-expressing flies were characterized by reduced metabolic rates as measured by CO_2 _production, but it is interesting to note that the decrease in mortality rate appeared to precede the decrease in CO_2 _production. It has been suggested [37] that longevity can be uncoupled from reduced metabolism, since O_2 _consumption was not detectably changed in long-lived *InR *mutant flies [20]. However, these assays were performed at a young time point rather than across lifespan. Furthermore, CO_2 _measurements are a more precise measure of metabolic rate than those of O_2 _consumption and so were employed in this study. Here, they indicate that metabolic rates were decreased in long-lived MnSOD over-expressing flies whereas a previous study that instead considered O_2 _consumption did not detect a difference [17]. In accordance with the measured alterations in CO_2 _production, energy metabolism genes were over-represented amongst those induced by MnSOD over-expression. This may also reflect increased requirements for energy costly processes such as endobiotic and xenobiotic detoxification or cellular maintenance that might contribute to longevity.

It is interesting to note that a subset of the genes whose expression was altered by MnSOD tended to be changed in the opposite direction by DOX alone. One conceivable explanation for this observation might be that MnSOD over-expression reduces DOX uptake or effective concentration in the flies, thereby reducing the effects of DOX on gene expression. However, since the gene expression changes due to DOX were most often smaller than those due to MnSOD, this is unlikely. Moreover, DOX-regulated expression of a LacZ reporter construct was not altered by coincident over-expression of MnSOD to a greater extent than an unrelated control transgene, suggesting that MnSOD does not affect DOX uptake (Additional data file 1, and Figure S8 in Additional data file 2). A more likely explanation for the negative correlation observed between DOX-regulated and MnSOD-regulated genes is that DOX causes a slight down-regulation of MnSOD as well as two putative Cu/Zn SOD encoding enzymes, CG9027 and *Sh3β*/CG8582. Another plausible contributing factor is the mild inhibitory effect of tetracyclines and their analogs on mitochondrial translation and proliferation [79,80], since MnSOD causes alterations in mitochondria-related genes. Despite knowledge of this small effect for over two-decades, DOX-regulated systems have still been successfully employed to study mitochondrial function in detail, including mitochondrial translation [81,82]. Thus, the combined effect of a slight reduction in MnSOD and two other Cu/Zn superoxide-dismutase encoding genes along with a decrease in mitochondrial translation can readily account for the negative correlation observed between DOX-regulated and MnSOD-regulated genes.

### Candidate aging biomarkers include carbohydrate metabolism and electron transport genes

Based upon their opposing expression pattern between control and long-lived flies of the same chronological age and that of normal aging, a set of 24 potential aging biomarkers was identified and found to consist primarily of highly regulated carbohydrate and energy metabolism genes (Figure 5). In the future, it may be possible to validate such biomarkers by examining their longitudinal expression profiles and ability to predict remaining lifespan in individual flies [10,83].

Of these potential biomarkers, CG9140 and *CoVa *are expected to participate in electron transport. This is interesting in light of the finding that electron transport chain genes are consistently diminished with age in flies, mice, and humans (but not worms) [84], suggesting that diminished expression of the electron transport pathway with age may be an important marker of 'physiological age' and supporting our findings. Three of the potential biomarkers are expected to function in nucleotide binding (CG9920, *betaTub85D*, CG5075), and two in nucleobase metabolism (CG7804, CG5075). CG9220 encodes a glucuronosyltransferase and, based on sequence similarity, may participate in protein folding, *betaTub85D *functions in microtuble-based movement, and CG5075 encodes a hydrogen-exporting ATPase. In addition, four genes comprising key regulatory components of the glycolytic pathway were represented amongst this class of potential biomarkers, including pyruvate kinase (CG12229) and fructose-bisphosphate aldolase (*delilah*) as well as genes that act in peripheral pathways, such as those encoding trehalose-phosphatase (CG5177) and L-iditol 2-dehydrogenase (CG4836). Trehalose-phosphatase catalyzes the de-phosphorylation of trehalose-6-phosphate to trehalose and orthophosphate. In insects, trehalose and glucose are the only circulating sugars found in the hemolymph. While glucose is obtained from the diet, trehalose is a key homeostatic molecule that derives from the fat body and is involved in sugar transport to peripheral tissues and energy storage [85]. This non-reducing sugar is thought to increase desiccation tolerance by preventing protein aggregation, and trehalose phosphate synthase protects *Drosophila *during anoxia [86,87]. Previously, the reduced thermotolerance of long-lived median neurosecretory-cells (mNSC)-ablated flies was attributed to lowered circulating trehalose concentrations in the hemolymph [40]. These flies exhibited an altered pattern of circulating carbohydrates, having reduced circulating trehalose (approximately 15%), increased circulating glucose (100%), and increased whole body energy stores of trehalose, glycogen, and lipids [40]. Additionally, both male and female *InR *mutants have been shown to be hyper-trehalosemic [88]. These results are in line with the finding that lifespan extension by MnSOD is characterized by diminished thermotolerance, alterations in carbohydrate metabolism gene expression, and the up-regulation of trehalose phosphatase. Furthermore, they corroborate the observation that specific carbohydrate metabolism genes are potential biomarkers of aging. Trehalose has previously been touted as a longevity-assurance sugar in *C. elegans *based upon the increased expression of trehalose phosphate synthase in *daf-2 *mutants and increased levels of trehalose in dauer larvae and IIS *age-1 *(hx546) mutants [89-91].

### The targets of MnSOD over-expression share features with normal aging patterns

An intriguing finding is that a significant number of genes (52) up-regulated by MnSOD at both time points are also up-regulated during normal aging and this list is enriched for genes involved in the defense response, such as immune response genes (*AttB*, *Rel*, *Im2*, *PGRP-SD*, *PGRP-LB*, *TepII*), heat shock proteins (*Hsp90*), GSTs (*GstE1*, CG5224), and peroxidase (CG8913). Enrichment for heterocyclic-compounds and amino acid metabolism was also found. Notably, several of the genes in this set (*ade3*, *ade5*, CG11089, *Ect3*, *Anxb11*, CG14935) were also identified as species-conserved, longevity-associated genes (Figure 7). These findings suggest that MnSOD may partially mediate lifespan extension by effecting a species-general, non oxidative-stress, hormesis response.

### MnSOD over-expression causes reduced expression of genes that negatively regulate lifespan

Endocrine signals have been demonstrated to regulate life cycles and affect aging in all higher organisms [92]. Both juvenile hormone and 20-hydroxyecdysone are decreased in IIS mutants [20]. Additionally, EcR mutant heterozygotes are long-lived [93]. This suggests that, in *Drosophila*, reduced IIS activity may extend lifespan, in part, by diminishing signaling through juvenile hormone and ecdysone. It is interesting, therefore, that the gene encoding the EcR was down-regulated in MnSOD over-expressing flies relative to controls of the same 'physiological age' along with numerous other genes involved in endocrine activity, such as ecdysteroid hydroxylase (*sad*), ecdysone-induced genes (*Eip74EF*, *Eig71Ec*, *Edg84A*, *ImpE1*), insulin-like peptide-4 (*Ilp4*), and the neuropeptides (*Nplp4*, *Nplp3*). Since MnSOD may be a downstream effector of FOXO in flies, this suggests that lifespan extension in IIS mutants may involve a MnSOD-mediated reduction in signaling through the EcR.

The finding that numerous genes involved in olfaction and gustation are down-regulated by MnSOD is also intriguing. Olfactory and gustatory neurons are known to negatively regulate lifespan in *C. elegans *[50,51], and it was recently shown that in *Drosophila*, exposure to nutrient-derived odors reduces lifespan extension caused by dietary restriction [94]. Furthermore, mutation in the *Drosophila Or83b *receptor resulted in defective olfaction, altered metabolism, increased stress resistance, and lifespan extension [94].

### Cross-species, cross-condition comparisons reveal shared longevity gene-expression signatures

The gene expression profiles in *Drosophila *upon MnSOD over-expression were compared to the expression profiles that result from long-lived *C. elegans daf-2 *insulin receptor (InR)-like mutants and dauer larvae [74,75]. Strikingly, this comparison revealed numerous genes with similar expression patterns that are conserved between the worm and fly and likely represent longevity promoting genes (Figure 7, Additional data file 7). This is in contrast to a recent study [95] that identified conservation only at the process level, but not the gene level. Amongst the genes identified are those involved in the purine biosynthetic pathway, programmed cell death, intracellular protein transport, ribosome biogenesis, insulin signaling, and hormone secretion. Of particular interest is the finding that an energy sensing AMP-activated protein kinase (CG8057) and the nuclear hormone receptor HR96, a xenobiotic stress sensor, are up-regulated by MnSOD. Gene expression profiling of individual nematodes identified the AMP-activated protein kinase (AMPK) *beta *subunit as a gene that differentiates wild-type and *daf-2 *mutants with respect to age [96]. Recently, over-expression of the AMPK *alpha *subunit, *aak-2*, in *C. elegans *was shown to increase longevity, and lifespan extension by mutation of *daf-2 *or *sir-2.1 *over-expression was found to be dependent on *aak-1*. It is notable, therefore, that, in *Drosophila*, CG8057, which encodes an AMPK, is up-regulated by MnSOD over-expression as well as reduced IIS signaling, and concomitantly down-regulated upon yeast re-feeding after dietary restriction [97]. Thus, the profiles observed in response to lifespan altering interventions in *Drosophila *support the view that as in *C. elegans*, AMPK coordinates metabolism at an organismal level by integrating positive and negative cues to maintain cellular ATP levels [98]. In *C. elegans*, the HR96 homologue, DAF-12, acts at the intersection of pathways that regulate larval diapause, development, stress responses, and adult longevity [99,100]. While a similar role for HR96 in mediating *Drosophila *longevity has not been previously reported, we find further support for this connection by demonstrating that a significant portion of genes regulated by MnSOD are also similarly altered in response to xenobiotic stress induced by phenobarbital. This finding is of particular interest since McElwee and colleagues [74,75] have previously reported that the phase I and phase II class of enzymes involved in xenobiotic detoxification are shared between *C. elegans *dauers and *daf-2 *mutants. Several of the species-conserved, potential longevity promoting genes are described in further detail in Additional data file 10.

### MnSOD-regulated targets downstream of dFOXO

The cross-species, cross-condition comparison described above was aimed at identifying genes and processes that broadly mediate lifespan and, hence, are robust signatures of longevity mechanisms. However, certain downstream targets of dFOXO may have been missed by a comparison of stringent orthologs. In order to identify species specific MnSOD-regulated targets that act downstream of dFOXO as well as potential lifespan promoting mechanisms that might be unique to *Drosophila*, the transcriptional profile of MnSOD over-expression was compared to those resulting from altered insulin signaling in *Drosophila*. These comparisons are described in Additional data file 10.

### MnSOD-mediated mitochondria to nucleus signaling and crosstalk with the IIS pathway

Taken together with results from *C. elegans*, the data suggest a model in which MnSOD is a direct transcriptional target of the FOXO transcription factor and MnSOD catalyzed detoxification of superoxide results in increased intracellular hydrogen peroxide levels that mediate numerous signaling events. Based on kinetic arguments, it has been suggested that it is unlikely that over-expression of MnSOD could significantly increase cellular hydrogen peroxide levels [101]. One way to reconcile these observations is to suggest a localized region of hydrogen peroxide increase such as might be afforded by physical proximity between the mitochondria and nucleus [102]. Although it is not possible to rule out decreased superoxide as the retrograde signal at this time, that hydrogen peroxide is the relevant signal is supported by previous studies demonstrating that catalase over-expression on its own, in combination with Cu/ZnSOD [15] or MnSOD [17], has neutral or slightly negative effects on lifespan. Additionally, previous studies in cultured mammalian cells suggest that MnSOD-mediated growth suppression is due to elevated hydrogen peroxide levels resulting in oxidative environments in the mitochondria and subsequently in the cytoplasm [103]. It is also of interest to note that hydrogen peroxide and the antifungal para-hydroxymethyl-benzoic acid are reported to favor survival of flies restricted to a sugar only diet [104].

In further support of a hydrogen peroxide signal, there is a highly significant overlap in the genes altered by MnSOD over-expression and those altered upon direct stimulation with 3% hydrogen peroxide (C Curtis, G Landis, D Skvortsov, D Abdueva, K Tozer, J Tower and S Tavaré, in preparation). Specifically, 312 (*p *value < 4.0 × 10^-42^) and 260 (*p *value < 4.3 × 10^-43^) genes were also up-regulated upon hydrogen peroxide treatment as well as in MnSOD over-expressing flies of the same physiological and chronological age, respectively. A significant overlap was also found for genes down-regulated by hydrogen peroxide and down-regulated upon MnSOD over-expression in flies of the same physiological (216; *p *value < 0.003) and chronological (103; *p *value < 0.003) age, respectively, although to a lesser extent than genes up-regulated in both conditions.

The comparison of MnSOD-regulated gene expression changes to *daf-16 *dependent changes in IIS mutants suggests that MnSOD modulates the expression of numerous genes downstream of FOXO. It is interesting to note, therefore, that some of these targets contain both hydrogen peroxide responsive *cis*-regulatory elements, such as the HRE and ARE, as well as DAF-16 related elements, such as the canonical DBE and the DAE. This raises the possibility that such targets might be regulated both by MnSOD, through hydrogen peroxide signaling, and FOXO (Additional data file 9). Other genes that lack both the DBE and DAE might be indirect effectors of FOXO that are regulated by MnSOD.

**Figure 9:**
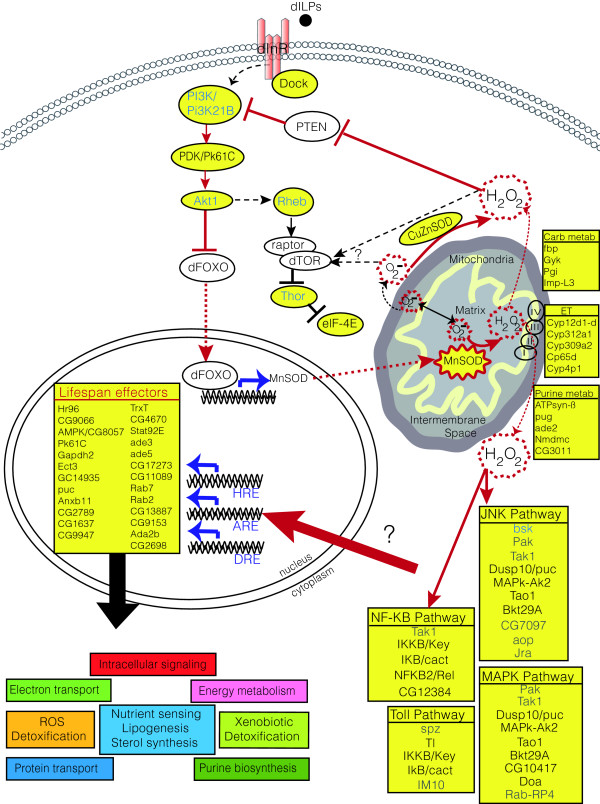
Proposed mechanism for MnSOD-mediated mitochondria to nucleus signaling and crosstalk with the IIS pathway. The data suggest a model in which MnSOD catalyzed detoxification of superoxide results in increased intracellular hydrogen peroxide levels that mediate various signaling events. Such events include the activation of the JNK and NF-κB pathways. Pathway components that demonstrate increased expression due to MnSOD over-expression are highlighted in yellow. Note that genes up-regulated at both time points are indicated by black text, those up-regulated only at the first time point assayed are indicated by grey text, whereas those up-regulated only at the later time point are denoted by blue text. Solid lines indicate direct interactions, dashed lines indicate indirect interactions, dotted lines indicate translocation events, and '?' indicates hypothetical or speculative elements. The proposed retrograde signal from the mitochondria to the nucleus mediated by hydrogen peroxide is shown in red. Numerous genes are up-regulated as a result of these signaling events and some were also identified as being similarly altered in long-lived *C. elegans *IIS mutants, suggesting their role as species-general lifespan effectors. These genes are indicated as are the biological processes that they contribute to. Hydrogen peroxide reversibly inhibits PTEN [105], an upstream inhibitor of IIS, resulting in activation of phosphoinositide 3-kinase (PI3K) signaling. In accordance with this, *Pk61C *gene expression levels are up-regulated as are some downstream components of the IIS pathway in response to MnSOD over-expression. Increased IIS activity results in dFOXO inactivation and since MnSOD may be a direct transcriptional target, this suggests that feedback regulation may occur. The proposed feedback loop between MnSOD and the IIS pathway is also shown in red. Crosstalk between TOR and its binding partner, raptor, with the mitochondrion has been suggested [111], although the molecular basis has not been elucidated. As shown here, hydrogen peroxide may participate in this mechanism. ET, electron transport; ILP, insulin-like peptide.

Recently, JNK has been reported to extend lifespan in *Drosophila *[47], and its activation by hydrogen peroxide may facilitate the interplay between ROS mediated apoptotic and protective signals in a pathway that also involves the NF-κB cascade and mitochondria to nucleus signaling [52] (Figure 9). Hydrogen peroxide has also been shown to reversibly inactivate purified human PTEN, a tumor suppressor and upstream inhibitor of insulin signaling through phosphatidylinositol (3,4,5)-triphosphate, by oxidation of the essential cysteine residue in the active site of the PTEN lipid phosphatase [105]. In further support of a role for MnSOD in the redox regulation of PTEN, it has recently been demonstrated in *Drosophila *that thioredoxin (shown here to be induced by MnSOD) inhibits PTEN through disulfide bond formation and that over-expression of human thioredoxin in fly heads resulted in increased Akt phosphorylation [106]. In accordance with these findings, the gene encoding *Drosophila *phosphoinositide dependent kinase, *Pk61C*, is up-regulated in response to MnSOD over-expression. Downstream of *Pk61C*, additional components of the IIS pathway are up-regulated, including *eIF-4E*. eIF-4E is repressed by its binding protein, Thor, a direct transcriptional target of dFOXO that mediates cues from changing environmental conditions, including starvation and oxidative stress to control cell number during development [107,108]. Increased IIS results in inactivation of FOXO by phosphorylation and exclusion from the nucleus and since MnSOD may be a direct transcriptional target of dFOXO, this suggests the possibility of negative feedback regulation between MnSOD and the IIS pathway (Figure 9). Notably, while nucleo-cytoplasmic shuttling of DAF-16 in *C. elegans *is an important component of its regulation, recent studies suggest [109] that the nuclear localization of this FOXO transcription factor may not be required for all of its activity [110]. Furthermore, it is of interest that additional components of the IIS pathway, such as *Pi3K21B*, *Akt1*, *Rheb*, and *Thor*, are up-regulated in MnSOD over-expressing flies relative to controls sampled at the same 'physiological age', but not the same chronological age. One possible explanation for this is delayed induction or complex time-dependencies in the expression patterns that might result from feedback regulation. An interesting consequence of such a feedback loop would be control of MnSOD expression levels. This may be important for maintaining redox balance and is supported by the finding that high levels of MnSOD expression are toxic [78]. An adaptive response of MnSOD expression levels to the mitochondrial redox state has previously been suggested [103]. The importance of tight regulation of MnSOD is underscored by the fact that optimal enzyme activity levels should be such that the lower limit is sufficient to remove mitochondrial superoxide, whereas the upper limit does not exceed mitochondrial hydrogen peroxide removal capacity [103]. Furthermore, the fact that in these flies lifespan is extended, while much higher-level over-expression of MnSOD is toxic [78], suggests that in these experiments hydrogen peroxide levels are being manipulated within the normal physiological range for signaling, and, therefore, are consistent with the observation that there was no obvious oxidative stress response or inactivation of aconitase enzyme.

Other pathways involved in nutrient sensing have also been shown to crosstalk with the mitochondria through feedback mechanisms. For example, TOR is implicated in regulating the balance between glycolysis and mitochondrial metabolism, although the molecular basis has yet to be elucidated [111,112]. Additionally, oxidative capacity correlates with TOR-raptor complex stability [111], suggesting that a retrograde signal from the mitochondria influences TOR activity. It is possible that hydrogen peroxide signaling participates in this mechanism.

## Conclusion

A surprising aspect of the data is that a single, albeit important, enzyme can have a profound effect on the organism's longevity, metabolic rate, and gene expression. MnSOD likely mediates some of these beneficial changes in nuclear gene expression by a retrograde signal of increased hydrogen peroxide. Lifespan extension by MnSOD appears to proceed through a regulatory response that involves an intricate network of genes, orthologs of which are implicated in lifespan extension from reduced IIS activity in *C. elegans*. This implies that part of lifespan regulation by IIS normally proceeds through MnSOD, and identifies likely species-general effectors of longevity.

## Materials and methods

### *Drosophila *strains

All *Drosophila melanogaster *strains were as described [18,113,114].

### Plasmid construction

PCR products (MnSOD-1, MnSOD-2) were obtained using a pBlue Script vector containing the MnSOD cDNA as a template [17]. MnSOD-1 was generated using primers Mn1F (5'GTCGAATAAAACGCAGATATGTTCG-3') and Mn1R (5'-CCATGGTTAAATAATCGGCGTTGAA-3'). MnSOD-2 was generated using primers MnSOD2F (5'-TGCAGTCGAATAAAACGCAGATATGTTCG-3') and MnSOD2R (5'-TTAACCATGGTTAAATAATCGGCGTTGAA-3'). Both products were generated using pfu DNA polymerase (Stratagene, San Diego, CA, USA). Products MnSOD-1 and MnSOD-2 were boiled for 10 minutes at 95°C and cooled to room temperature to generate a reannealed MnSOD gene with a *Pst*I site engineered at the 5' end and an *Eco*RI site at the 3' end. This fragment was cloned into the *Pst*I and *Eco*RI sites of USC1.0 [115] to generate the construct USC1.0-MnSOD.

### P element mediated transformation

Five independent germ-line transformants of the USC1.0-MnSOD construct (*MnSOD(2)4*, *MnSOD(2)12*, *MnSOD(2)20*, *MnSOD(2)22 *and *MnSOD(2)38*) were generated using standard methods [116], using the *y-ac-w1118 *recipient strain [117]. Southern analysis indicated the presence of single inserts for all lines.

### *Drosophila *culture and lifespan assays

*Drosophila *were cultured on standard agar/molasses/corn meal/yeast media [118]. Where indicated, flies were cultured on food supplemented to a final concentration of 240 μg/ml DOX and 64 μg/ml ampicillin, while control vials were adjusted 64 μg/ml ampicillin alone. To obtain adult flies, the *Oregon R *control strain (provided by the Bloomington *Drosophila *stock center) and MnSOD transgenic lines (*MnSOD(2)4*, *MnSOD(2)12*, *MnSOD(2)20*, *MnSOD(2)22*, *MnSOD(2)38*) were crossed to the *rtTA(3)E2 *transactivator line [18], cultured at 25°C in urine specimen bottles, and the hybrid adult male progeny resulting from these crosses were used in all experiments. Prior to eclosion of the majority of pupae, bottles were cleared of adults and newly eclosed flies were allowed to emerge over the next 48 hours. The majority of the males will have mated during this time. The males only were then removed and were designated one day old, and were maintained at 25°C at 40 flies per vial in culture vials with food. All flies were transferred every other day into fresh media unless otherwise indicated. At 4 days of age the males were split into control and experimental groups of 200 males each, with the experimental group (+DOX) placed on culture media supplemented with 240 μg/ml DOX. The number of dead flies was counted at each passage, and the number of vials was progressively reduced to maintain approximately 40 flies per vial. To calculate the mean lifespan for the experimental (+DOX) and control (-DOX) cohorts, each fly's lifespan was tabulated, the data averaged, and the mean, median and standard deviation were calculated.

### Northern analyses

Flies were cultured on plus and minus DOX for one week. RNA was isolated from male adult *Drosophila *using the RNAqueous kit (Ambion, Austin, TX, USA), fractionated on 1.0% agarose gels and transferred to GeneScreen membranes (PerkinElmer, Waltham, MA, USA). 1X = 5 mg, and 2X = 10 mg. The PCR product MnSOD-1 was used as a specific probe for the MnSOD gene. The probe for the *hsp22 *gene was generated from a genomic subclone. The loading control was *Rp49*, which encodes a ribosomal protein [119]. DNA probes were ^32^P-labelled using the Prime-It II DNA labeling kit (Stratagene). Hybridization was carried out in Church-Gilbert solution at 65°C overnight. Hybridization signals were visualized and quantified using the phospho-imager and ImageQuant software (Molecular Dynamics, Sunnyvale, CA, USA). Transcript size was determined by comparison with 1 Kb RNA ladder (Gibco-BRL, Gaithersburg, MD, USA) according to the manufacturer's instructions.

Relative RNA levels and the fold induction of transcripts were estimated from Northern blot data as follows: a box was drawn around the band for each gene transcript and intensity measured in arbitrary units using the Phosphoimager and ImageQuant. An equal size box was drawn around a region of the lane containing no bands and that value was subtracted as background. *Rp49 *loading control was quantified in the same way for each lane. Each *Rp49 *intensity value was divided by the median *Rp49 *intensity value to generate a loading correction factor for each lane. A normalized intensity value for each gene transcript was then calculated by multiplying by the *Rp49 *correction factor for that lane. This quantification was done twice for each phospho-image. The 1X and 2X Northern lanes for each RNA sample were quantified and the numbers were averaged. The resulting relative expression levels are presented in arbitrary units ± standard deviation.

#### *MnSOD *probe data

*MnSOD(2)4 *-DOX = 8,194,986 ± 282,073, +DOX = 105,246,261 ± 4,133,376, fold induction approximately 13. *MnSOD(2)12 *-DOX = 9,130,631 ± 509,194, +DOX = 118,636,517 ± 4,449,685, fold induction approximately 13. *MnSOD(2)20 *-DOX = 9,786,260 ± 612,061, +DOX = 135,629,576 ± 18,497,585, fold induction approximately 14. *MnSOD(2)22 *-DOX = 14,089,283 ± 1,569,894, +DOX = 210,419,748 ± 2,779,774, fold induction approximately 15. *MnSOD(2)38 *-DOX = 10,211,365 ± 1,113,851, +DOX = 57,845,424 ± 2,076,761, fold induction approximately 6.

#### *hsp22 *probe data

*MnSOD(2)4 *-DOX = 2,077,692 ± 33,078, +DOX = 5,122,305 ± 576,188, fold induction approximately 2.5. *MnSOD(2)12 *-DOX = 2,565,603 ± 274,446, +DOX = 5,256,513 ± 91,162, fold induction approximately 2.0. *MnSOD(2)20 *-DOX = 3,479,575 ± 346,431, +DOX = 5,679,243 ± 710,345, fold induction approximately 1.6. *MnSOD(2)22 *-DOX = 4,684,510 ± 14,391, +DOX = 9,017,229 ± 1,220, fold induction approximately 1.9. *MnSOD(2)38 *-DOX = 2,528,625 ± 100,005, +DOX = 2,288,297 ± 279,032, fold induction approximately 0.9.

### Statistical analysis of the effect of DOX-induced MnSOD over-expression on lifespan, stress resistance, desiccation, metabolism, and aconitase levels

Additional data file 1 includes a complete description of the analyses performed and Additional data file 2 includes the results.

### Assaying the effect of MnSOD over-expression during development and adulthood

*MnSOD *males of the indicated lines were mated to *rtTA(3)E2 *virgins and their progeny allowed to develop in bottles containing food plus and minus DOX. At eclosion, age-synchronized cohorts of *MnSOD/rtTA(3)E2 *males were transferred to vials containing five flies each. Both the progeny from the -DOX and +DOX bottles were split into two groups, one in +DOX vials, and one in -DOX vials, resulting in four sets of lifespan assays of 100 males each: +DOX in adulthood only, +DOX in development only, +DOX throughout lifespan, and no DOX. The plus DOX vials and bottles included 240 μg/ml DOX. The flies were transferred into fresh vials every other day and survival was determined by counting the number of dead flies in each vial. A description of the statistical analyses performed is presented in Additional data file 1 and the results are shown in Tables S1-S4 in Additional data file 2.

### Statistical analysis of the effect of MnSOD on lifespan

The effect of DOX treatment during adulthood on mean and maximal lifespan were assessed using log-rank and Chi-squared tests, respectively (Tables S1-S3 in Additional data file 2). The effect of MnSOD over-expression on the mean, median, and the 90th percentile of lifespan was further examined by employing a bootstrap resampling scheme [33] to construct 95% confidence intervals for the ratio of the means and for the ratio of percentiles of the control and treatment populations [35,120]. Such robust methods have been shown to provide confidence intervals with coverage much closer to the nominal value than classical methods in certain instances. For the ratio of percentiles, where no simple variance estimator is known, a double-bootstrap approach was taken to estimate the variance. For each bootstrap sample (B1), an additional bootstrap sample (B2) was employed to compute estimates of û**, the sample variance of which is the bootstrap estimate of the variance of û*. For the ratio of means, four different types of equi-tailed, two-sided nonparametric confidence intervals were constructed: the normal approximation, bootstrap-t interval, the basic bootstrap interval, and the double bootstrap interval. For the ratio of percentiles, the basic bootstrap interval and the double bootstrap interval were computed. In all cases, B1 = 5,000, B2 = 1,000, and α = 0.05. Results are shown in Table S4 in Additional data file 2.

### Hydrogen peroxide survival assay

Age synchronized cohorts of adult male flies were cultured on plus and minus DOX food for one week. They were then transferred to vials containing tissue (Kimwipes) saturated with 0%, 2.5%, and 5% hydrogen peroxide in a 1% sucrose solution. The +DOX vials included 240 μg/ml DOX. The flies were transferred into fresh vials each day, and survival was determined by counting the number of dead flies in each vial.

### Paraquat survival assay

Age synchronized cohorts of adult male flies were cultured on plus and minus DOX food for one week. They were then transferred to vials containing tissue saturated with 20 mM paraquat in a 1% sucrose solution. Paraquat solutions were made fresh for each experiment, as this was found to be necessary for reproducible results. The +DOX vials included 240 μg/ml DOX. The flies were transferred into fresh vials each day, and survival was determined by counting the number of dead flies in each vial.

### 100% Oxygen survival assay

Age synchronized cohorts of adult male flies were cultured on plus and minus DOX food for one week. They were then placed in an enclosed chamber with 100% oxygen gas flow [10], transferred into fresh vials each day, and survival was determined by counting the number of dead flies in each vial.

### Thermal stress survival assay

Age synchronized cohorts of adult male flies were cultured on plus and minus DOX food for one week. They were then placed in an incubator at 34°C, transferred onto fresh food each day, and survival was determined by counting the number of dead flies in each vial.

### Desiccation resistance assay

Age synchronized cohorts of adult male flies were cultured on plus and minus DOX food for one week, after which the flies were briefly anesthetized with CO_2 _and transferred to an empty 8-dram glass vial. A foam stopper was placed approximately 3 cm down into the vial and approximately 4.5 g of Drierite was placed on top of the stopper. The open end of the vial was then sealed with Parafilm. The flies were checked hourly for mortality, which was characterized by the inability of the flies to resume an upright position after the vial was shaken. Desiccation resistance was expressed as survival time (hours) and was estimated for 50 flies (10 vials, each containing 5 flies) from each treatment group.

### Oxygen consumption and carbon dioxide production assay

Age synchronized cohorts of adult male flies were cultured on plus and minus DOX food and transferred to fresh vials every other day. The rate of CO_2 _emission was used to determine the metabolic rate of the flies and was measured using flow-through respirometry. The CO_2 _emission of 6 groups of flies (14-21 individuals per group) from each treatment was measured once a week for 8 weeks. During the respirometry assays, room air was pumped through three silica gel columns plus one Drierite/Ascarite/Drierite column to remove water vapor and CO_2_. The water and CO_2_-free air then flowed through six respirometer chambers containing the flies, as well as an empty control chamber, and subsequently to the CO_2 _analyzer. Air flow through the respirometry chambers was regulated by a system of computer-controlled valves (Sable Systems, Henderson, NV, USA), which allowed each of the six groups of flies to be measured sequentially. The volume of the respirometry chambers was 12 ml, and the rate of air flow through the chambers was 20 ml/minute. The rate of CO_2 _emission of each fly group was measured for 15 minutes weekly using a Sable Systems Licor LI-6251 infrared CO_2 _analysis system. The room temperature was maintained at 25 ± 1°C. The CO_2 _levels (ppm) were averaged and recorded once/second using Sable Systems data acquisition software. To ensure that the CO_2 _recordings had reached steady-state levels, only data from the last 5-7 minutes of the measurements were used in the data analyses. Oxygen concentrations (Pa) in the outflowing air were measured using an Oxilla (Sable Systems) differential oxygen analyzer. The respiratory quotient of the control and experimental flies was compared at every time interval and not found to be statistically significantly different. We therefore used the CO_2 _measurements to describe the metabolic rate since these measurements are more precise than those of oxygen consumption.

### Aconitase enzyme assay

The effect of MnSOD over-expression on aconitatse activity was examined in age-synchronized cohorts of male progeny from the following lines: Control, *MnSOD(2)12*, and *MnSOD(2)20*. Cohorts were cultured on plus and minus DOX food and passaged to fresh vials every other day. On days 7, 21, 35, 49 and 63, triplicate samples of 5 male flies from each of 6 conditions (3 genotypes +/- DOX) were frozen at -72°C. Aconitase was measured on fresh fly homogenates using a modification of the method of Rose and O'Connell [121] that employs a coupled assay with isocitrate dehydrogenase. Aconitase activity was measured with and without activation. Fly homogenates were prepared on ice by grinding 5 flies in 1.5 ml Eppendorf tubes containing 38 μl 100 mM Tris pH 7.4, 1 mM DTPA and 1 mM MgCl_2_. After centrifugation at 16,000 × g (Sorvall Biofuge Fresco; Kendro, Newtown, CT, USA), supernatants were collected and SOD (1,440 units/μl) was added. To activate aconitase, supernatants were placed in a 96-well plate (Cat.# 3371, Corning Inc., Lowell, MA, USA) and diluted with 9 volumes of freshly prepared ice-cold activation buffer (5.5 mM cysteine, pH 7.4, 600 μM ferrous ammonium sulfate, 94 mM Tris pH 7.4) and held for 1 h on ice. Non-activated samples were diluted with 9 volumes of ice cold 100 mM Tris pH 7.4 and assayed immediately. For the assay, a 30 μl sample (activated or non-activated) was placed in a UV transparent 96-well assay plate (Costar 3635). To start the assay, 200 μl of 37°C assay buffer was added to sample wells using a multichannel pipettor. Final assay conditions were 1 mM NADP, 5 mM MgCl_2_, 2 mM sodium citrate, and in 99 mM Tris pH 7.4 at 37°C containing 260 milli-units of isocitrate dehydrogenase (USB 17798)/ml. Aconitase activity was measured by following the rate of formation of NADPH at 340 nm between 2 and 5 minutes in a Spectramax Plate Reader (Molecular Devices, Sunnyvale, CA, USA). Protein concentrations were determined using the Bradford method [122] with bovine serum albumin as a standard. Unless otherwise noted, all reagents were from Sigma-Aldrich.

### RNA isolation and microarray data analysis

An average of 35 μg RNA was isolated from groups of 30 adult male *Drosophila *using the RNAqueous kit (Ambion), and a portion (3 μg) was fractionated on 1.0% agarose gels to determine purity. Total RNA (10 μg) was used as substrate to generate biotinylated cRNA according to standard Affymetrix protocol (Childrens Hospital, Los Angeles, CA, USA).

DrosGenome1 arrays were used to monitor the expression of 13,500 predicted *Drosophila *transcripts in response to specific MnSOD over-expression under the control of a tetracycline-inducible promoter. In total, 20 gene chips were employed with four replicates for each of five conditions. To control for the effect of a 20% delay in aging caused by MnSOD over-expression, cohorts of MnSOD transgenic flies were sampled at the same chronological age (approximately 50% survival of -DOX flies, day 73) as well as at the same 'physiological age' (approximately 50% survival for both +DOX and -DOX flies, day 83 and day 73, respectively). To control for the effect of DOX, control flies treated with or without DOX were sampled at the same chronological age (approximately 50% survival of -DOX flies, day 78) since DOX does not dramatically delay aging. Thus, the following samples were hybridized to the GeneChips: control (DOX) sampled at 50% survival, control (+DOX) sampled at -DOX 50% survival, *MnSOD(2)22 *(-DOX) sampled at 50% survival, *MnSOD(2)22 *(+DOX) sampled at -DOX 50% survival, and *MnSOD(2)22 *(+DOX) sampled at 50% survival.

Gene expression measures were computed using the robust multichip average [123] in the *affy *package for the R statistical programming language [124]. Linear modeling and empirical Bayes analysis [125] was performed using the R *limma *(Linear Models for Microarray data) package [126] to identify genes significantly differentially expressed in response to MnSOD in treated and untreated flies of the same chronological age and, likewise, in flies of the same 'physiological age' while controlling for the effect of DOX on gene expression. *limma *computes an empirical Bayes adjustment for the *t*-test, and is more robust than the standard two-sample *t*-test comparison. Multiple testing was corrected for by the Benjamini and Hochberg method, which controls the FDR [127]. Using this robust method, genes were found to be significantly differentially expressed by both biological and statistical criteria (± 1.2-fold change, FDR 1% (*p *< 0.01)). Notably, a 1.2-fold change cutoff was selected since relatively small changes have been shown to be important for a variety of biological phenomena, including aging [128]. The microarray data discussed in this study have been deposited in the National Center for Biotechnology Information Gene Expression Omnibus (GEO) [129] and are accessible through GEO Series number GSE7159.

### Identification and enrichment of DNA response elements

MnSOD-regulated genes were examined for the presence of specific DNA response elements in the region 2 Kb upstream of the transcriptional start site and the first intron using a custom program (T Goldman, M Lebo, and M Arbeitman, personal communication). The enrichment for a specific motif in a gene list was determined based on a two-stage selection procedure: one at the gene level, and another at the gene set level. First, the statistical significance of finding a specified motif in a particular gene was computed based on a second order Markov model of the background sequence [130] to determine the probability of finding this motif within the region examined versus a designated number of strings (1,000) that were 'randomly' constructed using Markov model nucleotide probabilities. These probabilities were computed for each motif and each gene within the lists of unique up-regulated (409) or down-regulated (322) genes and in the reference list compoised of genes (12,189) represented on the DrosGenome1 Array. Each motif was considered separately, and only genes for which the significance associated with finding that motif had a *p *value < 0.05 were employed in the subsequent enrichment analysis. At the second stage, the number of genes for which a given motif was found to be significant (*p *value < 0.05) in the set of MnSOD-regulated genes (test set) was then tested for enrichment based on the hypergeometric distribution by comparison to the reference set (genes represented on the DrosGenome1 Array). In particular, MnSOD-regulated genes were queried for the following motifs: the ARE core motif, TGACNNNGC [65,66]; HRE, GGAAGC [64]; DRE, TATCGATA [68,69]; HIF-1 response element (HIF-RE), TCACGTCC [67]; DBE, TTGTTTAC [70]; and DAE, CTTATCA [23].

### Functional annotation and statistical overrepresentation of Gene Ontology classifications

Lists of differentially expressed genes were mapped onto the GO classification [44] to allow for the examination of specific molecular functions, biological processes, and cellular components that were influenced by MnSOD over-expression or other interventions. Comparisons of the distribution of MnSOD and age-dependent changes across the functional categories described by GO allowed for the identification of statistically and biologically relevant patterns of gene expression, as it did for the other comparisons of interest. To this end, GOstat [45,46] was employed to translate lists of differentially expressed genes into functional characterizations of the effect of the condition being examined. Briefly, the number of appearances of each GO term annotated to a gene differentially expressed in a particular condition or group was determined and compared to the number of appearances in a reference list based either on the DrosGenome1 Array or some subset thereof. Statistically overrepresented GO categories were identified by the calculation of a *p *value denoting the probability that the observed numbers of counts could have resulted from randomly distributing a particular GO term between the test and reference group. The *p *value is approximated by a Chi-square distribution (Fisher's Exact test when the expected number of counts is <5), and multiple testing was corrected for by controlling the FDR at a level of 1% (*p *< 0.01).

### Comparison of MnSOD-regulated genes to published data

A more thorough comparison of the results presented here with other published data would require a full treatment of the raw data to ensure a common normalization routine and statistical determination of differential gene expression. In several instances the raw data were not available in microarray repositories, nor upon request, and more rigorous comparisons could not be made. This was especially unfortunate for studies in which the number of replicates was low and primitive statistical procedures were employed to process the data, since methods in microarray data analysis continue to improve considerably. Since the raw data were not available for all the studies of interest, the processed data were utilized for all comparisons. In support of this approach, a recent study aimed at the identification of biomarkers from multiple cancer datasets discussed similar issues in the use of raw versus processed data [131]. The authors concluded that a meta-review approach using processed data was highly concordant with a meta-analysis approach based on re-analysis of the raw data.

### Comparison of gene expression changes in *Drosophila *resulting from MnSOD over-expression to normal aging

Previously, Landis *et al. *[10] examined the transcriptional profile of normal aging in male *Drosophila *using DrosGenome1 arrays and reported the up-regulation of 271 genes and the down-regulation of 656 genes. The gene expression patterns of MnSOD over-expression were compared to those of normal aging by considering genes that were altered in either the same or opposing directions.

### Comparison of gene expression changes resulting from MnSOD over-expression in *Drosophila *to *C. elegans daf-2 *mutants

McElwee and colleagues [74,75] previously described the transcriptional outputs of long-lived dauers and identified genes regulated by *daf-2 *in a *daf-16 *dependent manner. The gene expression patterns of MnSOD over-expressing *Drosophila *were compared to these lists by mapping pairs of *D. melanogaster *and *C. elegans *reciprocal best BLAST hits [31] onto the respective microarrays with allowance for fly genes with multiple close worm homologues. Worm-fly orthologs representing 3,542 unique fly genes and 4,940 total worm-fly pairs resulted. Of the 412 genes up-regulated by MnSOD in flies sampled at both the same chronological and the same 'physiological' age, 169 have a worm ortholog based on the above mapping. Of the 1,160 genes up-regulated in the *daf-2/daf-16 *dataset, 185 have fly orthologs with 25 genes being identified in both studies. If the list is expanded to include all 656 genes (265 have a corresponding worm ortholog) that are up-regulated by MnSOD when flies are sampled at the same chronological age, five additional genes are identified in the overlap. Likewise, if the list includes all 858 (337 have a corresponding worm ortholog) genes up-regulated by MnSOD when flies are sampled at the same 'physiological age', 10 additional genes are identified in the overlap.

### Comparison of gene expression changes in *Drosophila *resulting from MnSOD over-expression to phenobarbital induced xenobiotic stress

Previously, King-Jones *et al. *[76] studied the *Drosophila *xenobiotic response by treating *CanS *flies with PB and examining the resultant transcriptional profiles using Drosophila2 arrays. Comparisons between MnSOD-regulated genes (identified using Affymetrix DrosGenome1 arrays) and xenobiotic regulated genes (identified using Drosophila2 arrays) were made by considering only those probesets that represent 'good matches', according to the manufacturer. This resulted in 8,636 genes being mapped to probes on both arrays. Out of the 656 genes up-regulated at the same chronological age due to MnSOD over-expression, 411 are considered matches between these two arrays based on this criterion. Likewise, of the 503 genes up-regulated by PB treatment [76], 337 are considered matches. From these lists, 59 genes were found to be up-regulated in both conditions.

### Comparison of gene expression changes in *Drosophila *resulting from MnSOD over-expression to altered insulin signaling

Puig *et al. *[108] previously reported the up-regulation of 277 genes in response to insulin stimulation in S2 cells that expressed the constitutively active dFOXO construct, dFoxoA3, using DrosGenome1 arrays. A separate study by Junger *et al. *[107] also examined the transcriptional response of *Drosophila *tissue culture cells to insulin stimulation. In this study, stationary Kc167 cells treated with 100 nM insulin for 2 hours were compared to untreated controls using DrosGenome1 arrays. A selection of candidate genes demonstrating two-fold or greater repression upon insulin stimulation was reported in the published work, and examination of the previously processed data suggests that 481 and 199 genes were up- and down-regulated, respectively, greater than two-fold 2 hours post insulin stimulation.

### Comparison of gene expression changes in *Drosophila *resulting from MnSOD over-expression to yeast re-feeding

Recently, Gershman *et al. *[97] characterized the transcriptional profiles of female *Drosophila *during the first 12 hours of yeast re-feeding after dietary restriction using DrosGenome1 arrays. Using a change point statistic, the authors identified 3,519 differentially expressed genes, of which 2,310 were up-regulated and 1,209 down-regulated. The authors also compared these profiles to those described by Puig *et al. *[108] to identify potential nutrient-mediated dFOXO targets. In this study, MnSOD-regulated genes were compared to those either up- or down-regulated upon yeast re-feeding.

### Statistical significance of overlapping gene sets

The statistical significance of the overlap between various gene sets was evaluated by computing the *p *value representing the probability of obtaining the observed number of overlaps by chance under a hypergeometric distribution. Additionally, the significance of the observed level of overlap between differentially expressed genes for several of the comparisons was assessed by Monte Carlo simulation using custom scripts. In cases where a mapping between orthologs or between Affymetrix probe identifiers was necessary, this step was included in the simulation. For example, for the comparison of genes that were up-regulated by MnSOD over-expression in *Drosophila *to those that were up-regulated in *daf-2 *worms in a *daf-16 *dependent manner, 412 and 1,199 genes were randomly selected from the appropriate total list of genes represented on the DrosGenome1 array and *C. elegans *whole genome Affymetrix array, respectively. These genes were then mapped onto the list of ortholog pairs, and the number of overlaps between these two lists was counted. This procedure was repeated 10,000 times to produce a distribution of overlap results from the random simulations and an approximate *p *value was computed by comparing the actual overlap to this distribution (Additional data file 5). Using the appropriate total gene lists, simulations were also performed for the comparison used to identify aging biomarkers as well as for the comparison of MnSOD-regulated genes and xenobiotic detoxification genes.

## Abbreviations

AMPK, adenosine monophosphate (AMP)-activated protein kinase; ARE, antioxidant response element; CI, confidence interval; DAE, DAF-16 associated element; DBE, DAF-16 binding element; DOX, doxycycline; DRE, DNA replication-related element; EcR, ecdysone receptor; ET, electron transport; FDR, false discovery rate; GO, Gene Ontology; GST, glutathione-S-transferase; HIF, hypoxia induction factor; HIF-RE, hypoxia induction factor-1 (HIF-1) response element; HRE, hydrogen peroxide response element; IIS, insulin/insulin-like growth factor-like signaling; ILP, insulin-like peptide; InR, insulin receptor; JNK, c-Jun-N-terminal kinase; MAPK, mitogen-activated protein kinase; NF-κB, nuclear factor-kappa beta; PB, phenobarbital; ROS, reactive oxygen species; SOD, superoxide dismutase.

## Authors' contributions

JT conceived and designed the study with help from GNL and ST. NH created the transgenic constructs and strains, and GNL, NH, MW, DFord, AL, and AB assayed life span, transgene expression and stress resistance. NBW and RLL designed and carried out aconitase assays, and DFolk and TJB designed and carried out CO_2 _production and dessication-resistance assays. CC, DA, DS, and ST designed and carried out the statistical and bioinformatic analyses. CC designed and carried out cross-dataset comparisons and contributed analysis tools. CC wrote the paper.

## Additional data files

The following additional data are available with the online version of this paper. Additional data file 1 provides additional methods, including statistical analysis of the effect of DOX-induced MnSOD over-expression on lifespan, stress resistance, desiccation, metabolism, and aconitase levels and LacZ expression assay. Additional data file 2 provides additional results for the effect of DOX-induced MnSOD over-expression on lifespan, stress resistance, desiccation, metabolism, and aconitase levels and LacZ expression assay. Additional data file 3 lists DOX regulated immune response genes. Additional data file 4 lists annotated differentially expressed genes resulting from MnSOD over-expression. Additional data file 5 shows the statistical significance of overlapping gene sets. Additional data file 6 is a categorization of the gene expression differences between MnSOD over-expressing flies and controls sampled at the same 'physiological age'. Additional data file 7 shows additional longevity promoting genes conserved between *C. elegans daf-2 *mutants and MnSOD over-expressing *Drosophila*. Additional data file 8 shows MnSOD-regulated xenobiotic detoxification genes. Additional data file 9 gives the DNA regulatory elements in the set of conserved longevity promoting genes. Additional data file 10 provides supporting results and additional explanatory text. Additional data file 11 describes the proposed role for HR96 in the endocrine regulation of lifespan.

## Supplementary Material

Additional data file 1 Statistical analysis of the effect of DOX induced MnSOD over-expression on lifespan, stress resistance, desiccation, metabolism, and aconitase levels and LacZ expression assay. Click here for file

Additional data file 2 Additional results for the effect of DOX-induced MnSOD over-expression on lifespan, stress resistance, desiccation, metabolism, and aconitase levels and LacZ expression assay. Click here for file

Additional data file 3 DOX regulated immune response genes. Click here for file

Additional data file 4 Annotated differentially expressed genes resulting from MnSOD over-expression. Click here for file

Additional data file 5 Statistical significance of overlapping gene sets. Click here for file

Additional data file 6 Categorization of the gene expression differences between MnSOD over-expressing flies and controls sampled at the same 'physiological' age. Click here for file

Additional data file 7 Additional longevity promoting genes conserved between *C. elegans daf-2 *mutants and MnSOD over-expressing *Drosophila*. Click here for file

Additional data file 8 MnSOD-regulated xenobiotic detoxification genes. Click here for file

Additional data file 9 DNA regulatory elements in the set of conserved longevity promoting genes. Click here for file

Additional data file 10 Supporting results and additional explanatory text. Click here for file

Additional data file 11 Proposed role for HR96 in the endocrine regulation of lifespan. Click here for file
