# Erratum to: Transcriptional profiling of MnSOD-mediated lifespan extension in *Drosophila* reveals a species-general network of aging and metabolic genes

**DOI:** 10.1186/s13059-016-0959-3

**Published:** 2016-05-06

**Authors:** Christina Curtis, Gary N Landis, Donna Folk, Nancy B Wehr, Nicholas Hoe, Morris Waskar, Diana Abdueva, Dmitriy Skvortsov, Daniel Ford, Allan Luu, Ananth Badrinath, Rodney L. Levine, Timothy J. Bradley, Simon Tavaré, John Tower

**Affiliations:** Molecular and Computational Biology Program, Department of Biological Sciences, University of Southern California, Los Angeles, CA 90089-1340 USA; Department of Ecology and Evolutionary Biology, University of California, Irvine, CA 92717 USA; Laboratory of Biochemistry, National Heart, Lung, and Blood Institute, Bethesda, MD 201817- 6735 USA; Department of Pathology and Laboratory Medicine, Childrens Hospital Los Angeles, Keck School of Medicine, University of Southern California, Los Angeles, CA 90089-9034 USA; Department of Oncology, University of Cambridge, Cambridge, CB2 2XZ UK

The published version of this article [1] contains a duplicated *Rp49* loading control in the lower panel of Figure 1. The corrected Figure 1 is presented here. Calculations for levels of gene expression in the original article remain unchanged, as they were generated using the correct control panel. The authors apologize for this error.

**Fig. 1:**
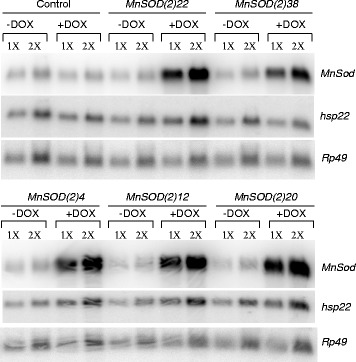
Northern analysis of MnSOD and hsp22 expression in control and transgenic lines. Northern analysis for controls and transgenic lines MnSOD(2)22, MnSOD(2)38, MnSOD(2)4, MnSOD(2)12, and MnSOD(2)20 demonstrates the induction of MnSOD transgene expression by DOX administration and the increased expression of hsp22 due to MnSOD over-expression. Rp49 represents the loading control; 1X = 5 μg RNA, 2X = 10 μg RNA

## References

[1] Curtis C, Landis GN, Folk D, Wehr NB, Hoe N, Waskar M (2007). Transcriptional profiling of MnSOD-mediated lifespan extension in *Drosophila* reveals a species-general network of aging and metabolic genes. Genome Biol.

