# Supplementary material for: Transcriptional profiling of MnSOD-mediated lifespan extension in Drosophila reveals a species-general network of aging and metabolic genes
Source: Genome Biol. 2007 Dec 9;8(12):R262. doi: 10.1186/gb-2007-8-12-r262 (PMC2246264; doi:10.1186/gb-2007-8-12-r262)
Supplement: Additional data file 1 — Statistical analysis of the effect of DOX induced MnSOD over-expression on lifespan, stress resistance, desiccation, metabolism, and aconitase levels and LacZ expression assay. [file gb-2007-8-12-r262-S1.pdf]

Additional methods:

Statistical analysis of the effect of DOX induced MnSOD over-expression on lifespan, stress resistance, desiccation, metabolism, and aconitase levels and LacZ expression assay

The effect of DOX induced MnSOD expression during development and adulthood. The nature of the effect of DOX on lifespan in MnSOD transgenic flies was investigated on age-synchronized cohorts of male progeny for control, *MnSOD(2)12*, and *MnSOD(2)22* flies. The 1146 flies were evenly divided by line and cultured in one of four DOX conditions: No DOX exposure, DOX during development only, DOX during adulthood only, or DOX exposure throughout the entire lifespan.

For each of the above lines, the effect of DOX on mean lifespan for the various treatments was examined using ANOVA and Tukey's Honestly Significant Difference (HSD) multiple comparison with the experiment-wise significance level $\alpha = 0.05$. For the control, DOX did not have a significant impact on mean lifespan, regardless of the time when the flies were exposed, whereas *MnSOD(2)12* and *MnSOD(2)20* exhibited significant differences in mean lifespan due to the timing of DOX treatment (Table S1). For *MnSOD(2)12*, the timing of DOX exposure had a significant effect on lifespan such that flies exposed during adulthood or across the entire lifespan lived significantly longer than flies with no DOX exposure or exposure only during development. Likewise, for *MnSOD2(22)* flies that were exposed to DOX during adulthood or across the entire lifespan lived significantly longer than flies that were exposed to DOX only during

development, or flies that received no DOX exposure. Bar graphs of the results are shown in Additional data file 2, Figure S7 (a).

For each line and treatment the effect of DOX on maximum lifespan was also assayed. Chi-squared (χ^2) analyses were performed, comparing the proportion of flies alive before and after this point (treating maximum lifespan as the dependent variable). For the control, the maximum lifespan was found to be significantly longer for flies exposed to DOX during adulthood only (Pearson χ^2 (1) = 11.40, $p \leq 0.01$), as compared to flies with no DOX exposure. For *MnSOD(2)12*, maximum lifespan was significantly increased by DOX exposure during adulthood only (Pearson χ^2 (1) = 10.61, $p \leq 0.001$), and by DOX exposure across the entire lifespan (Pearson χ^2 (1) = 11.48, $p \leq 0.001$). For *MnSOD(2)22*, maximum lifespan was significantly increased by DOX exposure during adulthood only (Pearson χ^2 (1) = 25.96, $p << 0.001$), and by DOX exposure across the entire lifespan (Pearson χ^2 (1) = 17.21, $p << .0.0001$). Bar graphs of the results are shown in Additional data file 2, Figure S7 (b).

The effect of DOX on mean adult lifespan.

Statistical analyses were conducted for three different cohorts of several MnSOD lines, and the corresponding control. To determine if DOX treatment had an effect on lifespan, ANOVAs were performed twice for each line, once including all flies in the analysis and once excluding flies that died within the first two weeks. Very similar results were obtained with or without censoring the early-dying flies. For the control (cohorts 1, 2, and 3 combined), the effect of the presence of DOX on lifespan was similar regardless of

whether all flies are included or if early-dying flies were excluded (Table S2). *MnSOD(2)12* (cohorts 1, 2, and 3 combined) also showed a significant effect for DOX, with DOX+ flies living significantly longer than DOX- flies. For *MnSOD(2)20* (cohorts 1 and 3 combined), DOX had a significant effect on lifespan such that DOX+ flies lived longer than DOX- flies. For *MnSOD(2)22* (cohorts 1, 2, and 3 combined), DOX also had a significant positive effect on lifespan. In general, when flies that died early were excluded from the analysis, the significance of the DOX effect was more pronounced, although the magnitude of the difference in lifespan remained consistent. A summary of these results is presented in Additional data file 2, Table S2.

The effect of DOX treatment during adulthood on lifespan, stress-resistance, and desiccation.

The effect of DOX treatment during adulthood on mean and maximal lifespan (defined here as the 90th percentile of lifespan) was assessed using log-rank and Chi-squared tests, respectively. The effect of DOX on stress and desiccation resistance was also assayed by measuring survivorship and significant differences between control and treated samples were assessed using the log-rank test (unpaired t-test for desiccation assay). A summary of the results for the effect of DOX on lifespan, stress resistance, and desiccation resistance is presented in Additional data file 2, Table S3.

The effect of DOX treatment on the mean, median, and the 90th percentile of lifespan were further assessed by constructing 95% bootstrap confidence intervals for the ratio of the means and for the ratio of percentiles of the control and treatment populations.

Likewise, bootstrap confidence intervals were constructed for the CO_2 production and heat stress resistance assays. In all cases, B1 = 5,000, B2 = 1,000, and $\alpha = 0.05$. The results are presented in Additional data file 2, Table S4.

The effect of DOX on CO_2 production, O_2 consumption, and Respiratory Quotient. Statistical analyses were carried out to investigate the nature of the effect of DOX on CO_2 production, O_2 consumption, and Respiratory Quotient (RQ = CO_2 production/O_2 consumption) as indicators of metabolic rate, in both MnSOD transgenic flies and the control. Once a week for eight weeks, CO_2 production was measured for six samples of flies from the control, *MnSOD(2)12*, *MnSOD(2)20*, and *MnSOD(2)22* lines in both treatment conditions (DOX+ or DOX-), for a total of 96 measures per line. The values presented represent the average CO_2 production per fly. O_2 consumption measures were also taken at each time for the MnSOD lines. The effect on respiratory quotient (RQ) was also examined. For the control, only three samples were measured each week for a total of 48 measures per line. The data were analyzed by ANOVA with DOX treatment as the independent variable and either CO_2 production (nl CO_2/ minute), O_2 consumption (nl O_2/ minute), or RQ (CO_2 production/O_2 consumption) as the dependent variable, averaged across the weeks and results are shown in Additional data file 2, Table S5 and S6.

For control A, CO_2 production did not differ significantly for DOX+ and DOX- flies, indicating similar metabolic rates. For *MnSOD(2)12* A, DOX significantly impacted CO_2 production such that DOX- flies produced more CO_2 than DOX+ flies. For *MnSOD(2)20*

A, DOX- flies showed higher CO_2 production than DOX+ flies. As with the other transgenic lines, *MnSOD(22)* A DOX- flies also produced significantly more CO_2 than DOX+ flies. Similarly, for lines *MnSOD(2)*12 B, *MnSOD(2)20* B, and *MnSOD(22)* B, DOX had a significant impact on CO_2 production such that DOX- flies produced more CO_2 than DOX+ flies. Note that there was no control B in this assay. For the O_2 consumption assays, a statistically significant difference in O_2 consumption between DOX+ and DOX- control flies was not detected. *MnSOD(2)12* flies consumed significantly more oxygen in the absence of DOX than in the presence of DOX. Although the difference only approached significance, *MnSOD(2)20* DOX- flies also consumed more oxygen that DOX+ flies. DOX treatment had no effect on oxygen consumption for *MnSOD(2)22*. For all lines tested, a statistically significant difference in RQ between DOX+ and DOX- flies was not detected. The impact of DOX on CO_2 production, O_2 consumption, and RQ is summarized in Additional data file 2, Table S6.

The effect of DOX on RQ across lifespan. In order to assess whether RQ changes during lifespan, regression analyses were performed for each line and treatment with RQ as the outcome variable and age (by week) as the predictor variable. The results indicate that for the untreated (DOX-) control and *MnSOD(2)22*, RQ decreased over time. For all treated (DOX+) lines, RQ remained stable over time (Additional data file 2, Figure S7).

The effect of DOX on aconitase levels. To determine whether aconitase levels were altered by DOX treatment, ANOVAs were performed for each of the lines treating

aconitase levels (mU/mg) as the dependent variable. In all analyses, the effect of DOX was not found to be statistically significant (Additional data file 2, Table S8).

LacZ expression assay. In order to test the possibility that MnSOD over-expression reduces DOX uptake or the effective concentration in transgenic flies, the expression of lacZ driven by a DOX-inducible promoter was quantified in flies coincident with the over-expression of MnSOD, using two independent MnSOD transgenic lines (Additional data file 2, Figure S8). To control for possible rtTA dosage effects, unrelated genotypes with varying numbers of target constructs were also assayed. Assay of lacZ activity in fly extracts was performed essentially as previously described [1]. Young adult male flies of the indicated genotypes were cultured on +DOX and –DOX vials for two weeks. Three flies were homogenized in an eppendorf tube using a fitted pestle, in 100 μl of ice-cold homogenization buffer (50 mM KPO_4). Then 400 μl of additional homogenization buffer was added to each sample, the extracts were centrifuged for 5 minutes to spin down the debris, and the supernatants were retained. Each extraction was done in triplicate. The Bradford assay (Bio-Rad) was used to measure protein concentration of each extract. 5 μl of each fly extract was added to a well in a 96 well plate. Then 250 μl of 1X Bradford reagent was added, and the assay was allowed to incubate at room temperature for ten minutes prior to measuring absorbance at 595 nm. BSA standards were used to generate a standard curve. To assay beta-galactosidase activity, fly extract was added to the wells of a 96 well plate at 5 μl per sample, and 200 μl of CPRG (chlorophenol red-beta-D-galactopyranoside) reagent (Sigma) dissolved in H_2O was added. The plate was incubated at 37°C, and absorbance was then measured at 574 nm at 0, 30, 45, and 60

minute time points. The amount of extract protein used was adjusted so that the reaction was linear with regard to amount of extract and time of reaction. Beta-galactosidase activity was measured as the difference between the 60 minute and 30 minute time points divided by the protein concentration of each extract to generate specific activity in relative units. Data are presented as the average +/- standard deviation of the triplicate extracts. The lacZ reporter strain w^{1118}*; p{7T40-lacZ}E1* is as previously described [2]. The "Ubb" transgenic strains will be described in detail elsewhere (N. Hoe and J.Tower, submitted). Strain Ubb^{wt118D} contains the wild-type *Drosophila ubiquitin* cDNA cloned downstream of the doxycycline-regulated promoter, with two inserts on the second chromosome. Strains $Ubb^{+1\text{-}1D}$ and $Ubb^{+1\text{-}11D}$ contain a 3' deleted *ubiquitin* cDNA that does not encode a full-length protein, and each strain contains two inserts on the second chromosome. The control genotype, *LacZ/+;+/+,* was generated by crossing strain w^{1118}*; p{7T40-lacZ}E1* to w^{1118}. The experimental genotypes contain the *rtTA(3)E2* driver insertion on the third chromosome. Statistical significance was determined using unpaired, two-sided t-tests. LacZ activity was found to vary somewhat across genotypes, and the expression of LacZ in the presence of MnSOD over-expression was within the range observed with the various controls. These data indicate that MnSOD over-expression does not significantly alter the uptake or effective concentration of DOX relative to controls.

As an additional means of assessing the uptake of DOX in the MnSOD transgenic lines as compared to controls, we assayed the effective concentration of DOX in fly extracts based upon the ability of the extracts to inhibit the growth of *E.coli*, a DOX-sensitive

bacteria, in liquid culture. However, we found that DOX-fed flies did not detectably inhibit bacterial growth, so DOX uptake in flies could not be determined by such a direct assay (data not shown).

References

1. Simon JA, Lis JT: **A germline transformation analysis reveals flexibility in the organization of heat shock consensus elements**. *Nucleic Acids Res* 1987, **15**:2971-2988.
2. Bieschke ET, Wheeler JC, Tower J: **Doxycycline-induced transgene expression during Drosophila development and aging**. *Mol Gen Genet* 1998, **258**(6):571-579.
