# Supplementary material for: Transcriptional profiling of MnSOD-mediated lifespan extension in Drosophila reveals a species-general network of aging and metabolic genes
Source: Genome Biol. 2007 Dec 9;8(12):R262. doi: 10.1186/gb-2007-8-12-r262 (PMC2246264; doi:10.1186/gb-2007-8-12-r262)
Supplement: Additional data file 2 — Additional results for the effect of DOX-induced MnSOD over-expression on lifespan, stress resistance, desiccation, metabolism, and aconitase levels and LacZ expression assay. [file gb-2007-8-12-r262-S2.pdf]

Additional results for the effect of DOX induced MnSOD over-expression on lifespan, stress resistance, desiccation, metabolism, and aconitase levels and LacZ expression assay

(a)

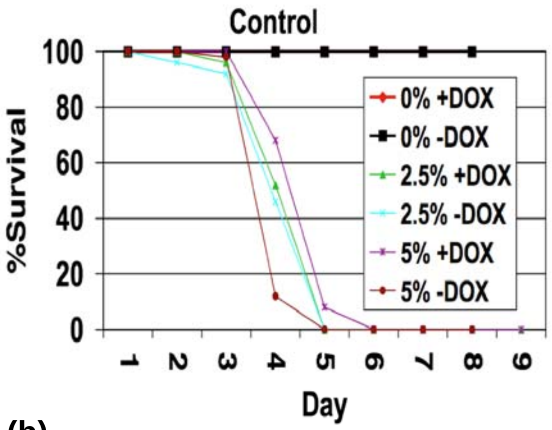

(b)

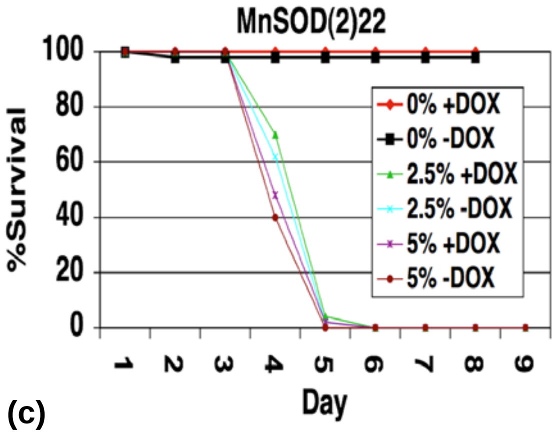

(c)

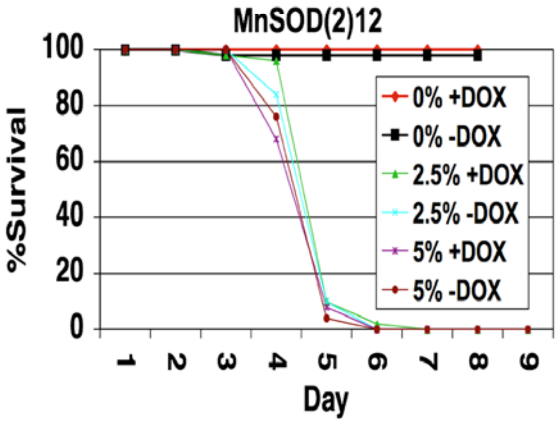

Figure S1: Hydrogen peroxide survival. MnSOD hydrogen peroxide survival. The percentage of animals alive is plotted against animal age for (a) control, (b) *MnSOD(2)22*, and (c) *MnSOD(2)12*.

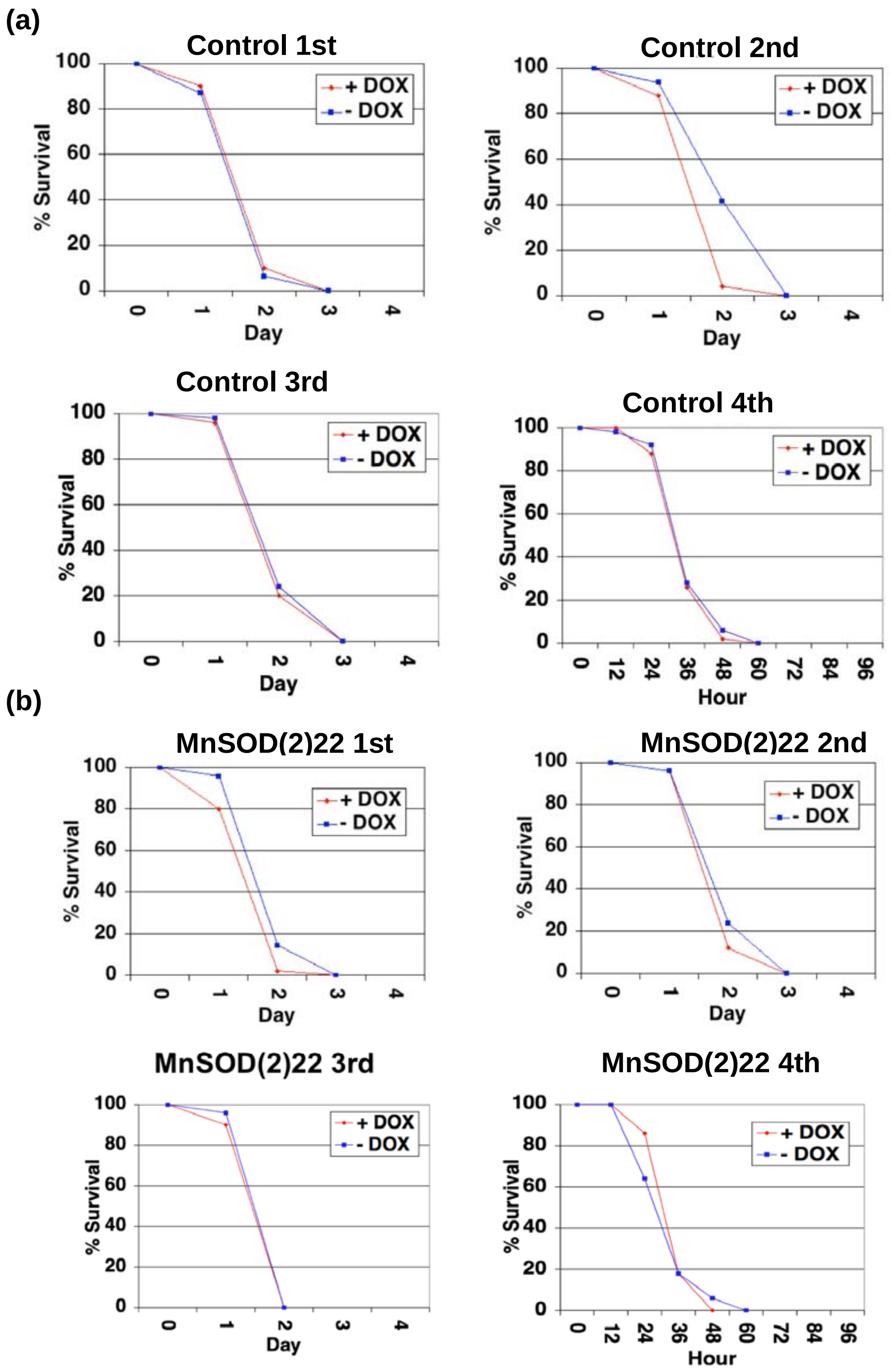

Figure S2: 20 mM Paraquat survival. The percentage of animals alive is plotted against animal age for (a) control, (b) *MnSOD(2)22*, (c) *MnSOD(2)20*, and (d) *MnSOD(2)12*; 1st- 4th indicates the cohort.

(c)

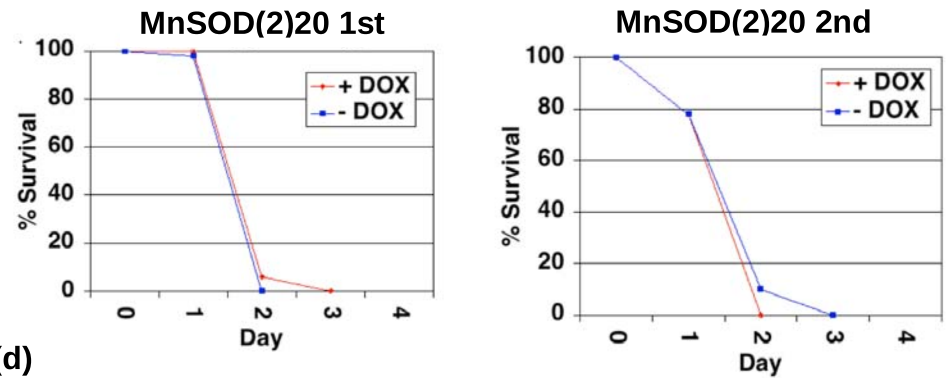

(d)

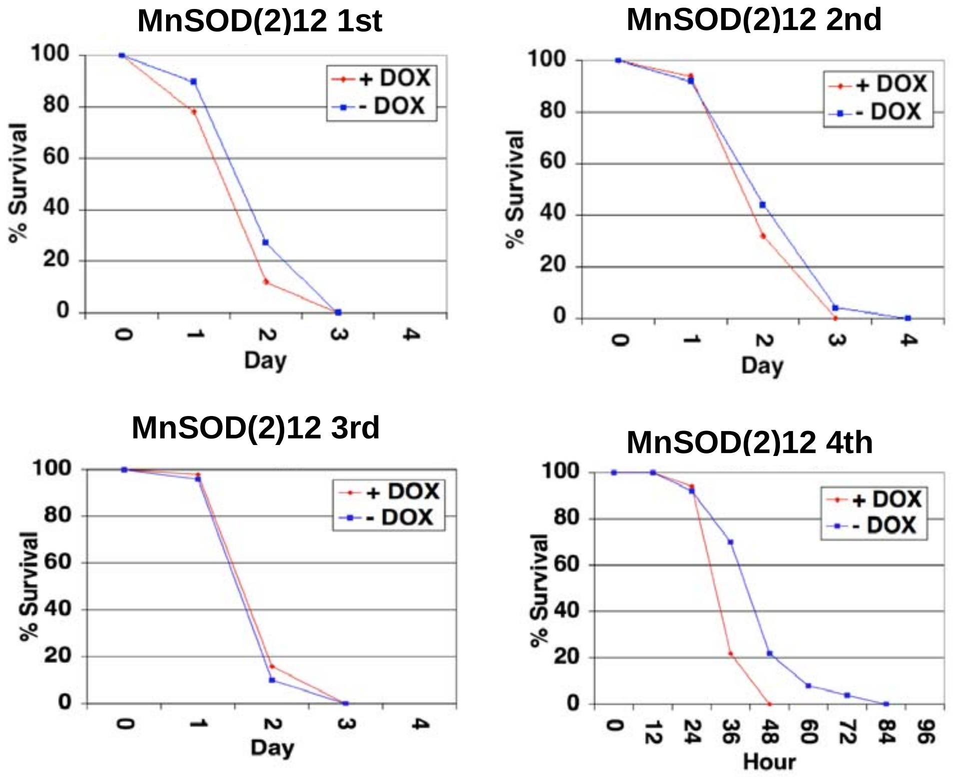

Figure S2, Continued: 20 mM Paraquat survival. The percentage of animals alive is plotted against animal age for (a) control, (b) *MnSOD(2)22*, (c) *MnSOD(2)20*, and (d) *MnSOD(2)12*; 1st- 4th indicates the cohort.

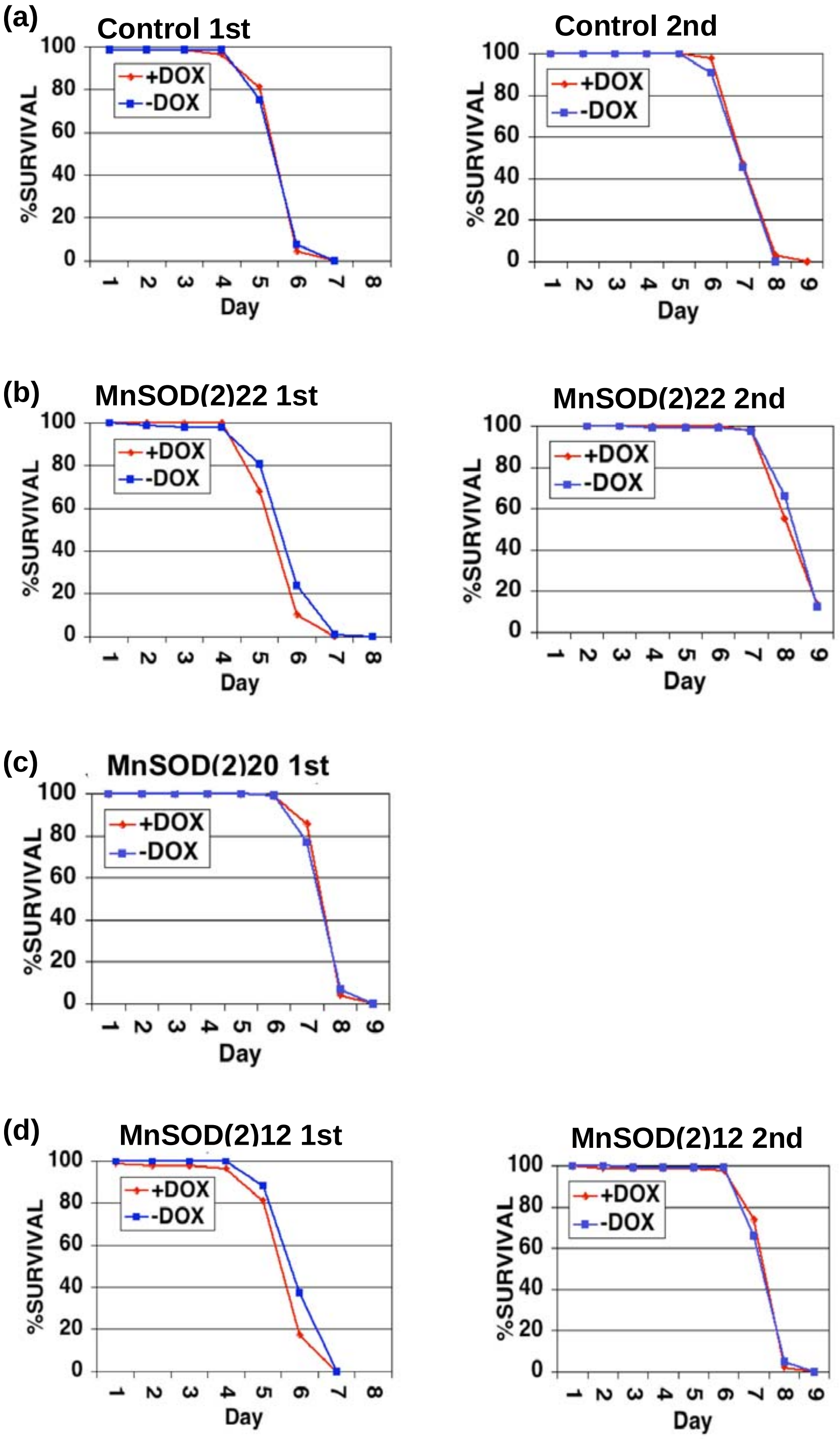

Figure S3: 100% Oxygen survival. The percentage of animals alive is plotted against animal age for (a) control, (b) *MnSOD(2)22*, (c) *MnSOD(2)20*, and (d) *MnSOD(2)12*.

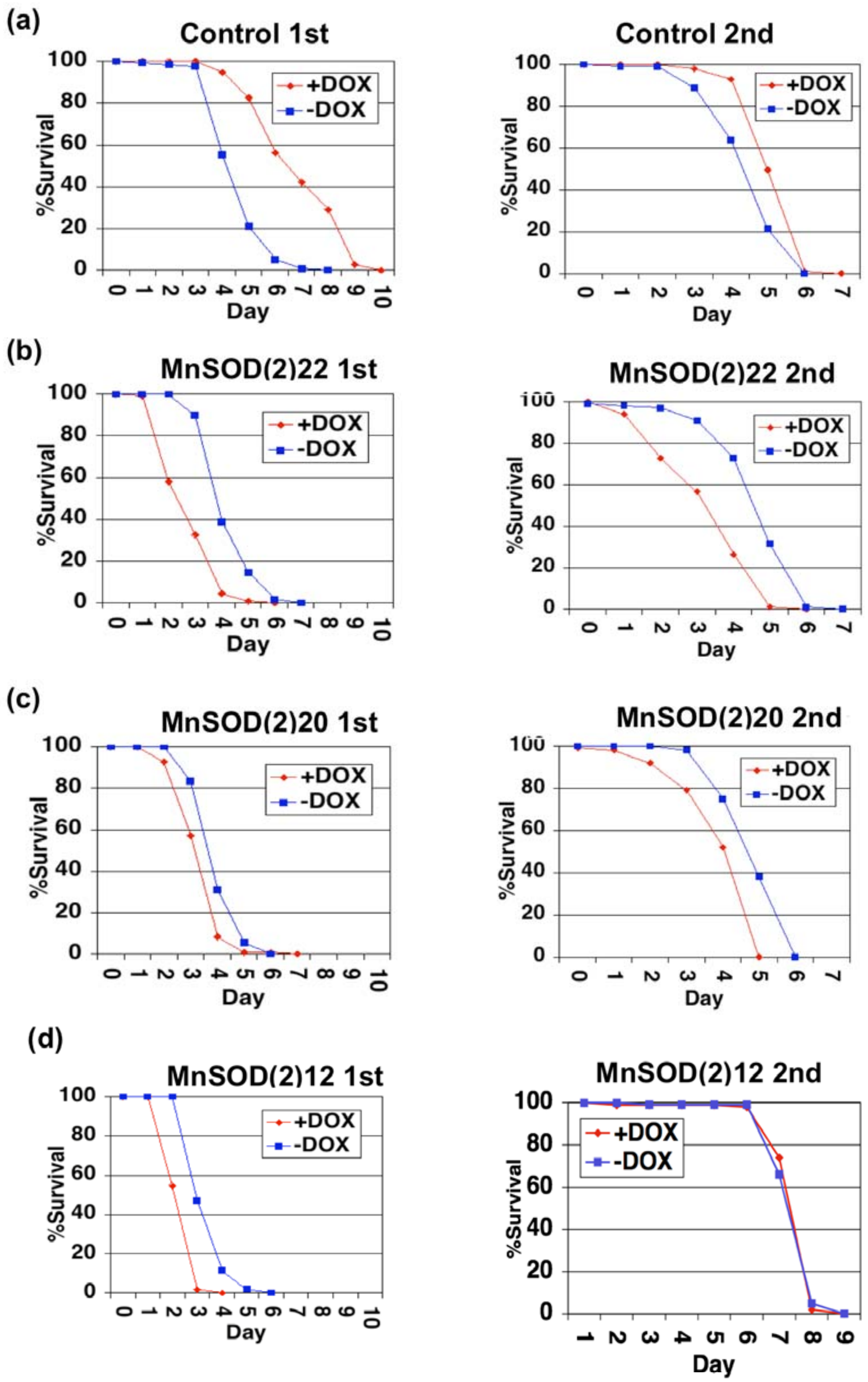

Figure S4: 34 °C survival. The percentage of animals alive is plotted against animal age for lines (a) control, (b) *MnSOD(2)22*, and (c) *MnSOD(2)20*, and (d) *MnSOD(2)12*.

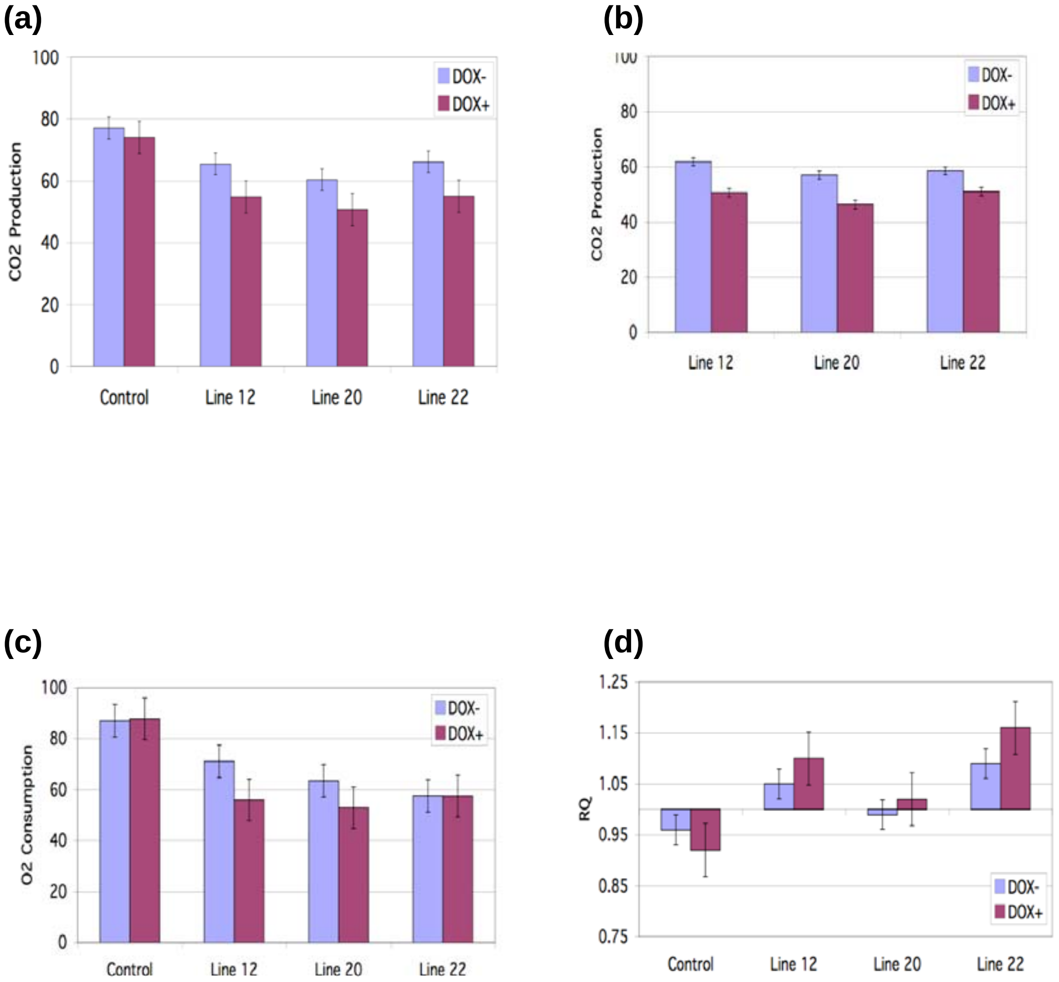

Figure S5: CO_2 production, O_2 consumption, and Respiratory quotient. The average (a) CO_2 production (nl CO_2/ minute) for Group A, (b) CO_2 production (nl CO_2/ minute) for Group B, (c) O_2 consumption (nl O_2/ minute), and (d) RQ (CO_2 production/O_2 consumption) are shown for the control, *MnSOD(2)12* (Line 12), *MnSOD(2)20* (Line 20), and *MnSOD(2)22* (Line 22).

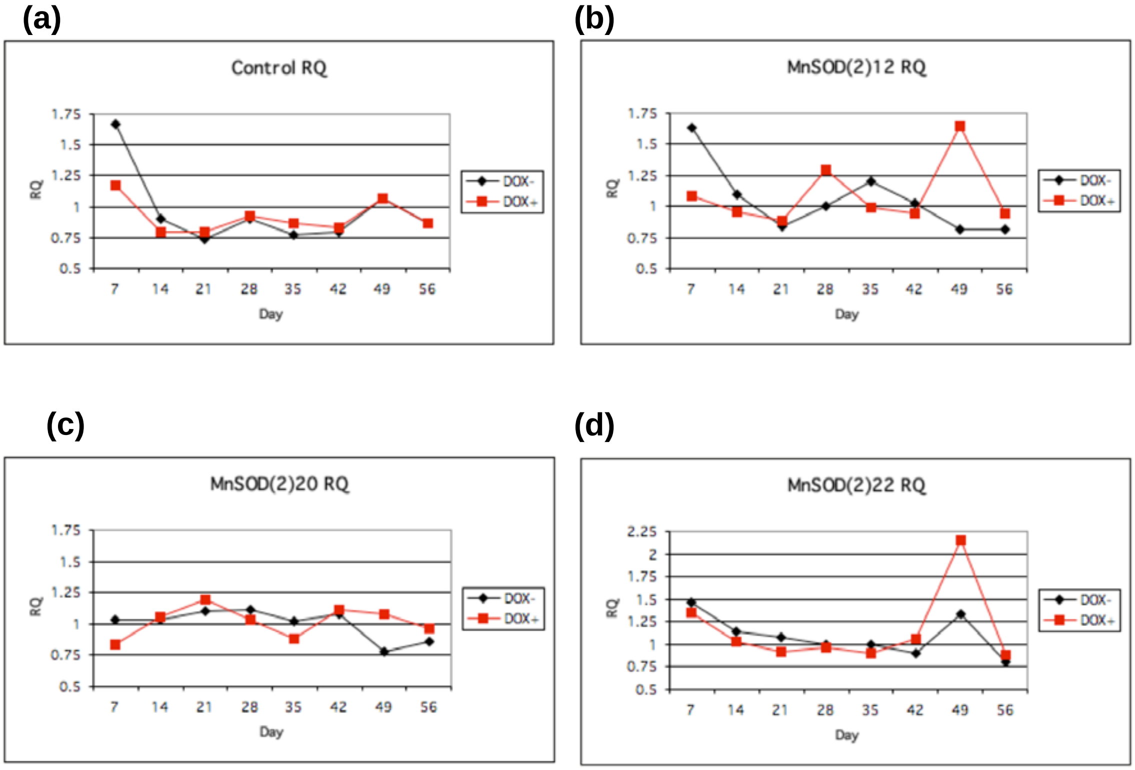

Figure S6: Respiratory quotient over lifespan. The RQ is plotted across lifespan (by week) for the (a) control, (b) *MnSOD(2)12*, (c) *MnSOD(2)20*, and (d) *MnSOD(2)22*.

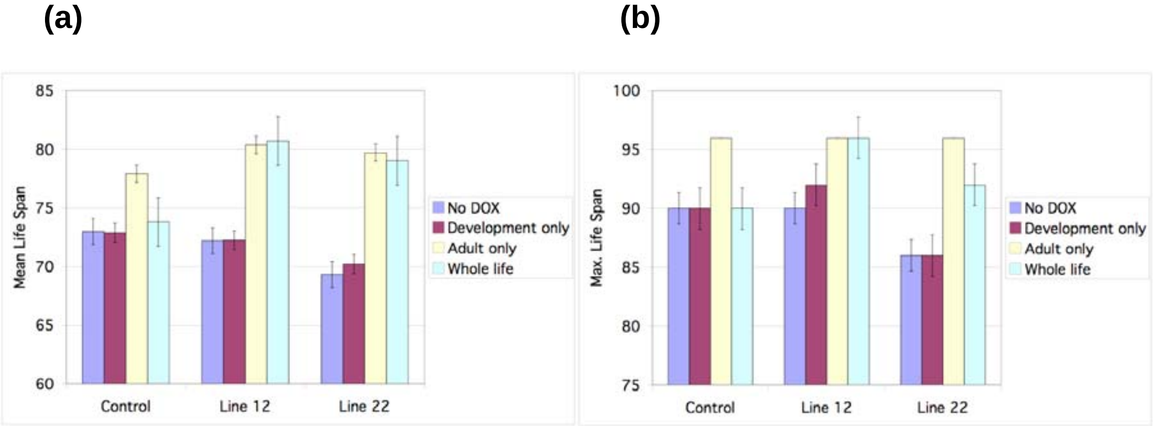

Figure S7: Effect of DOX on lifespan during development and adulthood. The effect of DOX treatment during development only, during adulthood only, throughout life or, no treatment (control) on (a) mean lifespan and (b) maximum lifespan is shown for the control, *MnSOD(2)12* (Line 12), and *MnSOD(2)22* (Line 22).

(a) ANOVA table

Genotype	Effect		DF	Sum Sq	Mean Sq	F value	P-value
Control	Timing of DOX	Between	3	1647.05	549.02	2.42	0.07
		Within	380	86377.57	549.02		
		Total	383	88024.63			
MnSOD(2)12	Timing of DOX	Between	3	6673.63	2224.54	10.11	< 1.00 x 10-3
		Within	382	84082.98	220.11		
		Total	385	90756.61			
MnSOD(2)22	Timing of DOX	Between	3	8745.57	2915.19	14.43	< 1.00 x 10-3
		Within	372	75152.43	202.02		
		Total	375	83898.00			

(b) Tukey HSD

			Subset 1	Subset 2
**Genotype**	**DOX Treatment**	N	**Mean**	
MnSOD(2)12	No DOX	89	72.20	
	Development only	102	72.25	
	Adult only	99		80.38
	Whole life	96		80.71
	Sig.		1	0.999
MnSOD(2)22	No DOX	95	69.33	
	Development only	96	70.21	
	Adult only	91		79.72
	Whole life	94		79.03
	Sig.		0.974	0.987

(c) Descriptive statistics

Genotype	DOX Treatment	N	Mean	STD	SEM
Control	No DOX	98	73.00	13.42	1.36
	Development only	97	72.89	15.53	1.58
	Adult only	97	77.92	16.71	1.70
	Whole life	92	77.80	14.44	1.51
	Total	384	73.41	15.16	0.77
MnSOD(2)12	No DOX	89	72.20	15.39	1.63
	Development only	102	72.25	15.90	1.57
	Adult only	99	80.38	15.90	1.39
	Whole life	96	80.71	14.08	1.44
	Total	386	76.43	14.08	0.78
MnSOD(2)22	No DOX	95	69.33	13.88	1.42
	Development only	96	70.21	14.08	1.44
	Adult only	91	79.72	15.02	1.55
	Whole life	94	79.03	13.84	1.45
	Total	376	74.50	14.96	0.77

Table S1: Effect of timing of DOX treatment on mean lifespan. (a) ANOVA table. The results of the effect of DOX on mean lifespan for the various treatments based on ANOVA. (b) Tukey's HSD. Significant differences in group means were identified using Tukey's Honestly Significant Difference (HSD) multiple comparison for $\alpha = 0.05$. Means that differed significantly from one another are listed in separate columns. (c) Descriptive statistics. The mean lifespan for each of the lines and DOX treatments, sample size, standard deviation (STD), and standard error of the mean (SEM) are listed.

(a) ANOVA table

Sample	Genotype	Effect		DF	Sum Sq	Mean Sq	F value	P-value
**All flies**	Control	DOX	Between	1	6762.10	6762.10	18.67	< 1.00 x 10-3
	Cohorts 1,2,3		Within	1172	424556.79	362.25		
			Total	1173	431318.88			
**Excluding ealy deaths**	Control	DOX	Between	1	4135.56	4135.56	17.65	< 1.00 x 10-3
	Cohorts 1,2,3		Within	1118	262024.21	234.37		
			Total	1119	266159.77			
**All flies**	*MnSOD(2)12*	DOX	Between	1	32815.70	32815.70	96.47	< 1.00 x 10-3
	Cohorts 1,2,3		Within	1147	390173.38	340.17		
			Total	1148	422989.08			
**Excluding ealy deaths**	*MnSOD(2)12*	DOX	Between	1	32320.19	32320.19	130.72	< 1.00 x 10-3
	Cohorts 1,2,3		Within	1114	275441.38	247.25		
			Total	1115	307761.58			
**All flies**	*MnSOD(2)20*	DOX	Between	1	28405.88	28405.88	85.19	< 1.00 x 10-3
	Cohorts 1,2,3		Within	775	258408.80	333.43		
			Total	776	286814.68			
**Excluding ealy deaths**	*MnSOD(2)20*	DOX	Between	1	25578.09	25578.09	111.76	< 1.00 x 10-3
	Cohorts 1,2,3		Within	752	172107.52	228.87		
			Total	753	197685.61			
**All flies**	*MnSOD(2)22*	DOX	Between	1	41287.68	41287.68	197.87	< 1.00 x 10-3
	Cohorts 1,2,3		Within	1099	229324.08	208.67		
			Total	1100	270611.76			
**Excluding ealy deaths**	*MnSOD(2)22*	DOX	Between	1	36502.86	36502.86	99.48	< 1.00 x 10-3
	Cohorts 1,2,3		Within	1155	423822.95	366.95		
			Total	1156	460325.81			

Table S2: Summary of the effect of DOX on mean adult lifespan. (a) ANOVA table. ANOVA results for the effect of DOX on mean lifespan in adult flies for various lines (where cohorts 1, 2, and 3 have been combined) are presented. Two ANOVAs were performed for each line: One including all flies, the other excluding early dying flies (those that died prior to day 14). (b) Summary of results for the effect of DOX on mean lifespan. The mean lifespan for each of the lines in the presence or absence of DOX are presented for the case when all flies are included in the analysis or when early dying flies are excluded. Also listed is the difference in lifespan between DOX+ and DOX- treated flies (δ), the F value from the ANOVA, and the significance of the effect (Sig.). *Note that line 20 was tested in cohorts 1 and 3 only.

	All flies					Excluding early-dying flies				
	Lifespan (days)					Lifespan (days)				
**Line**	**DOX-**	**DOX+**	**δ**	**F value**	**Sig.**	**DOX-**	**DOX+**	**δ**	**F value**	**Sig**
**MnSOD Coh. 1**										
Control	51.53	58.27	6.74	10.91	0.001	58.17	61.22	3.06	3.99	0.05
MnSOD(2)4	61.20	68.24	7.04	18.10	< 0.001	62.50	71.17	8.61	47.13	< 0.001
MnSOD(2)12	58.17	68.63	10.46	26.26	< 0.001	61.47	70.55	9.08	27.74	< 0.001
MnSOD(2)20	58.51	71.68	13.17	50.62	< 0.001	60.9	73.41	12.50	65.35	< 0.001
MnSOD(2)22	50.72	62.87	12.15	32.70	< 0.001	54.25	69.81	15.56	115.65	< 0.001
MnSOD(2)38	61.13	67.38	6.25	8.09	0.01	66.41	71.64	5.22	11.59	< 0.001
**MnSOD Coh. 2**										
Control	63.41	65.74	2.33	2.10	0.15	64.52	66.73	2.21	2.42	0.12
MnSOD(2)4	62.72	69.09	6.37	11.53	0.001	64.82	71.33	6.51	18.78	< 0.001
MnSOD(2)12	63.94	70.21	6.27	9.55	0.002	65.44	73.57	8.13	24.51	< 0.001
MnSOD(2)22	62.42	73.01	10.59	35.25	< 0.001	64.34	74.83	10.49	53.79	< 0.001
**MnSOD Coh. 3**										
Control	64.94	70.29	5.35	8.28	0.004	65.83	72.52	6.69	16.85	< 0.001
MnSOD(2)12	57.74	72.98	15.24	101.95	< 0.001	58.02	72.98	14.96	101.20	< 0.001
MnSOD(2)20	63.05	73.95	10.90	35.06	< 0.001	64.70	75.43	10.73	47.42	< 0.001
MnSOD(2)22	58.46	69.19	10.73	36.87	< 0.001	59.93	70.58	10.65	48.75	< 0.001
**Cohorts 1 & 2**										
Control	57.46	62.01	4.55	11.44	0.001	61.53	64.04	2.51	5.562	0.02
MnSOD(2)4	61.95	68.66	6.71	28.90	< 0.001	63.67	71.25	7.58	60.30	< 0.001
MnSOD(2)12	61.06	69.43	8.37	33.49	< 0.001	63.50	72.06	8.56	51.36	< 0.001
MnSOD(2)22	56.48	67.95	11.47	63.29	< 0.001	59.34	72.44	13.10	153.21	< 0.001
**Cohorts 1, 2, & 3**										
Control	60.00	64.80	4.80	18.67	< 0.001	63.05	66.89	3.84	17.65	<0.001
MnSOD(2)12	59.95	70.64	10.69	96.47	< 0.001	61.62	2.38	10.76	130.72	<0.001
*MnSOD(2)20**	60.73	72.82	12.09	85.19	< 0.001	62.78	74.43	11.65	111.76	<0.001
MnSOD(2)22	57.14	68.37	11.23	99.48	< 0.001	59.54	71.78	12.24	197.87	<0.001

Table S2, Continued: Summary of the effect of DOX on mean adult lifespan. (a) ANOVA table. ANOVA results for the effect of DOX on mean lifespan in adult flies for various lines (where cohorts 1, 2, and 3 have been combined) are presented. Two ANOVAs were performed for each line: One including all flies, the other excluding early dying flies (those that died prior to day 14). (b) Summary of results for the effect of DOX on mean lifespan. The mean lifespan for each of the lines in the presence or absence of DOX are presented for the case when all flies are included in the analysis or when early dying flies are excluded. Also listed is the difference in lifespan between DOX+ and DOX- treated flies (δ), the F value from the ANOVA, and the significance of the effect (Sig.). *Note that line 20 was tested in cohorts 1 and 3 only.

		Mean lifespan[a]				Maximum lifespan[b]			
**Cohort**	**Line**	**-DOX**	**+DOX**	**%Δ**	**p-value**	**-DOX**	**+DOX**	**%Δ**	**p-value**
**Lifespan**									
Cohort 1	Control	51.52±21.06	58.27±19.12	13.08%	2.60 x 10-5	72	78	8%	0.01
Cohort 2	Control	63.55±16.36	65.86±15.40	3.61%	0.50	82	80	-2%	0.20
Cohort 3	Control	64.94±17.62	70.29±19.43	8.25%	3.15 x 10-5	82	88	7%	0.48
Cohort 1, 2, & 3	Control	60.00±19.35	64.80±18.71	8%	1.01 x 10-6	82	82	0%	0.09
Cohort 1	*MnSOD(2)22*	50.72±17.50	62.87±23.86	23.95%	0.00	70	84	20%	0.00
Cohort 2	*MnSOD(2)22*	62.62±18.03	73.01±16.92	16.59%	1.50 x 10-11	82	88	7%	0.00
Cohort 3	*MnSOD(2)22*	58.46±15.96	69.19±18.80	18.36%	2.97 x 10-13	78	88	13%	0.00
Cohort 1, 2, & 3	*MnSOD(2)22*	57.14±17.78	68.37±20.44	20%	0.00	78	88	13%	0.00
Cohort 1	*MnSOD(2)20*	58.50±18.38	71.68±18.22	22.51%	2.33 x 10-15	78	88	13%	0.00
Cohort 3	*MnSOD(2)20*	63.05±18.49	73.95±17.69	17.29%	8.80 x 10-11	82	90	10%	0.00
Cohort 1 & 3	*MnSOD(2)20*	60.73±18.55	72.82±17.97	20%	0.00	82	90	10%	0.00
Cohort 1	*MnSOD(2)12*	58.16±19.55	68.63±20.14	17.99%	2.96 x 10-11	80	90	13%	0.00
Cohort 2	*MnSOD(2)12*	63.22±18.93	70.21±20.36	11.06%	2.85 x 10-5	88	88	0%	0.32
Cohort 3	*MnSOD(2)12*	57.74±16.27	72.98±13.31	26.39%	1.11 x 10-16	82	88	7%	0.00
Cohort 1, 2, & 3	*MnSOD(2)12*	59.95±18.64	70.64±18.24	18%	0.00	82	88	7%	0.00
Cohort 1	*MnSOD(2)4*	61.20±15.30	68.24±17.33	11.50%	1.13 x 10-8	78	84	8%	0.00
Cohort 2	*MnSOD(2)4*	62.72±17.94	69.26±18.60	10.43%	7.21 x 10-6	82	88	7%	0.00
Cohort 1 & 2	*MnSOD(2)4*	61.95±16.65	68.74±17.95	11%	2.45 x 10-12	82	86	5%	0.00
Cohort 1	*MnSOD(2)38*	61.13±21.03	67.38±21.86	10.22%	1.44 x 10-8	78	86	10%	0.00
**Survival - 2.5% H2O2**									
Cohort 1	Control	3.34±0.745	3.48±0.580	4.19%	0.46				
Cohort 1	*MnSOD(2)22*	3.64±0.525	3.74±0.527	2.75%	0.34				
Cohort 1	*MnSOD(2)12*	3.94±0.511	4.06±0.511	3.05%	0.30				
**Survival - 5% H2O2**									
Cohort 1	Control	3.10±0.364	3.76±0.591	21.29%	9.57 x 10-9				
Cohort 1	*MnSOD(2)22*	3.40±0.495	3.50±0.544	2.94%	0.34				
Cohort 1	*MnSOD(2)12*	3.80±0.495	3.76±0.591	-1.05%	0.74				
**Survival - 20 mM Paraquat**									
Cohort 1	Control	1.94±0.438	2.00±0.452	3.09%	0.47				
Cohort 2	Control	2.35±0.601	1.92±0.396	-18.30%	3.89 x 10-5				
Cohort 3	Control	2.97±0.162	2.95±0.221	-0.67%	0.54				
Cohort 4	Control	1.98±0.377	1.90±0.364	-4.04%	0.17				
Cohort 1, 2, 3, &	Control	2.39±0.697	2.25±0.672	-5.86%	0.05				
Cohort 1	*MnSOD(2)22*	2.10±0.425	1.82±0.438	-13.33%	1.83 x 10-3				
Cohort 2	*MnSOD(2)22*	2.20±0.495	2.08±0.396	-5.45%	0.17				
Cohort 3	*MnSOD(2)22*	2.96±0.200	2.90±0.303	-2.03%	0.25				
Cohort 4	*MnSOD(2)22*	1.70±0.580	1.86±0.351	9.41%	0.11				
Cohort 1, 2, 3, &	*MnSOD(2)22*	2.24±0.638	2.17±0.574	-3.13%	0.15				
Cohort 1	*MnSOD(2)20*	1.98±0.141	2.06±0.240	4.04%	0.05				
Cohort 2	*MnSOD(2)20*	1.88±0.558	1.78±0.418	-5.32%	0.20				
Cohort 1 & 2	*MnSOD(2)20*	1.93±0.408	1.92±0.367	-0.52%	0.84				
Cohort 1	*MnSOD(2)12*	2.17±0.595	1.90±0.580	-12.44%	0.03				
Cohort 2	*MnSOD(2)12*	2.40±0.700	2.26±0.565	-5.83%	0.20				
Cohort 3	*MnSOD(2)12*	2.96±0.211	2.98±0.154	0.67%	0.32				
Cohort 4	*MnSOD(2)12*	2.18±0.629	1.90±0.240	-12.84%	0.01				
Cohort 1, 2, 3, &	*MnSOD(2)12*	2.45±0.688	2.31±0.683	-5.71%	0.04				

[a] Mean lifespan, days +/- SD, *p* value for log-rank test.

[b] Maximum lifespan, days, *p* value for Chi^2 test

%Δ = (+DOX)-(-DOX)/(-DOX)

Table S3: Summary of the effect of DOX on lifespan, stress, and desiccation resistance. The results of log-rank and Chi-squared tests to assess the effect of DOX treatment during adulthood on mean and maximal lifespan, respectively are reported. The effect of DOX on stress and desiccation resistance as measured by mean survivorship was also assessed. The mean +/- the standard deviation, percent change in treated versus untreated samples, and *p*-values are reported.

Cohort	Line	Mean lifespan[a] -DOX	+DOX	%Δ	p-value
**Survival - 100% Oxygen**					
Cohort 1	Control	5.84±0.544	5.84±0.517	0.00%	0.94
Cohort 2	Control	6.36±0.646	6.48±0.598	1.89%	0.31
Cohort 1 & 2	Control	7.37±0.653	7.14±0.784	-3.12%	0.50
Cohort 1	*MnSOD(2)22*	6.00±0.814	5.78±0.616	-3.66%	3.78 x 10-3
Cohort 2	*MnSOD(2)22*	6.73±0.819	6.67±0.735	-0.89%	0.38
Cohort 1 & 2	*MnSOD(2)22*	7.34±0.901	7.22±0.808	-1.64%	0.03
Cohort 2	*MnSOD(2)20*	6.83±0.554	6.89±0.451	0.88%	0.41
Cohort 1	*MnSOD(2)12*	6.25±0.655	5.95±0.748	-4.80%	4.01 x 10-3
Cohort 2	*MnSOD(2)12*	6.67±0.725	6.70±0.769	0.45%	0.55
Cohort 1 & 2	*MnSOD(2)12*	7.47±0.721	7.34±0.844	-1.74%	0.15
**Survival - 34°C**					
Cohort 1	Control	4.77±1.02	7.07±1.63	48.16%	0.00
Cohort 2	Control	4.72±0.980	5.41±0.700	14.77%	1.22 x 10-7
Cohort 1 & 2	Control	4.75±1.00	6.30±1.53	24.60%	0.00
Cohort 1	*MnSOD(2)22*	4.45±0.911	2.95±0.985	-33.69%	0.00
Cohort 2	*MnSOD(2)22*	4.95±1.019	3.51±0.1.273	-29.18%	5.33 x 10-15
Cohort 1 & 2	*MnSOD(2)22*	4.67±1.04	3.22±1.16	-32.98%	0.00
Cohort 1	*MnSOD(2)20*	4.20±0.782	3.60±0.811	-14.25%	5.13 x 10-7
Cohort 2	*MnSOD(2)20*	5.11±0.827	4.24±0.970	-16.98%	4.36 x 10-11
Cohort 1 & 2	*MnSOD(2)20*	4.64±0.923	3.89±0.982	-16.16%	1.01 x 10-13
Cohort 1	*MnSOD(2)12*	3.65±0.742	2.56±0.534	-28.87%	0.00
Cohort 2	*MnSOD(2)12*	4.58±0.945	3.46±1.058	-24.45%	7.23 x 10-13
Cohort 1 & 2	*MnSOD(2)12*	4.08±0.977	2.98±0.961	-26.96%	0.00
**Survival - Desiccation[c]**					
Cohort 1	Control	3.1±1.4	3.4±0.6	9.70%	0.66
Cohort 2	Control	5.5±2.2	4.7±1.0	-14.50%	0.34
Cohort 1	*MnSOD(2)22*	4.2±1.5	3.3±1.0	-21.40%	< 0.05
Cohort 2	*MnSOD(2)22*	4.1±1.8	4.5±1.7	9.80%	0.46
Cohort 1	*MnSOD(2)20*	2.4±0.6	2.5±0.4	4.20%	0.60
Cohort 2	*MnSOD(2)20*	4.8±1.8	4.7±1.3	-2.10%	0.98
Cohort 1	*MnSOD(2)12*	3.1±0.3	2.6±0.4	-16.10%	<0.05
Cohort 2	*MnSOD(2)12*	4.7±0.9	5.0±2.0	6.40%	0.49

[a] Mean lifespan, days +/- SD, *p* value for log-rank test.
[c] Mean lifespan, hours in empty vial with desiccant at 25°C +/- SD, *p* value for unpaired, two-sided t-test.
%Δ = (+DOX)-(-DOX)/(-DOX)

Table S3, Continued: Summary of the effect of DOX on lifespan, stress, and desiccation resistance. The results of log-rank and Chi-squared tests to assess the effect of DOX treatment during adulthood on mean and maximal lifespan, respectively are reported. The effect of DOX on stress and desiccation resistance as measured by mean survivorship was also assessed. The mean +/- the standard deviation, percent change in treated versus untreated samples, and *p*-values are reported.

(a) Lifespan assay

Cohort	Line	û	STD	95% Bootstrap CI	%Δ Mean	%Δ Median	%Δ Maximum
Cohort 1, 2, & 3	Control			Bootstrap t intervals	4.88 – 11.21		
				Double bootstrap intervals	4.81 – 11.25	3.00 – 9.63	0.00 – 0.00
				Basic bootstrap intervals	4.76 – 10.47	2.82 – 9.38	0.00 – 0.00
		8.00	0.0192	Normal approximation	4.20 – 11.74		
Cohort 1, 2, & 3	*MnSOD(2)22*			Bootstrap t intervals	12.78 – 19.72		
				Double bootstrap intervals	12.77 – 19.76	16.18–24.05	5.57 – 12.66
				Basic bootstrap intervals	12.67 – 19.57	16.09–23.66	4.72 – 13.01
		16.18	0.0212	Normal approximation	12.03 – 20.34		
Cohort 1 & 3	*MnSOD(2)20*			Bootstrap t intervals	16.08 – 23.91		
				Double bootstrap intervals	16.17 – 23.84	21.00 – 29.87	9.76 – 13.66
				Basic bootstrap intervals	15.22 – 24.61	21.61 – 29.73	9.76 – 12.20
				Normal approximation	16.08 – 23.71		
		19.91	0.0240				
Cohort 1, 2, & 3	*MnSOD(2)12*			Bootstrap t intervals	15.10 – 21.74		
				Double bootstrap intervals	15.15 – 21.69	18.90 – 27.99	5.11 – 9.49
				Basic bootstrap intervals	15.03 – 21.63	19.08 – 27.31	4.87 – 9.87
		18.35	0.0198	Normal approximation	14.46 – 22.23		
Cohort 1 & 2	*MnSOD(2)4*			Bootstrap t intervals	7.50 – 14.50		
				Double bootstrap intervals	7.64 – 14.35	8.94 – 17.95	-0.20– 4.87
				Basic bootstrap intervals	7.42 – 14.41	8.87 – 18.94	-0.25– 4.87
		10.96	0.0211	Normal approximation	6.83– 15.08		
Cohort 1				Bootstrap t intervals	4.11 – 16.44		
	MnSOD(2)38			Double bootstrap intervals	4.09 – 16.48	-1.94 – 5.88	7.15 – 13.27
				Basic bootstrap intervals	3.68 – 16.07	-3.40 – 5.88	7.69 – 13.01
		10.22	0.0377	Normal approximation	2.83 – 17.61		

Table S4: Bootstrap confidence intervals for lifespan, heat stress, and CO_2 production assays. The effect of DOX treatment on (a) the mean, median, and 90th percentile of lifespan, (b) heat stress resistance, and (c) mean CO_2 production was assessed by constructing 95% bootstrap confidence intervals for the ratio of the means and ratio of percentiles of the control and treatment populations. Where applicable, the bootstrap t-intervals and the normal approximation are reported in addition to the basic bootstrap and double bootstrap confidence intervals. In all cases, the number of bootstrap replicates, B1 = 5,000, B2 = 1,000, and the significance level $\alpha = 0.05$.

(b) Heat stress assay

Cohort	Line	û	STD	95% Bootstrap CI	%Δ Mean	%Δ Median	%Δ Maximum
Cohort 1	Control			Bootstrap t intervals	41.16 – 55.49		
				Double bootstrap intervals	41.07 – 55.53	11.63 – 60.49	50.00 –50.00
				Basic bootstrap intervals	40.85 – 55.06	5.00 – 60.00	50.00 –50.00
		48.16	0.0437	Normal approximation	39.59 – 56.73		
Cohort 2	Control			Bootstrap t intervals	10.27 – 19.59		
				Double bootstrap intervals	10.00 – 11.02	-37.62 – 0.00	NaN
				Basic bootstrap intervals	9.95 – 19.23	-20.00 – 0.00	0.00 – 0.00
		14.77	0.0282	Normal approximation	9.24 – 20.31		
**Cohort 1 & 2**	Control			Bootstrap t intervals	28.09 – 37.76		
				Double bootstrap intervals	27.99 – 37.82	20.00 – 20.00	50.00 – 50.00
				Basic bootstrap intervals	27.82 – 37.51	20.00 – 20.00	50.00 – 50.00
		32.74	0.0292	Normal approximation	27.01 – 38.47		
Cohort 1	*MnSOD(2)22*			Bootstrap t intervals	-37.83 – -29.41		
				Double bootstrap intervals	-37.88 – -29.37	-25.00 – -25.00	-40.57 – -33.33
				Basic bootstrap intervals	-37.97 – -29.73	-25.00 – -10.00	-46.67 – -33.33
		-33.69	0.0250	Normal approximation	-38.58– -28.80		
Cohort 2	*MnSOD(2)22*			Bootstrap t intervals	-33.49– -23.36		
				Double bootstrap intervals	-33.60– -23.19	-20.00 – -7.08	-16.67 – -16.67
				Basic bootstrap intervals	-33.67– -23.58	-20.00 – 0.00	-16.67 – -16.67
				Normal approximation	-34.52– -22.39		
		-28.56	0.0309				
**Cohort 1 & 2**	*MnSOD(2)22*			Bootstrap t intervals	-34.33– -27.54		
				Double bootstrap intervals	-34.38– -27.47	-50.03– -40.00	-17.00 – -17.00
				Basic bootstrap intervals	-34.45– -27.64	-55.00– -40.00	-17.00 – -17.00
		-31.04	0.0203	Normal approximation	-35.03– -27.05		

Table S4, continued: Bootstrap confidence intervals for lifespan, heat stress, and CO_2 production assays. The effect of DOX treatment on (a) the mean, median, and 90th percentile of lifespan, (b) heat stress resistance, and (c) mean CO_2 production was assessed by constructing 95% bootstrap confidence intervals for the ratio of the means and ratio of percentiles of the control and treatment populations. Where applicable, the bootstrap t-intervals and the normal approximation are reported in addition to the basic bootstrap and double bootstrap confidence intervals. In all cases, the number of bootstrap replicates, B1 = 5,000, B2 = 1,000, and the significance level $\alpha = 0.05$.

(b) Heat stress assay

Cohort	Line	û	STD	95% Bootstrap CI	%Δ Mean	%Δ Median	%Δ Maximum
Cohort 1	*MnSOD(2)20*			Bootstrap t intervals	-18.33– -10.22		
				Double bootstrap intervals	-18.34– -10.21	0.00– 13.17	-43.75– -20.00
				Basic bootstrap intervals	-18.49– -10.45	0.00– 25.00	-40.00– -20.00
		-14.26	0.0242	Normal approximation	-19.01– -9.51		
Cohort 2	*MnSOD(2)20*			Bootstrap t intervals	-21.90– -13.88		
				Double bootstrap intervals	-21.96– -13.83	0.00– 55.00	-16.67 – -16.67
				Basic bootstrap intervals	-21.75– -13.77	0.00– 20.00	-16.67 – -16.67
		-17.81	0.0245	Normal approximation	-22.62– -13.00		
**Cohort 1 & 2**	*MnSOD(2)20*			Bootstrap t intervals	-19.23– -13.06		
				Double bootstrap intervals	-19.37– -12.90	-44.01– -20.00	-16.67 – -16.67
				Basic bootstrap intervals	-19.25– -13.12	-40.00– -20.00	-16.67 – -16.67
		-16.15	0.0187	Normal approximation	-19.82– -12.48		
Cohort 1	*MnSOD(2)12*			Bootstrap t intervals	-32.20– -25.50		
				Double bootstrap intervals	-32.38– -25.30	0.00– 49.79	-64.74 – -40.00
				Basic bootstrap intervals	-32.21– -25.62	0.00– 33.33	-55.00 – -40.00
		-28.87	0.0204	Normal approximation	-32.88– -24.86		
Cohort 2	*MnSOD(2)12*			Bootstrap t intervals	-30.07– -20.41		
				Double bootstrap intervals	-30.01– -20.42	-62.64– -40.00	-27.60– -16.67
				Basic bootstrap intervals	-30.05– -20.56	-60.00– -40.00	-33.34– -16.67
		-25.21	0.0287	Normal approximation	-30.83– -19.58		
**Cohort 1 & 2**	*MnSOD(2)12*			Bootstrap t intervals	-30.28– -23.52		
				Double bootstrap intervals	-30.39– -23.46	NaN	-42.01– -2.94
				Basic bootstrap intervals	-30.38– -23.67	-25.00– -25.00	-40.00– -6.67
		-27.00	0.0205	Normal approximation	-31.02– -22.98		

Table S4, continued: Bootstrap confidence intervals for lifespan, heat stress, and CO_2 production assays. The effect of DOX treatment on (a) the mean, median, and 90th percentile of lifespan, (b) heat stress resistance, and (c) mean CO_2 production was assessed by constructing 95% bootstrap confidence intervals for the ratio of the means and ratio of percentiles of the control and treatment populations. Where applicable, the bootstrap t-intervals and the normal approximation are reported in addition to the basic bootstrap and double bootstrap confidence intervals. In all cases, the number of bootstrap replicates, B1 = 5,000, B2 = 1,000, and the significance level $\alpha = 0.05$.

(c) CO_2 production averaged over lifespan

Line	û	STD	95% Bootstrap CI	%Δ Mean
Control			Bootstrap t intervals	NaN
			Double bootstrap intervals	NaN
			Basic bootstrap intervals	NaN
	0.37	0.0013	Normal approximation	NaN
MnSOD(2)22			Bootstrap t intervals	-20.62 – -10.62
			Double bootstrap intervals	-20.83 – -10.31
			Basic bootstrap intervals	-20.50 – -13.10
	-16.69	0.0251	Normal approximation	-21.63 – -11.77
MnSOD(2)20			Bootstrap t intervals	-23.22 – - 8.72
			Double bootstrap intervals	-22.89 – - 9.10
			Basic bootstrap intervals	-21.87 – - 9.97
	-15.81	0.0398	Normal approximation	-23.60 – - 8.02
MnSOD(2)12			Bootstrap t intervals	-20.90 – -10.90
			Double bootstrap intervals	-20.12 – -10.78
			Basic bootstrap intervals	-20.45 – -12.22
	-16.26	0.0276	Normal approximation	-21.69 – -10.85

Table S4, continued: Bootstrap confidence intervals for lifespan, heat stress, and CO_2 production assays. The effect of DOX treatment on (a) the mean, median, and 90th percentile of lifespan, (b) heat stress resistance, and (c) mean CO_2 production was assessed by constructing 95% bootstrap confidence intervals for the ratio of the means and ratio of percentiles of the control and treatment populations. Where applicable, the bootstrap t-intervals and the normal approximation are reported in addition to the basic bootstrap and double bootstrap confidence intervals. In all cases, the number of bootstrap replicates, B1 = 5,000, B2 = 1,000, and the significance level $\alpha = 0.05$.

(a) CO2 production

Genotype	Effect		DF	Sum Sq	Mean Sq	F value	P-value
Control A	DOX	Between	1	212.45	212.45	1.13	0.29
		Within	94	17674.59	188.03		
		Total	95	17887.04			
MnSOD(2)12 A	DOX	Between	1	2727.68	2727.68	31.18	$< 1.00 \times 10^{-3}$
		Within	94	8224.45	87.49		
		Total	95	10952.13			
MnSOD(2)20 A	DOX	Between	1	2177.45	2177.45	20.23	$< 1.00 \times 10^{-3}$
		Within	94	10115.51	107.61		
		Total	95	12292.96			
MnSOD(2)22 A	DOX	Between	1	2935.89	2935.89	28.04	$< 1.00 \times 10^{-3}$
		Within	94	9843.71	104.72		
		Total	95	12779.59			
MnSOD(2)12 B	DOX	Between	1	3039.75	3039.75	25.65	$< 1.00 \times 10^{-3}$
		Within	94	11140.70	118.52		
		Total	95	14180.45			
MnSOD(2)20 B	DOX	Between	1	2718.95	2718.95	27.19	$< 1.00 \times 10^{-3}$
		Within	94	9401.26	100.01		
		Total	95	12120.20			
MnSOD(2)22 B	DOX	Between	1	1328.34	1328.34	10.61	$< 1.00 \times 10^{-3}$
		Within	94	11769.60	125.21		
		Total	95	13097.94			

(b) O2 consumption

Genotype	Effect		DF	Sum Sq	Mean Sq	F value	P-value
Control B	DOX	Between	1	6.02	6.02	0.01	0.93
		Within	46	31871.79	692.87		
		Total	47	31877.81			
MnSOD(2)12 B	DOX	Between	1	5490.38	5490.38	5.45	0.02
		Within	94	94630.96	1006.71		
		Total	95	100121.33			
MnSOD(2)20 B	DOX	Between	1	2709.38	2709.38	2.96	0.09
		Within	94	86046.46	915.39		
		Total	95	88755.83			
MnSOD(2)22 B	DOX	Between	1	0.38	0.38	0.00	0.98
		Within	94	64504.96	686.22		
		Total	95	64505.33			

(c) RQ

Genotype	Effect		DF	Sum Sq	Mean Sq	F value	P-value
Control B	DOX	Between	1	0.03	0.03	0.38	0.54
		Within	46	3.07	0.07		
		Total	47	3.10			
MnSOD(2)12 B	DOX	Between	1	0.04	0.04	0.11	0.74
		Within	94	36.04	0.38		
		Total	95	36.08			
MnSOD(2)20 B	DOX	Between	1	0.01	0.01	0.05	0.82
		Within	94	11.92	0.13		
		Total	95	11.93			
MnSOD(2)22 B	DOX	Between	1	0.11	0.11	0.24	0.62
		Within	94	42.86	0.46		
		Total	95	42.97			

Table S5: Effect of DOX on CO2 production, O2 consumption, and RQ. ANOVA tables for each line for each of the dependent variables (a) CO_2 production, (b) O_2 consumption, (c) RQ.

	Control	*MnSOD(2)12*	*MnSOD(2)20*	*MnSOD(2)22*
**CO2 A**				
DOX-	77.1	65.5	60.4	66.2
DOX+	74.1	54.9	50.8	55.1
% Δ	-3.9%	-16.3%	-15.8%	-16.7%
Sig.	0.29	< 0.001	< 0.001	< 0.001
**CO2 B**				
DOX-	NA	61.9	57.1	58.6
DOX+	NA	50.7	46.4	51.1
% Δ		-18.2%	-18.7%	-12.7%
Sig.		< 0.001	< 0.001	< 0.001
**O2 B**				
DOX-	87.1	71.1	63.5	57.6
DOX+	87.8	56.0	52.9	57.5
% Δ	0.8%	-21.3%	-16.7%	-0.2%
Sig.	0.93	0.02	0.09	0.98
**RQ B**				
DOX-	0.96	1.05	0.99	1.09
DOX+	0.92	1.10	1.02	1.16
% Δ	-4.8%	4.0%	3.4%	6.2%
Sig.	0.54	0.74	0.65	0.62

Table S6: Summary of results for CO_2, O_2, and RQ analyses. For each line tested, the mean CO_2 production, O_2 consumption, or respiratory quotient (RQ) is given for untreated (DOX-) and treated (DOX+) flies. The percent change in each of these measures between DOX treated and untreated flies for each line is listed along with the significance of the result.

	N	Adjusted R^2	Standardized β	Sig.
**Control**				
DOX-	24	0.13	-0.40	0.05
DOX+	24	-0.03	-0.12	0.57
MnSOD(2)12				
DOX-	48	0.04	-0.25	0.09
DOX+	48	-0.001	0.12	0.43
MnSOD(2)20				
DOX-	49	0.02	-0.20	0.18
DOX+	47	-0.02	0.047	0.75
MnSOD(2)22				
DOX-	48	0.10	-0.35	0.02
DOX+	48	-0.02	0.08	0.58

Table S7: Statistical analysis of RQ across lifespan. Results of regression analyses for each line and treatment with RQ as the outcome variable and age (by week) as the predictor variable. Adjusted R^2 refers to the fraction of variance in the outcome variable that is accounted for by the predictor. Standardized β refers the strength and direction of the relationship between the predictor and the outcome and Sig. refers to the statistical significance of the predictor.

(a) ANOVA table

Sample	Genotype	Effect		DF	Sum Sq	Mean Sq	F value	P-value
**Unactivated**	Control	DOX	Between	1	16396.38	16396.38	2.65	0.11
			Within	60	370835.42	6180.59		
			Total	61	387231.80			
**Activated**	Control	DOX	Between	1	7685.16	7685.16	1.04	0.31
			Within	60	443690.08	7394.84		
			Total	61	451375.25			
**Unactivated**	*MnSOD(2)12*	DOX	Between	1	14189.78	14189.78	2.25	0.14
			Within	58	366470.68	6318.46		
			Total	59	380660.46			
**Activated**	*MnSOD(2)12*	DOX	Between	1	21068.56	21068.56	2.41	0.13
			Within	58	508138.07	8761.00		
			Total	59	529206.63			
**Unactivated**	*MnSOD(2)22*	DOX	Between	1	9763.99	9763.99	1.62	0.21
			Within	53	320514.57	6047.45		
			Total	54	330278.56			
**Activated**	*MnSOD(2)22*	DOX	Between	1	3247.87	3247.87	0.39	0.54
			Within	54	455897.67	8442.55		
			Total	55	459145.54			

(b) Descriptive statistics

Genotype	Aconitase	DOX	N	Mean	STD	SEM
Control	Unactivated	-	30	310.06	83.86	15.31
		+	32	277.52	73.38	12.97
		Total	62	293.27	79.68	10.12
	Activated	-	30	383.51	98.41	17.97
		+	32	361.23	72.48	12.81
		Total	62	372.01	86.02	10.93
MnSOD(2)12	Unactivated	-	32	318.58	86.66	15.32
		+	28	349.40	70.35	13.30
		Total	60	332.96	80.32	10.37
	Activated	-	32	406.63	98.25	17.37
		+	28	444.19	87.96	16.62
		Total	60	424.16	94.71	12.23
MnSOD(2)22	Unactivated	-	30	319.51	74.08	13.53
		+	25	346.27	81.99	16.40
		Total	55	331.68	78.21	10.55
	Activated	-	30	406.99	92.032	16.80
		+	36	391.72	91.71	17.99
		Total	56	399.90	91.37	12.21

Table S8: Effect of DOX on aconitase levels. (a) ANOVA tables. For each line tested, aconitase levels were measured in the presence of either activated or unactivated enzyme. The results of ANOVAs treating aconitase levels as the dependent variable are presented. (b) Summary statistics. The mean aconitase levels for each line are given for untreated (DOX-) and treated (DOX+) flies in the presence of either the unactivated or activated enzyme. The percent change in each of these measures between DOX treated and untreated flies is listed along with the significance of the result.

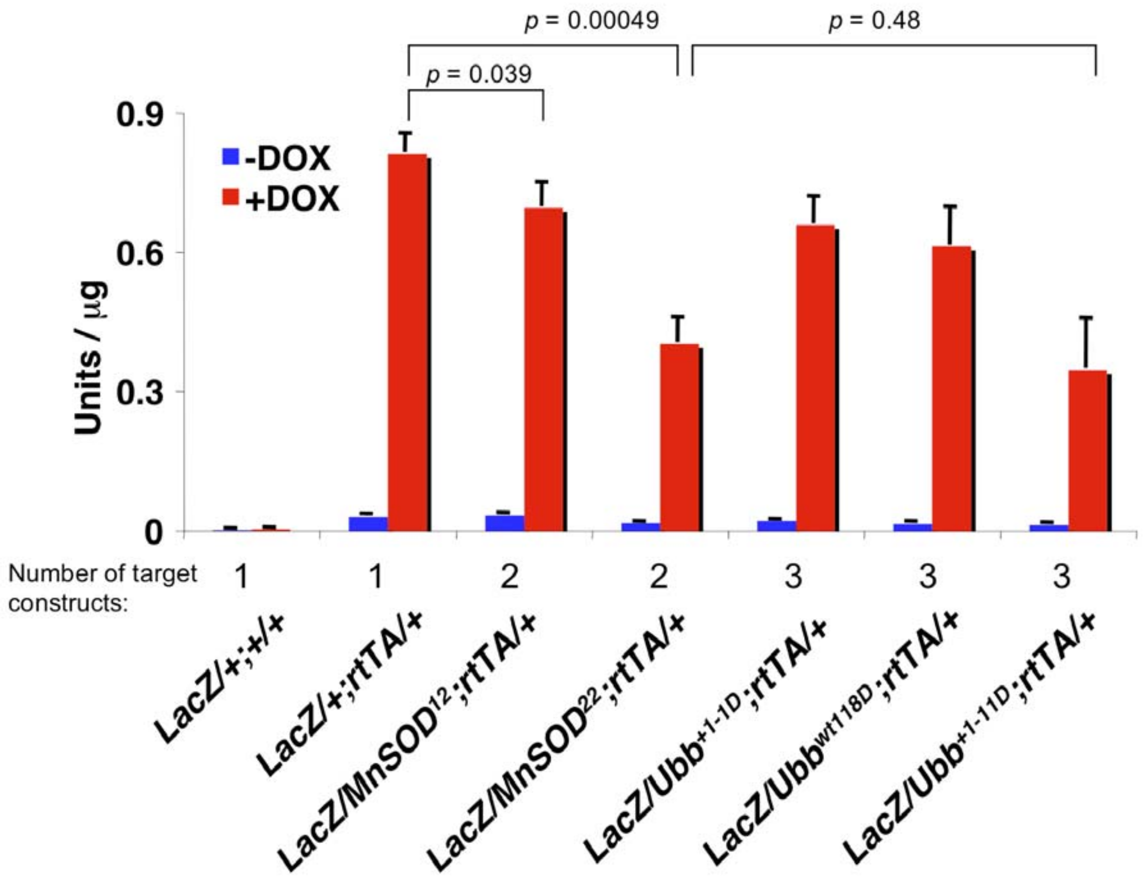

Figure S8: LacZ expression assay. Beta-galactosidase activity in terms of specific activity per μg of protein for each extract is shown for various transgenic lines cultured with or without DOX. Data are presented as the average +/- standard deviation of the triplicate extracts. The number of doxycycline-regulated promoter constructs present in each genotype are as indicated. Statistical significance was determined using unpaired, two-sided t-tests and results for various comparisons are indicated above the graph.
