# Supplementary material for: Transcriptional profiling of MnSOD-mediated lifespan extension in Drosophila reveals a species-general network of aging and metabolic genes
Source: Genome Biol. 2007 Dec 9;8(12):R262. doi: 10.1186/gb-2007-8-12-r262 (PMC2246264; doi:10.1186/gb-2007-8-12-r262)
Supplement: Additional data file 5 — Statistical significance of overlapping gene sets. [file gb-2007-8-12-r262-S5.pdf]

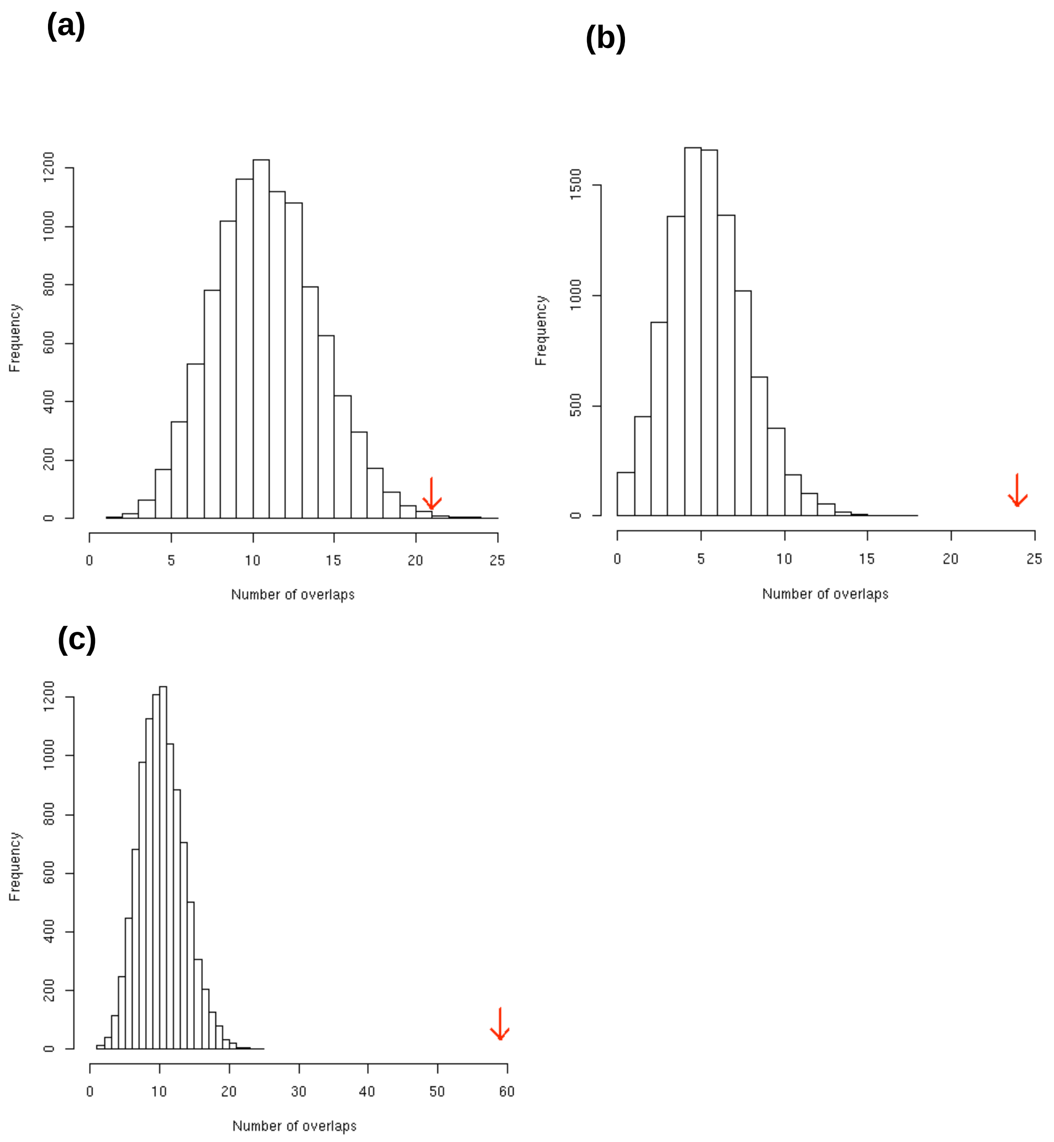

Additional data file 5: Statistical significance of overlapping gene sets. Histograms based on Monte Carlo simulations showing the likelihood of randomly finding overlapping gene sets larger than those identified for: (a) Aging biomarkers, (b) Conserved longevity promoting genes, and (c) MnSOD regulated xenobiotic detoxification genes. For each gene set, 10,000 simulations were performed and the histogram shows the number of genes in the overlap from each trial. The arrows indicate the number of trials that resulted in overlaps that exceeded the observed number of overlaps for each gene set. Approximate *p*-values computed by comparing the observed overlap to the simulated distribution were as follows: Aging biomakers, $p < 0.003$; Conserved longevity promoting genes, $p << 1.00 \times 10^{-4}$; Xenobiotic detoxification genes, $p << 1.00 \times 10^{-4}$.
