# Supplementary material for: Transcriptional profiling of MnSOD-mediated lifespan extension in Drosophila reveals a species-general network of aging and metabolic genes
Source: Genome Biol. 2007 Dec 9;8(12):R262. doi: 10.1186/gb-2007-8-12-r262 (PMC2246264; doi:10.1186/gb-2007-8-12-r262)
Supplement: Additional data file 7 — Additional longevity promoting genes conserved between C. elegans daf-2 mutants and MnSOD over-expressing Drosophila. [file gb-2007-8-12-r262-S7.pdf]

D. melanogaster	***C. elegans***
**Gene / CG**	**CGC / GeneSeq**
**Up-regulated bv MnSOD: Same chronological age**	**Up-regulated in *daf-2* mutants**
**Synaptobrevin**	**Synaptobrevin**
n-syb / CG17248	***snb-1* / T10H9.4
**Jun-related antigen**	**Transcriptional activator of the JUN family**
Jra / CG2275	*T24H10.2
**Glutathione peroxidase**	**Glutathione peroxidase**
PHGPx / CG12013	**T09A12.2
**Heterogeneous nuclear ribonucleoprotein**	**Heterogeneous nuclear ribonucleoprotein**
Hrb98DE / CG9983	***msi-1* / R10E9.1
**Uncharacterized**	**Predicted leucine zipper transcription factors**
CG15099	***pad-1* / Y18D10A.13
**Catalase activity**	**Catalase (peroxisomal, cytosolic)**
kitty (kit) / CG9314	****ctl-2* / Y54G11A.5b, ****ctl-1* / Y54G11A.6
**Up-regulated bv MnSOD: Same physiological age**	**Up-regulated in *daf-2* mutants**
**Ortholog of the serine/threonine kinase Akt/PKB**	**Ortholog of the serine/threonine kinase Akt/PKB**
Akt1 / CG4006	****akt-1* / C12D8.10B
**Ankryin**	**Ankyrin-like protein**
Ank / CG1651	****unc-44* / B0350.2A
**Oxysterol binding**	**Oxysterol-binding protein**
CG3860	****obr-2* / F14H8.1
**Uncharacterized**	**Similar to mouse MAPK binding protein**
CG7337	**H24G06.1
**Sterol O-acyltransferase activity**	**Acyl-CoA:diacylglycerol o-acyltransferase activity**
CG8112	**H19N07.4
**Actin monomer binding**	**Thymosin beta ortholog; actin polymerization**
cib / CG4944	***tth-1* / F08F1.8
**Cytochrome P450 monooxygenase**	**Cytochrome P450 CYP4/CYP19/CYP26 subfamilies**
Cyp6a13 / CG2397	*cyp-29A2* / T19B10.1
**Beta-carotene 15,15'-monooxygenase activity**	**Beta, beta-carotene 15,15'-dioxygenase**
ninaB / CG9347	**F53C3.12
**Ras GTPase**	**Ras GTPase**
Ras64B	***ras-1* / C44C11.1
**Synaptotagmin**	**Synaptotagmin**
syt	****snt-1* / F31E8.2

Additional data file 7: Additional longevity promoting genes conserved between *C. elegans daf-2* mutants and MnSOD over-expressing *Drosophila*. *Drosophila* and *C. elegans* ortholog matches that are differentially expressed in response to MnSOD over-expression (at only one time point) and in *daf-2* mutants in a *daf-16* dependent manner. Expected values from BLASTP are indicated as follows: * $5 \times 10^{-10} < p \leq 5 \times 10^{-02}$, ** $5 \times 10^{-70} < p \leq 5 \times 10^{-10}$, *** $p \leq 5 \times 10^{-70}$. Beige shading indicates genes that are not reciprocal best BLAST hits, but are members of the corresponding gene family.
