# Supplementary material for: Transcriptional profiling of MnSOD-mediated lifespan extension in Drosophila reveals a species-general network of aging and metabolic genes
Source: Genome Biol. 2007 Dec 9;8(12):R262. doi: 10.1186/gb-2007-8-12-r262 (PMC2246264; doi:10.1186/gb-2007-8-12-r262)
Supplement: Additional data file 8 — MnSOD-regulated xenobiotic detoxification genes. [file gb-2007-8-12-r262-S8.pdf]

GO ID	Function Name	Genes	Count	Ref	*p-val*
**GO: 0008150**	**biological process**				
GO:0008152	metabolism	PGRP-SD; Cyp12a4; CG3011; CG6687; wal; PHGPx; ade2; Cyp12d1-p; Tal; CG6214; NA; Nmdmc; CG10444; CG3036; tok; ade3; CRMP; CG10960; eas; CG11796; Gyk; Ance-4; Cyp6d5; CG8132; pug; Got1; Hn; CG12428; fbp; CG9009; ade5; CG5493; CG2789; CG5567; Pcaf; CG11963; ref(2)P; CG6045; PGRP-LB; CG10638; CG32549; GstE7; CG17836; CG11899; GstE1	45	3157	0.005
GO:0006807	nitrogen compound metabolism	Got1; CG3011; CG12428; eas; CG11796; Hn; CG8132; pug; Nmdmc; CG5493; CG11899;	11	260	0.002
GO:0044271	nitrogen compound biosynthesis	Got1; CG11899; CG3011; eas; Nmdmc; pug	6	55	0.001
GO:0009308	amine metabolism	Got1; CG3011; CG12428; eas; CG11796; Nmdmc; pug; CG5493; Hn; CG11899;	10	247	0.004
GO:0009309	amine biosynthesis	Got1; CG11899; CG3011; eas; CG11796; CG5493; Hn; CG11899;	6	55	0.001
GO:0006519	amino acid and derivative metabolism	Got1; CG3011; CG12428; eas; CG11796; pug; CG5493; CG11899; Nmdmc;	10	191	0.001
GO:0006520	amino acid metabolism	Got1; CG3011; CG12428; CG11796; CG5493; Hn; CG11899; Nmdmc; pug	9	173	0.002
GO:0008652	amino acid biosynthesis	Got1; CG11899; CG3011; Nmdmc; pug	5	47	0.003
GO:0019752	carboxylic acid metabolism	Got1; CG3011; CG12428; CG9009; CG11796; Nmdmc; pug; CG5493; Hn; CG11899;	10	252	0.004
GO:0006725	aromatic compound metabolism	CG6045; CG32549; CG11796; ade5; Hn; Nmdmc; pug	9	92	1.5×10^{-4}
GO:0046483	heterocycle metabolism	ade5; CG6045; ade2; ade3; CG32549; Nmdmc; pug	7	91	0.001
GO:0006144	purine base metabolism	ade5; CG6045; ade2; ade3; CG32549	5	36	0.001
GO:0009127	purine nucleoside monophosphate biosynthesis	ade5; ade2; ade3	3	11	0.004
GO:0009168	purine ribonucleoside monophosphate biosynthesis	ade5; ade2; ade3	3	11	0.004
GO:0006188	IMP biosynthesis	ade5; ade2; ade3	3	7	0.001
GO:0006732	coenzyme metabolism	CG10444; CG2789; Tal; CG6687; Nmdmc; CG11963; pug	7	145	0.008
GO:0009396	folic acid and derivative biosynthesis	Nmdmc; pug	2	2	0.003
**GO: 0003674**	**molecular function**				
GO:0003824	catalytic activity	PGRP-SD; Cyp12a4; CG3011; CG4716; PHGPx; Cyp12d1-p; CG6687;	40	2375	0.001
GO:0016491	oxidoreductase activity;	Nmdmc; ade2; CRMP; eas; CG11796; Gyk; Tal; CG8132; Cyp6d5; wal; CG11899; GstE1 pug; Got1; tok; CG1681; ade3; CG12428; fbp; CG9009; ade5; CG6045; PGRP-LB; Pcaf; CG5493; CG5567; ref(2)P; CG11963; CG10638; CG32549; CG6214; GstE7; Hn;	14	401	0.001
GO:0016645	oxidoreductase activity, acting on the CH-NH group of donors	CG4716; Nmdmc; pug	3	12	0.004
GO:0051213	dioxygenase activity	CG11796; CG5493	2	4	0.008
GO:0004486	methylenetetrahydrofolate dehydrogenase activity	CG4716; Nmdmc; pug	3	3	2.8×10^{-4}
GO:0016810	hydrolase activity	PGRP-SD; PGRP-LB; CRMP; CG8132; Nmdmc; pug	6	53	0.001
GO:0019238	cyclohydrolase activity	Nmdmc; pug	2	3	0.005
GO:0004372	transferase activity	ade3; CG3011	2	3	0.005

Additional data file 8: MnSOD-regulated xenobiotic detoxification genes. Gene Ontology classifications and functional overrepresentation of MnSOD up-regulated genes that are also up-regulated due to the xenobiotic response to phenobarbital.
