# Supplementary material for: Transcriptional profiling of MnSOD-mediated lifespan extension in Drosophila reveals a species-general network of aging and metabolic genes
Source: Genome Biol. 2007 Dec 9;8(12):R262. doi: 10.1186/gb-2007-8-12-r262 (PMC2246264; doi:10.1186/gb-2007-8-12-r262)
Supplement: Additional data file 9 — DNA regulatory elements in the set of conserved longevity promoting genes. [file gb-2007-8-12-r262-S9.pdf]

WormBase ID	Flybase ID	CG	HRE	ARE	DRE	DBE	DAE	HIF-RE
H42K12.1	*Pk61C*	CG1210	X			X	X	
Y51H4A.17	*Stat92E*	CG4257	X	X		X	X	
Y54G2A.18	CG13887	CG13887				X		
F26D10.9	CG14935	CG14935				X		
C37H5.6	CG17273	CG17273				X	X	
B0286.3	*ade5*	CG3989				X		
F55F3.1	CG8057	CG8057				X	X	
C04F12.8	*puc*	CG7850				X	X	
F21A3.2	CG1637	CG1637	X		X			
T19B10.3	*Ect3*	CG3132						
F38B6.4	*ade3*	CG31628	X	X			X	X
C55F2.1	CG11089	CG11089					X	
ZK662.3	*Hr96*	CG11783						
M01F1.4	CG2698	CG2698						
C41G7.3	CG2789	CG2789						
F53F10.4	*Rab2*	CG3269			X		X	
B0228.5	*TrxT*	CG3315			X			
F47B7.2	CG4670	CG4670						
W03C9.3	*Rab7*	CG5915			X			
K10B3.8	CG8893	CG8893						
K07E3.8	CG9066	CG9066						
Y48G8AL.1	CG9153	CG9153						
R08C7.2	CG9947	CG9947			X		X	
T07C4.9	*Anxb11*	CG9968		X				
F43E2.2	*Ada2b*	CG9638		X				
R10E9.1	*Hrb98DE*	CG9983						
T24H10.2	*Jra*	CG2275			X			
T09A12.2	*PHGPx*	CG12013						
T10H9.4	*n-syb*	CG17248						
Y18D10A.13	CG15099	CG15099			X	X		
C12D8.10B	*Akt1*	CG4006	X			X	X	
B0350.2A	*Ank*	CG1651	X				X	
F14H8.1	CG3860	CG3860	X				X	
H24G06.1	CG7337	CG7337	X	X	X	X	X	
H19N07.4	CG8112	CG8112	X	X				
F08F1.8	*cib*	CG4944	X	X			X	
T19B10.1	*Cyp6a13*	CG2397				X		
F53C3.12	*ninaB*	CG9347	X	X				
C44C11.1	*Ras64B*	CG1167	X	X	X	X		
F31E8.2	*syt*	CG3139		X			X	

Additional data file 9: DNA regulatory elements in the set of conserved longevity promoting genes. The presence of various DNA regulatory elements in the set of conserved longevity promoting genes for which the significance associated with finding that motif had a *p*-value < 0.05 is indicated by an “×”. For each gene, the 2 Kb upstream region and first intron were queried for the presence of the hydrogen peroxide response element (HRE), antioxidant response element (ARE), DNA replication element (DRE), DAF-16 binding element (DBE), DAF-16 associated element (DAE), and hypoxia induction factor (HIF-1) response element (HIF-RE). Light or dark grey shading indicates genes that were only altered at the first time point (same chronological age) or at the second time point (same “physiological age”), respectively.
