# Supplementary material for: Transcriptional profiling of MnSOD-mediated lifespan extension in Drosophila reveals a species-general network of aging and metabolic genes
Source: Genome Biol. 2007 Dec 9;8(12):R262. doi: 10.1186/gb-2007-8-12-r262 (PMC2246264; doi:10.1186/gb-2007-8-12-r262)
Supplement: Additional data file 10 — Supporting results and additional explanatory text. [file gb-2007-8-12-r262-S10.pdf]

Supporting results and additional explanatory text

Discussion of control lifespan

It should be noted that the lifespan of the controls used here (mean lifespan ~73 days at 25°C, Table S1C) compare favorably to the extended mutant lifespans reported for *InR* (60 days), JNK pathway (65 days), *chico* (65 days), *dTOR* (72 days) and *Methuselah* (77 days) [1-4]. Therefore, it is unlikely that MnSOD over-expression rescues some defect specific to the strains used. Preliminary data suggest that there is a limit to the amount of lifespan extension that can be achieved by over-expression of MnSOD alone: MnSOD transcript levels have been further increased by combining two MnSOD transgenic target constructs and/or by using a more active rtTA transactivator line [5], however this has so far yielded negative effects on lifespan [6]. Greater increases in life span (+40%) have been achieved by combining MnSOD with other lifespan-extending genes such as Cu/ZnSOD [7].

Cross-species, cross-condition comparisons reveals shared longevity gene-expression signatures

Comparison of the transcriptional profiles of MnSOD over-expression in *Drosophila* to those to those of long-lived *daf-2* mutants revealed a suite of genes with similar expression patterns in these two long-lived organisms. That these genes were altered in multiple conditions and different organisms suggests that they may play a significant role in mediating longevity. Some of these conserved, longevity promoting genes are described in greater detail below.

Hr96 and CG9066

Hr96 encodes a nuclear receptor represented by an orthologous group of three *C. elegans* genes: *daf-12, nhr-8,* and *nhr-48* [8]. NHR-8 is implicated in xenobiotic stress [9], while DAF-12 functions in dauer arrest, lipid metabolism, insulin signaling, and longevity [10-12]. Several vertebrate nuclear receptors play a central role in xenobiotic responses by directly binding toxic compounds and inducing the expression of key detoxification enzymes such as cytochrome P450s monooxygenases (P450s) and glutathione s-transferases (GSTs) [13]. Recently, numerous studies aimed at understanding xenobiotic responses in *Drosophila* [14-17] have shown that HR96 is functionally similar to the human receptors SXR and CAR, being required for proper xenobiotic responses in *Drosophila* [14, 15]. Additional studies revealed the differential expression of detoxification enzymes in male and female *Drosophila* upon treatment with PB, and that xenobiotics can alter hormonally regulated physiological processes [17].

The functions of the *C. elegans* and vertebrate HR96 homologues suggest that in *Drosophila* this nuclear receptor may function not only as a xenobiotic stress sensor responsible for regulating detoxification pathways [9], but also as a sensor of steroid hormones. In *C. elegans,* DAF-12 functions to integrate hormonal signals that determine major life history traits [10] and to modulate stress responses downstream of *daf-16*. Recently, DAF-12 has been further characterized revealing a complex mode of regulation and alleles that can extend or shorten lifespan [11]. Several ligands for the receptor have now been identified including dafachronic acids [18], cholestenoic acids [19], and the steroid pregnenalone (PREG) [20]. PREG can extend lifespan and levels of this molecule

are increased in germline defective *daf-9* mutants, while it has no effect on *daf-12* mutants [20].

Endocrine signals have been demonstrated to regulate life cycles and aging in all higher organisms [21, 22]. In *Drosophila*, the importance of JH in longevity modulation via endocrine signaling has been established [2, 22, 23]. However, details concerning aspects of the mechanism remain to be elucidated and other hormones are likely involved since some InR mutants that appear to lack JH are not long-lived [24]. The steroid prohormone ecdysone also influences both reproduction and longevity [25, 26]. The majority of 20-hydroxyecdysone (20HE) effects are processed through the ecdysone-ultraspinacle (EcR-USP) heterodimer receptor that transcriptionally regulates downstream gene expression [9]. Notably, HR96 is also 20HE inducible, and its encoded protein binds selectively to the *hsp27* 20HE response element. Furthermore, the 20HE receptor can bind to each of the sequences recognized by both HR96 and HR78, suggesting that these proteins may compete with the receptor for binding to a common set of target sequences [27].

Previous studies indicate that both JH and 20HE are decreased in IIS mutants [2] and that *EcR* mutant heterozygotes are long-lived [28] suggesting that reduced IIS activity in *Drosophila* may extend lifespan by diminishing signaling through JH and ecdysone. The finding that expression levels of *EcR* and numerous other genes involved in endocrine signaling are down-regulated in response to MnSOD over-expression in transgenic flies relative to controls of the same "physiological age" is therefore of interest. Additionally, the molecular chaperones *Hsc70* and *Hsp90*, which are implicated in longevity and

interact with EcR-USP [22, 25, 29], were also up-regulated by MnSOD (at both time points) as was the gene encoding heat shock factor (HSF). Given that MnSOD over-expressing flies did not demonstrate increased thermotolerance, these genes may be induced for alternative purposes, perhaps functioning in the endocrine regulation of aging. In support of this view, *C. elegans* heat shock factor HSF-1 extends lifespan in a cell-nonautonomous manner in *daf-2* mutants [30] and thus likely has additional functions besides the regulation of cell-autonomous heat shock genes [3].

Another gene involved in hormone secretion (GO:0046879), CG9066, was also amongst the conserved targets identified in this analysis. CG9066 has a cytochrome b5 domain and is orthologous to the human membrane associated progesterone receptor and the *C. elegans vem-1* gene (K07E3.8), a putative steroid membrane receptor which functions in the nematode xenobiotic response [31].

Previously, it has been suggested that secondary hormones act downstream of IIS to modulate reproduction and lifespan in long-lived IIS mutants [2], and that JH and ecdysone may represent these hormones acting in an analogous manner to the *daf-12* dependent steroid hormones in *C. elegans* [22]. Importantly, this possibility remains in question due to the fact that the sterility of *chico* mutant female *Drosophila* is both autonomous to the ovary and independent of JH and ecdysone levels [32]. Although a role for HR96 in insulin signaling has not been established, it may function similarly to *C. elegans* DAF-12 to process hormonal cues in the form of lipophillic molecules that could include JH or 20HE with effects on longevity and reproduction. A role for CG9066

acting upstream or in concert with HR96 in the endocrine pathway is also plausible. Furthermore, the finding that PB-treated HR96 mutants demonstrate increased expression of four genes encoding juvenile hormone binding proteins (JHBPs) [15] lends support to this view since it suggests that HR96 is normally required for their repression. JHBPs are important for the transport of JH from the hemolymph to specific target tissues [33]. One model for HR96 in the endocrine regulation of aging might involve a feedback loop such that the repression of JHBPs by HR96 results in reduced targeting of JH to its receptor in specific tissues thereby activating a pro-longevity mode. The juvenile hormone inducible protein JhI-26 may also participate in this pathway to promote longevity since its expression is up-regulated due to altered IIS activity in *Drosophila* and in response to MnSOD over-expression (discussed below). A schematic depicting the possible role of HR96 in the endocrine regulation of aging is shown in Additional data file 11.

Carbohydrate metabolism genes

Several carbohydrate metabolism genes were amongst those altered by MnSOD and in *C. elegans* IIS mutants. These include alpha amylase (CG14935), glucosidase (CG46700), beta-galactosidase (*Ect3*), and glyceraldehyde-3-phosphate dehydrogenase (*Gapdh2*).

Purine biosynthesis genes

Four components of the purine nucleotide biosynthesis pathway (*ade3, ade5*, CG17273, CG11089) were amongst those identified in this comparison (GO:0006164; $p < 0.05$). This is interesting in light of the previous finding that components of the purine pathway are up-regulated in aged and oxidatively stressed flies [34]. In further support of purines

serving as a form of metabolic currency, Bauer *et al.* recently noted that purine pathway genes are highly down-regulated in males as compared to females faced with nutrient deprivation suggesting a possible role in the tradeoff between somatic repair, reproduction, and longevity

Cytoprotective genes

Numerous genes (*Stat92E, Pk61C, Rab7, Ect3,* CG13887) involved in programmed cell death (GO:0012501; $p < 0.05$) were identified as potential longevity promoting genes suggesting an important role for cytoprotection. In addition to participating in apoptosis, these genes also carry out other important functions. For example, *Pk61C* functions upstream of *Akt1* in the IIS pathway. *Stat92E* plays a crucial role in various cellular processes ranging from the immune response to cell cycle control and cell growth. Ect3 functions in salivary gland cell death while CG13877 is involved in the defense response as is thioredoxin (*TrxT*). CG9153 encodes an E3 ubiquitin protein ligase, and thus assists in targeting damaged or unfolded proteins to the proteasome for degradation.

CG2698

Although CG2698 and its orthologous worm gene (M01F1.4) are uncharacterized, M01F1.4 encodes a gene with a metabotropic gamma-aminobutyric acid (GABA) receptor type B1 domain (IPR002456). GABA is the principal inhibitory neurotransmitter and Girardot *et al.* [35] have reported the age-related impairment of synaptic transmission gene expression in the brain including a member of this receptor family. GABA B type receptor activation has also been demonstrated to inhibit insulin secretion in rat

pancreatic beta cells in an autocrine fashion by the direct inhibition of exocytosis [36].

Rab7

Amongst the conserved longevity promoting genes, the small GTPases *Rab2* and *Rab7* both function in intracellular transport as does CG13887. In particular, *Rab7* participates in transport from the late endosome to the lysosome and in the maturation of late autophagic vesicles [37]. A role for *Rab7* in insulin signaling and DR has recently been suggested based upon the finding that the localization of both GFP-Rab7 and clathrin were altered in response to TOR inhibition [38]. Studies in model organisms indicate that reduced signaling through the TOR kinase extends lifespan via the up-regulation of a highly conserved response during nutrient restriction [39].

MnSOD-regulated targets downstream of dFOXO

The cross-species, cross-condition comparison described in the above was aimed at identifying genes and processes that broadly mediate lifespan and hence are robust signatures of longevity mechanisms. However, certain downstream targets of dFOXO/DAF-16 may have been missed by a comparison of stringent orthologs. In order to identify species specific MnSOD-regulated targets that act downstream of dFOXO as well as potential lifespan promoting mechanisms that might be unique to *Drosophila*, the transcriptional profile of MnSOD over-expression was compared to those resulting from altered insulin signaling in *Drosophila*.

Although *Drosophila InR* [2] and *chico* [1] mutants have been described, their transcriptional outputs have not as of the time of the preparation of this manuscript. As such, comparisons were made to the limited available datasets. A comparison of the patterns of expression due to MnSOD over-expression and insulin stimulation of S2 cells expressing constitutively active dFOXO [40] revealed 59 genes ($p << 0.001$) up-regulated in response to both interventions. Only three of these genes were also amongst the conserved longevity promoting genes also identified in worms, namely, Ect3, *Anxb11*, and *Pk61C*. Amongst these 59 genes, 11 were also down-regulated upon insulin stimulation of *Drosophila* Kc167 [41] cells, but only Pk61C was included amongst the conserved worm genes. These insulin signaling studies differ from that described above in *C. elegans* since they did not employ flies mutant for the *Drosophila InR* or insulin-like receptor substrate (IRS), *chico*, but simply insulin stimulation of a wild-type cell line and one expressing activated dFOXO. It should also be noted that since these studies profiled the transcriptome of two different types of tissue culture cells rather than the whole adult male transcriptome (as in this study), cell type specific differences can be expected.

The transcriptional profiles resulting from MnSOD over-expression were also compared to those of Gershman *et al.* [42] who examined the transcriptional profiles resulting from yeast re-feeding after DR in female flies and also considered the overlap with the above mentioned dFOXO study. Notably, DR did not prolong longevity in this study [43]. Although the adults from yeast-deprived larvae demonstrated reduced fecundity and small body size (similar to *InR* and *chico* mutants), they had normal patterns of

senescence, accompanied by normal ILP and JH synthesis [43]. Since yeast deprivation inhibits ILP secretion [43] genes contributing to dFOXO mediated longevity would be expected to be down-regulated in this intervention. A comparison of MnSOD up-regulated genes (same chronological age) to those down-regulated by reintroduction of yeast in the diet yielded an overlap of 151 genes ($p << 0.001$) many of which were also identified in the comparisons to other interventions. In particular, 22 such genes were also up-regulated in FOXOA3 cells, including *Pk61c, JhI-26, Rab1, Fer2LCH, Chmp1, scyl,* and suggesting an important role for these genes in modulating *Drosophila* aging in response to diverse interventions. Notably, the AMPK encoding gene, CG8057, was identified in this overlap, but not in the comparison to FOXOA3 cells. A comparison of genes up-regulated by MnSOD and by the reintroduction of yeast in the diet also yielded a large (135 genes) overlap ($p < 0.001$) suggesting that various processes are altered in an opposing manner in these two conditions.

Given the numerous differences in the conditions being assayed and the experimental systems used (such as male versus female, whole tissue versus cell lines), it is noteworthy that five genes (*Pk61C,* CG13624, CG30015, *vir-1*, *JhI-26*) exhibited altered expression in each of the conditions considered. *Pk61C* has been mentioned previously and will not be further described. CG13624 encodes a transcription factor with a basic leucine zipper domain and participates in protein homodimerization though little is known of its interaction partners. The virus-induced RNA 1 gene, *vir-1*, and CG30015 are of unknown function.

The genes encoding the juvenile hormone inducible proteins *JhI-26* and *JhI-1* were previously found to be transcriptionally up-regulated upon RNAi knockdown of SIN3A in *Drosophila* tissue culture cells [44]. This study also revealed that the SIN3 histone deacetylase complex not only functions as a co-repressor for nuclear hormone receptors, but also regulates a substantial portion of genes involved in mitochondrial energy generation including oxidative phosphorylation and the detoxification of ROS [44]. The up-regulation of *JhI-26* due to altered insulin signaling and its induction by xenobiotic stress and MnSOD over-expression suggest a role for this gene in longevity determination.

References

1. Clancy DJ, Gems D, Harshman LG, Oldham S, Stocker H, Hafen E, Leevers SJ, Partridge L: **Extension of life-span by loss of CHICO, a Drosophila insulin receptor substrate protein**. *Science* 2001, **292**(5514):104-106.
2. Tatar M, Kopelman A, Epstein D, Tu MP, Yin CM, Garofalo RS: **A mutant Drosophila insulin receptor homolog that extends life-span and impairs neuroendocrine function**. *Science* 2001, **292**(5514):107-110.
3. Kenyon C: **The plasticity of aging: insights from long-lived mutants**. *Cell* 2005, **120**(4):449-460.
4. Landis GN, Tower J: **Superoxide dismutase evolution and life span regulation**. *Mech Ageing Dev* 2005, **126**(3):365-379.
5. Bieschke ET, Wheeler JC, Tower J: **Doxycycline-induced transgene expression during Drosophila development and aging**. *Mol Gen Genet* 1998, **258**(6):571-579.
6. Ford D, Hoe N, Landis G, Tozer K, Luu A, Bhole D, Badrinath A, Tower J: **Alteration of Drosophila life span using conditional, tissue-specific expression of transgenes triggered by doxycyline or RU486/Mifepristone**. *Exp Gerontol*, in press.
7. Sun J, Molitor J, Tower J: **Effects of simultaneous over-expression of Cu/ZnSOD and MnSOD on Drosophila melanogaster life span**. *Mech Ageing Dev* 2004, **125**(5):341-349.
8. Snow MI, Larsen PL: **Structure and expression of daf-12: a nuclear hormone receptor with three isoforms that are involved in development and aging in**

Caenorhabditis elegans. *Biochimica et biophysica acta* 2000, **1494**(1-2):104-116.
9. King-Jones K, Thummel CS: **Nuclear receptors--a perspective from Drosophila**. *Nat Rev Genet* 2005, **6**(4):311-323.
10. Antebi A, Yeh WH, Tait D, Hedgecock EM, Riddle DL: **daf-12 encodes a nuclear receptor that regulates the dauer diapause and developmental age in C. elegans**. *Genes Dev* 2000, **14**(12):1512-1527.
11. Fisher AL, Lithgow GJ: **The nuclear hormone receptor DAF-12 has opposing effects on Caenorhabditis elegans lifespan and regulates genes repressed in multiple long-lived worms**. *Aging Cell* 2006, **5**(2):127-138.
12. Ludewig AH, Kober-Eisermann C, Weitzel C, Bethke A, Neubert K, Gerisch B, Hutter H, Antebi A: **A novel nuclear receptor/coregulator complex controls C. elegans lipid metabolism, larval development, and aging**. *Genes Dev* 2004, **18**(17):2120-2133.
13. Willson TM, Kliewer SA: **PXR, CAR and drug metabolism**. *Nat Rev Drug Discov* 2002, **1**(4):259-266.
14. Palanker L, Necakov AS, Sampson HM, Ni R, Hu C, Thummel CS, Krause HM: **Dynamic regulation of Drosophila nuclear receptor activity in vivo**. *Development* 2006, **133**(18):3549-3562.
15. King-Jones K, Horner MA, Lam G, Thummel CS: **The DHR96 nuclear receptor regulates xenobiotic responses in Drosophila**. *Cell Metab* 2006, **4**(1):37-48.
16. Sun W, Margam VM, Sun L, Buczkowski G, Bennett GW, Schemerhorn B, Muir WM, Pittendrigh BR: **Genome-wide analysis of phenobarbital-inducible genes in Drosophila melanogaster**. *Insect Mol Biol* 2006, **15**(4):455-464.
17. Le Goff G, Hilliou F, Siegfried BD, Boundy S, Wajnberg E, Sofer L, Audant P, ffrench-Constant RH, Feyereisen R: **Xenobiotic response in Drosophila melanogaster: sex dependence of P450 and GST gene induction**. *Insect Biochem Mol Biol* 2006, **36**(8):674-682.
18. Motola DL, Cummins CL, Rottiers V, Sharma KK, Li T, Li Y, Suino-Powell K, Xu HE, Auchus RJ, Antebi A *et al*: **Identification of ligands for DAF-12 that govern dauer formation and reproduction in C. elegans**. *Cell* 2006, **124**(6):1209-1223.
19. Held JM, White MP, Fisher AL, Gibson BW, Lithgow GJ, Gill MS: **DAF-12-dependent rescue of dauer formation in Caenorhabditis elegans by (25S)-cholestenoic acid**. *Aging Cell* 2006, **5**(4):283-291.
20. Broue F, Liere P, Kenyon C, Baulieu EE: **A steroid hormone that extends the lifespan of Caenorhabditis elegans**. *Aging Cell* 2007, **6**(1):87-94.
21. Pardee K, Reinking J, Krause H: **Nuclear hormone receptors, metabolism, and aging: what goes around comes around.Transcription factors link lipid metabolism and aging-related processes**. *Sci Aging Knowledge Environ* 2004, **2004**(47):re8.
22. Tatar M, Bartke A, Antebi A: **The endocrine regulation of aging by insulin-like signals**. *Science* 2003, **299**(5611):1346-1351.
23. Tu MP, Yin CM, Tatar M: **Mutations in insulin signaling pathway alter juvenile hormone synthesis in Drosophila melanogaster**. *General and comparative endocrinology* 2005, **142**(3):347-356.

24. Kenyon C: **A conserved regulatory system for aging**. *Cell* 2001, **105**(2):165-168.
25. Tsai CC, Kao HY, Yao TP, McKeown M, Evans RM: **SMRTER, a Drosophila nuclear receptor coregulator, reveals that EcR-mediated repression is critical for development**. *Molecular cell* 1999, **4**(2):175-186.
26. Colombani J, Bianchini L, Layalle S, Pondeville E, Dauphin-Villemant C, Antoniewski C, Carre C, Noselli S, Leopold P: **Antagonistic actions of ecdysone and insulins determine final size in Drosophila**. *Science* 2005, **310**(5748):667-670.
27. Thummel CS: **From embryogenesis to metamorphosis: the regulation and function of Drosophila nuclear receptor superfamily members**. *Cell* 1995, **83**(6):871-877.
28. Simon AF, Shih C, Mack A, Benzer S: **Steroid control of longevity in Drosophila melanogaster**. *Science* 2003, **299**(5611):1407-1410.
29. Arbeitman MN, Hogness DS: **Molecular chaperones activate the Drosophila ecdysone receptor, an RXR heterodimer**. *Cell* 2000, **101**(1):67-77.
30. Morley JF, Morimoto RI: **Regulation of longevity in Caenorhabditis elegans by heat shock factor and molecular chaperones**. *Molecular biology of the cell* 2004, **15**(2):657-664.
31. Reichert K, Menzel R: **Expression profiling of five different xenobiotics using a Caenorhabditis elegans whole genome microarray**. *Chemosphere* 2005, **61**(2):229-237.
32. Richard DS, Rybczynski R, Wilson TG, Wang Y, Wayne ML, Zhou Y, Partridge L, Harshman LG: **Insulin signaling is necessary for vitellogenesis in Drosophila melanogaster independent of the roles of juvenile hormone and ecdysteroids: female sterility of the chico1 insulin signaling mutation is autonomous to the ovary**. *Journal of insect physiology* 2005, **51**(4):455-464.
33. Braun RP, Wyatt GR: **Sequence of the hexameric juvenile hormone-binding protein from the hemolymph of Locusta migratoria**. *J Biol Chem* 1996, **271**(49):31756-31762.
34. Landis GN, Abdueva D, Skvortsov D, Yang J, Rabin BE, Carrick J, Tavare S, Tower J: **Similar gene expression patterns characterize aging and oxidative stress in Drosophila melanogaster**. *Proc Natl Acad Sci U S A* 2004, **101**(20):7663-7668.
35. Girardot F, Lasbleiz C, Monnier V, Tricoire H: **Specific age-related signatures in Drosophila body parts transcriptome**. *BMC genomics* 2006, **7**:69.
36. Braun M, Wendt A, Buschard K, Salehi A, Sewing S, Gromada J, Rorsman P: **GABAB receptor activation inhibits exocytosis in rat pancreatic beta-cells by G-protein-dependent activation of calcineurin**. *J Physiol* 2004, **559**(Pt 2):397-409.
37. Jager S, Bucci C, Tanida I, Ueno T, Kominami E, Saftig P, Eskelinen EL: **Role for Rab7 in maturation of late autophagic vacuoles**. *J Cell Sci* 2004, **117**(Pt 20):4837-4848.
38. Hennig KM, Colombani J, Neufeld TP: **TOR coordinates bulk and targeted endocytosis in the Drosophila melanogaster fat body to regulate cell growth**. *J Cell Biol* 2006, **173**(6):963-974.

39. Powers RW, 3rd, Kaeberlein M, Caldwell SD, Kennedy BK, Fields S: **Extension of chronological life span in yeast by decreased TOR pathway signaling**. *Genes Dev* 2006, **20**(2):174-184.
40. Puig O, Marr MT, Ruhf ML, Tjian R: **Control of cell number by Drosophila FOXO: downstream and feedback regulation of the insulin receptor pathway**. *Genes Dev* 2003, **17**(16):2006-2020.
41. Junger MA, Rintelen F, Stocker H, Wasserman JD, Vegh M, Radimerski T, Greenberg ME, Hafen E: **The Drosophila forkhead transcription factor FOXO mediates the reduction in cell number associated with reduced insulin signaling**. *J Biol* 2003, **2**(3):20.
42. Gershman B, Puig O, Hang L, Peitzsh RM, Tatar M, Garofalo RS: **High resolution dynamics of the transcriptional response to nutrition in Drosophila: a key role for dFOXO**. *Physiol Genomics* 2006.
43. Tu MP, Tatar M: **Juvenile diet restriction and the aging and reproduction of adult Drosophila melanogaster**. *Aging Cell* 2003, **2**(6):327-333.
44. Pile LA, Spellman PT, Katzenberger RJ, Wassarman DA: **The SIN3 deacetylase complex represses genes encoding mitochondrial proteins: implications for the regulation of energy metabolism**. *J Biol Chem* 2003, **278**(39):37840-37848.
