# Supplementary material for: Transcriptional profiling of MnSOD-mediated lifespan extension in Drosophila reveals a species-general network of aging and metabolic genes
Source: Genome Biol. 2007 Dec 9;8(12):R262. doi: 10.1186/gb-2007-8-12-r262 (PMC2246264; doi:10.1186/gb-2007-8-12-r262)
Supplement: Additional data file 11 — Proposed role for HR96 in the endocrine regulation of lifespan. [file gb-2007-8-12-r262-S11.pdf]

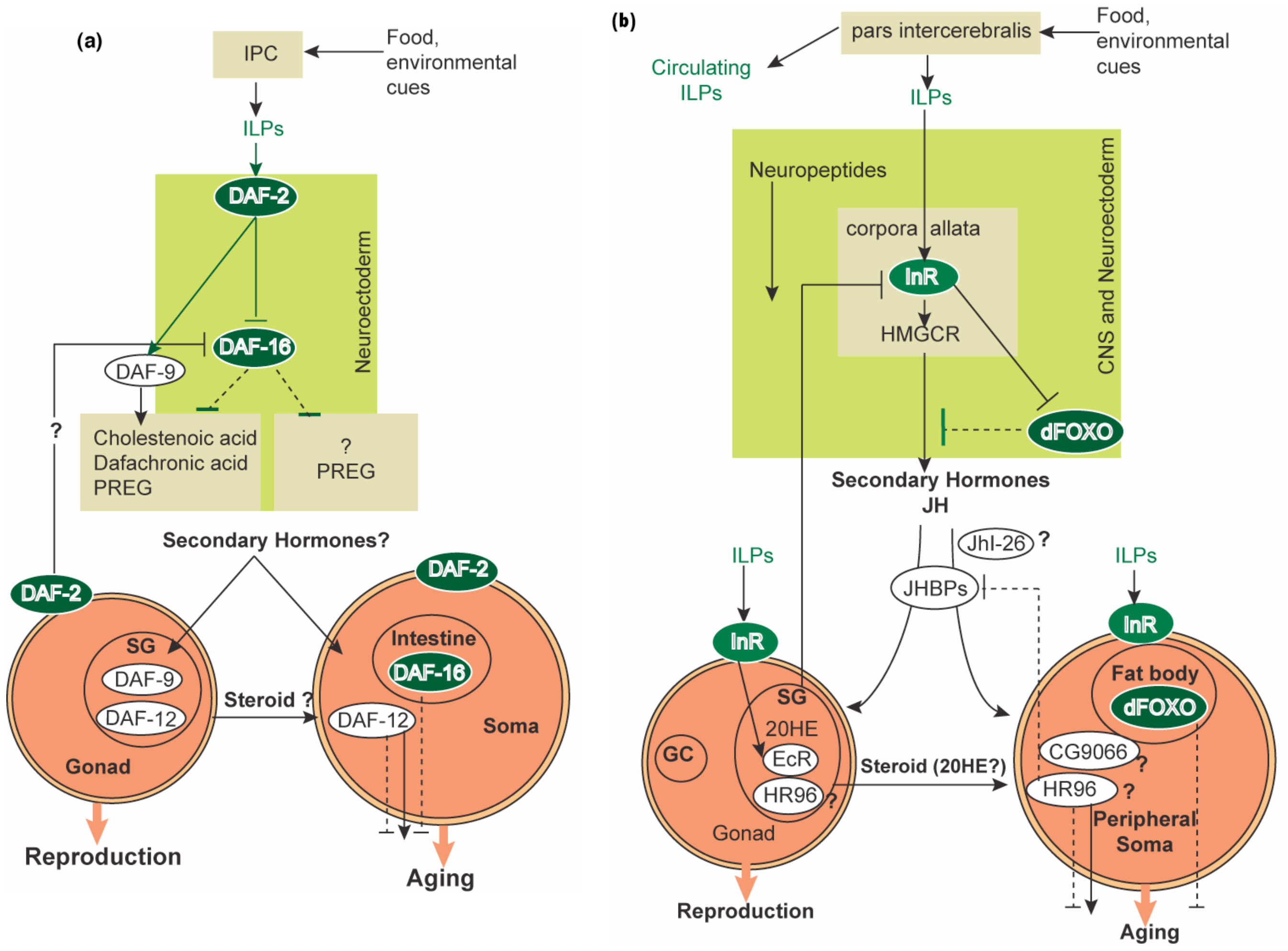

Additional data file 11: Proposed role for HR96 in the endocrine regulation of lifespan. Models for the endocrine regulation of aging in *C. elegans* and *D. melanogaster*. The CNS responds to environmental and dietary cues resuslting in activation of the reproductive, pro-aging mode via endocrine signaling. Pathways contributing to the active reproductive mode are denoted by solid lines whereas, broken lines indicate down-regulated pathways. IIS pathway components are shown in green. Note that arrows indicate activation whereas bars indicate inhibition. Hypothetical or speculative elements are denoted by a "?". (A) *C. elegans*. In response to environmental cues, insulin-like peptides (ILPs) are produced in the insulin producing cells (IPCs). DAF-2 becomes activated in target tissues, such as the neuroectoderm, resulting in putative secondary endocrines that stimulate reproductive mode such as Cholestenoic acid, Dafachronic acid, and PREG (pregnenolone). In the gonad, signals from the germ line cells (GC) inhibit production of a life-extending steroid produced by DAF-9, potentially synthesized in somatic gonad (SG) tissue. Secondary endocrines could inhibit the life-extending activities of DAF-16 and DAF-12 nuclear hormone receptor in multiple target or endocrine tissues. (B) *D. melanogaster*. In response to environmental cues, cells of the pars intercebralis produce ILPs. Insulin signaling stimulates juvenile hormone (JH) production in the corpora allata. This results in increased egg production and development in the gonad as well as, synthesis of 20-hydroxyecdysone (20HE). ILPs also act in the peripheral soma on the fat body to influence the rate of aging. Here, *Hr96* and

CG9066 are proposed to contribute to an anti-aging mode based on their identification as potential longevity promoting genes. Based on orthology to *C. elegans* DAF-12, HR96 may also integrate pro-reproductive signals in the gonad. Since juvenile hormone binding proteins (JHBPs) act as carriers for JH to its target tissues, the repression of JHBPs by HR96 might represent a feedback loop such that delivery of JH to its potential receptor in specific target tissues (such as the fat body) is inhibited. In the absence of ligand, reproductive mode would be inactive, while the pro-longevity mode would be active (dashed lines). *JhI-26* may also participate in this pathway to promote longevity since its expression is up-regulated due to altered IIS activity in *Drosophila* and in response to MnSOD over-expression. Adapted from Tatar *et al*., 2003 [22].
